# Control of Ionic Conductivity by Lithium Distribution in Cubic Oxide Argyrodites Li_6+*x*_P_1–*x*_Si_*x*_O_5_Cl

**DOI:** 10.1021/jacs.2c09863

**Published:** 2022-11-22

**Authors:** Alexandra Morscher, Benjamin B. Duff, Guopeng Han, Luke M. Daniels, Yun Dang, Marco Zanella, Manel Sonni, Ahmad Malik, Matthew S. Dyer, Ruiyong Chen, Frédéric Blanc, John B. Claridge, Matthew J. Rosseinsky

**Affiliations:** † Department of Chemistry, University of Liverpool, Crown Street, L69 7ZDLiverpool, U.K.; ‡ Stephenson Institute for Renewable Energy, University of Liverpool, Peach Street, L69 7ZFLiverpool, U.K.

## Abstract

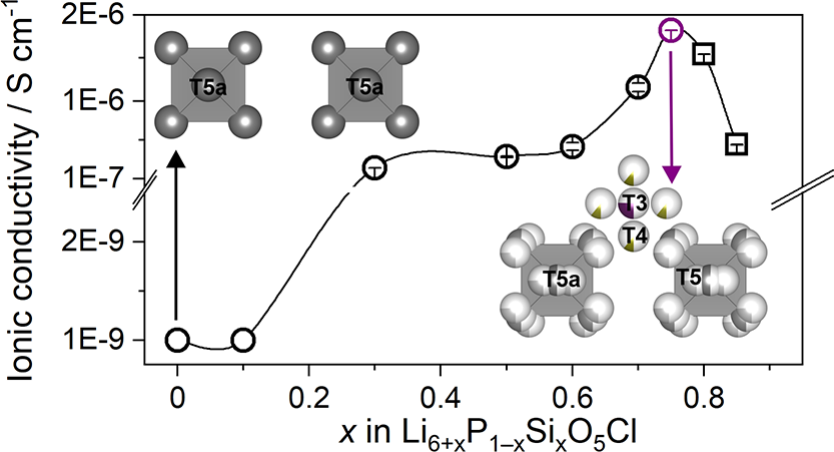

Argyrodite is a key structure type for ion-transporting materials. Oxide argyrodites are largely unexplored despite sulfide argyrodites being a leading family of solid-state lithium-ion conductors, in which the control of lithium distribution over a wide range of available sites strongly influences the conductivity. We present a new cubic Li-rich (>6 Li^+^ per formula unit) oxide argyrodite Li_7_SiO_5_Cl that crystallizes with an ordered cubic (*P*2_1_3) structure at room temperature, undergoing a transition at 473 K to a Li^+^ site disordered *F*4̅3*m* structure, consistent with the symmetry adopted by superionic sulfide argyrodites. Four different Li^+^ sites are occupied in Li_7_SiO_5_Cl (T5, T5a, T3, and T4), the combination of which is previously unreported for Li-containing argyrodites. The disordered *F*4̅3*m* structure is stabilized to room temperature via substitution of Si^4+^ with P^5+^ in Li_6+*x*_P_1–*x*_Si_*x*_O_5_Cl (0.3 < *x* < 0.85) solid solution. The resulting delocalization of Li^+^ sites leads to a maximum ionic conductivity of 1.82(1) × 10^–6^ S cm^–1^ at *x* = 0.75, which is 3 orders of magnitude higher than the conductivities reported previously for oxide argyrodites. The variation of ionic conductivity with composition in Li_6+*x*_P_1–*x*_Si_*x*_O_5_Cl is directly connected to structural changes occurring within the Li^+^ sublattice. These materials present superior atmospheric stability over analogous sulfide argyrodites and are stable against Li metal. The ability to control the ionic conductivity through structure and composition emphasizes the advances that can be made with further research in the open field of oxide argyrodites.

## 1 Introduction

All-solid-state batteries (ASSBs) replace the flammable liquid electrolyte currently used in most commercial Li^+^-ion batteries (LIBs) with a solid-state lithium electrolyte. As such, they offer the prospect of increased safety, increased power and energy densities, and longer lifetimes when compared to conventional LIBs.^1^ ASSBs have already found use in thin-film batteries in which conductivities in the range of 10^–6^ S cm^–1^ are sufficient for device operation. For example, the most widely used thin-film solid electrolyte is the glassy lithium phosphorous oxynitride (LIPON) with a room-temperature conductivity of ∼10^–6^ S cm^–1^.^2,3^ Several oxide solid lithium electrolytes are used as protective coatings to enable compatibility with electrodes,^4^ including Li_2_SiO_3_, Li_4_Ti_5_O_12_, LiTaO_3_, Li_3_PO_4_, and LiNbO_3_, with ionic conductivities ranging from 10^–5^ S cm^–1^ (amorphous LiNbO_3_) to 10^–8^ S cm^–1^ (Li_4_Ti_5_O_12_).^4^

One of the most promising classes of solid electrolytes is the argyrodite family which exhibits superionic conductivities at room temperature, for example, Li_6_PS_5_Br (6.8 × 10^–3^ S cm^–1^) and Li_6.6_Si_0.6_Sb_0.4_S_5_I (14.8 × 10^–3^ S cm^–1^).^5^ However, these highly conducting argyrodites are based on sulfides which exhibit poor stability in air and are not stable toward lithium metal.^6−8^

Most argyrodites with the general formula,  (L = Ag^+^, Cu^+^, Cd^2+^, etc.; A = Ga^3+^, Si^4+^, Ge^4+^, P^5+^, etc.; X = S^2–^, Se^2–^, Te^2–^; Y = Cl^–^, Br^–^, and I^–^),^9^ adopt high-temperature cubic *F*4̅3*m* and low-temperature pseudo-cubic polymorphs. The *F*4̅3*m* phases often exhibit dynamic disorder of mobile cations, which gives rise to high ionic mobility, while the low-temperature phases are stabilized by ordering of L cations onto a subset of favorable fully occupied positions. This affords lower symmetry (*P*2_1_3, *Cc*, *Pna*2_1_) structures that can be related to the *F*4̅3*m* phase through group–subgroup relationships.^10,11^

**Figure 1:**
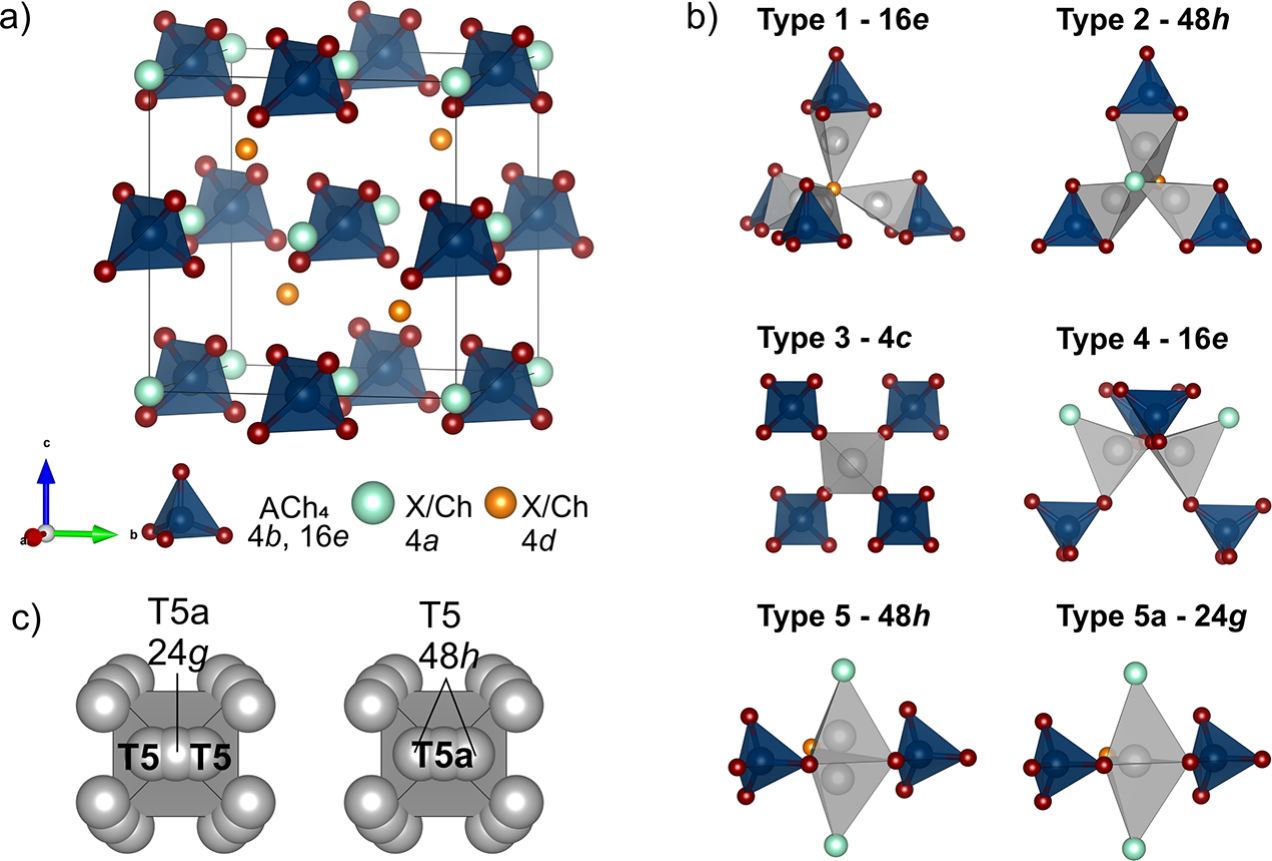
(a) Unit cell of Li_6_ACh_5_X (A = P, Si, and Al; Ch = O, S, and Se; X = Cl, Br, and I); Ch/X anions are tetrahedrally close packed on Wyckoff site (4a, 4d, and 16e) forming 136 tetrahedral voids; four are occupied by A cations on the 4b site defining ACh_4_ tetrahedra (Ch: 16e). Lithium atoms are not shown. (b) Panels showing five types (T1, T2, T3, T4, and T5) of possible interstitial tetrahedral sites available for lithium occupancy and the trigonal bipyramidal T5a site (classification proposed in ref (14)). Lithium atoms are shown in gray. (c) Octahedral Li^+^-ion cages consisting of T5 and T5a sites surrounding central anions on the 4d site; due to the close proximity of T5 and T5a sites, the total occupancy over the two sites is constrained to unity. Reproduced with permission from *Chem.—Eur. J.***2010,***16* (7), 2198–2206 (ref (14).). Copyright 2010 John Wiley and Sons.

The structures of *F*4̅3*m* lithium argyrodites such as Li_7–*x*_ACh_6–*x*_X_*x*_ (A = P, Si, Sb, Sn, and Ge; Ch = S, Se, and O; X = Cl, Br, and I) can be described by a tetrahedrally close packed arrangement of chalcogenide (Ch) and/or halogen (X) atoms^9^ on Wyckoff sites 4a, 4d, and 16e analogous to the close packing observed in cubic Lave phases,^12^ for example, MgCu_2_ (Figure 1a).^13^ The anion framework forms 136 interstitial tetrahedral voids per unit cell available for occupancy by cations. Four of these are occupied by the A (P, Si, Sb, Sn, and Ge) cations on the 4b site defining^14^ ACh_4_ tetrahedra (Ch: 16e). The remaining 132 tetrahedral sites are available for Li^+^ occupancy (Figure 1a) and can be classified on the basis of the number of Ch ions that are shared with ACh_4_ tetrahedra (Figure 1b). As such, the tetrahedral sites can be split into five subsets: Type 1 (T1) sites share faces and type 2 (T2) tetrahedra share edges with ACh_4_ tetrahedra. The type 3 (T3) and type 4 (T4) tetrahedra share four and three corners with ACh_4_ tetrahedra, respectively. The 48-fold type 5 (T5) sites share two corners with ACh_4_ tetrahedra, and the 24-fold type 5a (T5a) sits at the shared face of two neighboring T5 tetrahedra in a trigonal bipyramidal environment, with a maximum possible overall occupancy of unity across these two sites due to their close proximity.^14^ The T5 and T5a sites define octahedral cages around central anions (4d) (Figure 1c) which form a 3D network throughout the structure. Full occupancy of the T5 or T5a sites can lead to ordering of Li^+^ atoms and yields lower symmetry structures compared to *F*4̅3*m*.

Most Li^+^ argyrodites have Li^+^ occupancy of the T5 (48h) and/or T5a (24g) sites, for example, in Li_6_PS_5_I, Li^+^ atoms occupy the T5a and T5 sites in a disordered manner.^15^ Recently, the partial occupancy of other (T2, T3, or T4) lithium sites was revealed in several sulfide argyrodite materials, and this disordered distribution of Li^+^ leads to improvements in ionic conductivities. For example, partially replacing P^5+^ with Ge^4+^ in Li_6+*x*_P_1–*x*_Ge_*x*_S_5_I leads to the partial occupancy of interstitial T2 and T4 sites, providing shorter and more favorable pathways for Li^+^ diffusion and resulting in an enhancement of ionic conductivity from ∼10^–6^ to ∼10^–2^ S cm^–1^.^16^ Combined occupancy of the T2 and T3 sites was reported for Li_6+*x*_Sb_1–*x*_Sn_*x*_S_5_I,^5^ and partial occupancy of the T4 sites was observed in Li_6.15_Al_0.15_Si_0.1.35_S_5.4_O_0.6_.^17^ Detailed neutron diffraction studies have recently revealed that the Li^+^ sublattice in stoichiometric Li_6_PS_5_X (X = Cl or Br) is more complex than originally characterized and that Li^+^ ions occupy T2 sites alongside the T5 and T5a sites highlighting the importance of a highly delocalized distribution of Li^+^ formed from partially occupied interstitial sites to achieve high ionic conductivity in Li^+^ sulfide argyrodites.^18^

The ionic conductivity of sulfide argyrodites can be further controlled by tuning the anion site disorder. Mixing of sulfide and halide anions on the 4a and 4d sites increases the measured ionic conductivity of many sulfide argyrodites, for example, the ionic conductivity increases 4-fold in Li_6_PS_5_Br by tuning the synthesis conditions to increase site disorder across 4a and 4d sites.^19^

Significant effort has been invested into improving and understanding sulfide argyrodites; however, oxide argyrodites remain almost entirely unexplored, despite the propensity for oxide electrolytes to display higher stabilities to lithium metal and the potential for greater air and moisture stability.^20,21^ Only two reports of oxide argyrodite materials exist; cubic *F*4̅3*m* Li_6_PO_5_Cl and Li_6_PO_5_Br^22^ and hexagonal *P*6_3_*mc* Li_6_SiO_4_Cl_2_^23^ exhibit ionic conductivities below useable levels at room temperature (∼10^–9^ S cm^–1^). All three materials have ordered structures with fully occupied Li^+^ sites at room temperature which results in these low ionic conductivities.

We establish the first Li-rich (>6 Li atoms per formula unit) oxide argyrodite, Li_7_SiO_5_Cl, and explore the aliovalent cation substitutional chemistry by replacing Si^4+^ with P^5+^ to afford a unique Li^+^ distribution disordered across T5, T5a, T3, and T4 sites in Li_6+*x*_P_1–*x*_Si_*x*_O_5_Cl. Single-crystal X-ray diffraction shows that Li_7_SiO_5_Cl crystallizes in an ordered cubic *P*2_1_3 argyrodite structure at room temperature and exhibits an order–disorder phase transition to *F*4̅3*m* at 473 K, supported by differential scanning calorimetry (DSC). Partial occupancy of T3 and T4 Li^+^ sites in the disordered *F*4̅3*m* structures of Li_6+*x*_P_1–*x*_Si_*x*_O_5_Cl is confirmed through Rietveld analysis of high-quality synchrotron X-ray diffraction data and leads to an increase in the ionic conductivity of 3 orders of magnitude compared to existing oxide argyrodite materials measured by AC impedance and ^7^Li nuclear magnetic resonance (NMR), with a maximum ionic conductivity of 1.82(1) × 10^–6^ S cm^–1^ in *x* = 0.75. The material is stable against Li metal and exhibits greatly improved air stability compared to sulfide analogues, facilitating ease of handling.

## 2 Experimental Methods

### 2.1 Synthesis

#### 2.1.1 Materials

Li_2_CO_3_ (99.99%), SiO_2_ (silica gel, >99.0%), Li_3_PO_4_ (>99.0%), and LiCl (>99.0%) were purchased from Sigma-Aldrich. Li_2_O (>99.0%) was purchased from Alfa Aesar.

#### 2.1.2 Synthesis of Li_4_SiO_4_^24^

Precursors were dried overnight at 473 K before use. Li_2_CO_3_ (1.2331 g) and SiO_2_ (0.5013 g) were weighed according to the stoichiometric 2:1 ratio. The powders were ground in an agate mortar for 15 min, placed into an alumina crucible, and heated in air to 1073 K and annealed for 12 h before cooling to room temperature (heating and cooling rate: 5 K min^–1^). The product was ground in an agate mortar to give a white powder, which was characterized by powder X-ray diffraction (PXRD) and then used as a precursor in the synthesis of Li_6+*x*_P_1–*x*_Si_*x*_O_5_Cl.

#### 2.1.3 Synthesis of Li_6+*x*_P_1–*x*_Si_*x*_O_5_Cl

Li_4_SiO_4_, Li_3_PO_4_, Li_2_O, and LiCl were dried under dynamic vacuum (<10^–4^ mbar) at 473 K (Li_4_SiO_4_, LiCl), 673 K (Li_3_PO_4_), and 1223 K (Li_2_O) overnight before placing them inside an Ar-filled dry box (O_2_ < 0.1 ppm, H_2_O < 0.1 ppm). Fourier transform infrared (FTIR) spectroscopy was used to confirm the absence of water in each of the precursor materials. Treating the reagents in this way was crucial to achieve phase pure synthesis of Li_6+*x*_P_1–*x*_Si_*x*_O_5_Cl as the presence of water led to the formation of Li_2_OHCl alongside other impurities, even in small amounts. As such, all precursors and resulting powders were handled in an Ar-filled dry box (O_2_ < 0.1 ppm, H_2_O < 0.1 ppm). Li_4_SiO_4_, Li_3_PO_4_, Li_2_O, and LiCl were mixed in the stoichiometric ratios (Table S1) using ball milling under an Ar atmosphere. The precursors were ball milled in 1 g batches for a total time of 6 h (intervals: 20 min on, 10 min off) in 45 mL zirconia jars using seven zirconia balls (diameter: 10 mm). The resulting powder was then pressed into 5 mm diameter pellets using 300 MPa pressure. The pellets were placed into alumina crucibles, and the crucibles were placed in flame-dried quartz tubes which were sealed under vacuum (∼10^–5^ mbar). The tubes were heated to 823 K for *x* = 0.1, 0.3, 0.5, 0.6, 0.7, 0.75, 0.8, 0.85, and 0.9, held at the reaction temperature for 3 h and cooled at a rate of 5 K min^–1^. For *x* = 1, the material was heated to 873 K (ramp rate of 5 K min ^–1^), held for 6 h, and cooled at a rate of 5 K min^–1^. A range of reaction temperatures were explored, and it was found that values of *x* = 0.1–0.85 formed at the lower reaction temperature of 823 K, while for *x* = 1, it had to be heated to 873 K to form as reactions at lower temperatures lead to a mixture of starting materials (Li_4_SiO_4_ and LiCl) and Li_6_SiO_4_Cl_2_. A single crystal was picked from the *x* = 1 powder synthesized at 873 K and used for analysis. The quartz tubes were opened inside the Ar dry box, and the powders were ground in an agate mortar for further characterization.

### 2.2 PXRD

Routine assessment of sample purity was carried out using a Bruker D8 Discover diffractometer with monochromatic Cu radiation (Kα_1_, λ = 1.54056 Å) in Debye–Scherrer transmission geometry with sample powders loaded into 0.7 mm diameter borosilicate glass capillaries.

Synchrotron X-ray diffraction (SXRD) was performed at Diamond Light Source U.K. on high-resolution beamline I11^25^ at λ = 0.826552 Å for samples *x* = 0.1, 0.3, 0.5, 0.6, 0.7, 0.75, 0.8, 0.85, and 0.9. The patterns were recorded in the transmission mode [0° < 2θ < 150°] using a multianalyzer crystal detector for *x* = 0.6, 0.7, and 0.8 and a position sensitive detector (PSD, λ = 0.82660 Å) for *x* = 0.1, 0.3, 0.5, 0.75, 0.85, and 0.9. The experiments were carried out at room temperature on samples introduced into 1 mm diameter borosilicate glass capillaries.

Synchrotron variable temperature X-ray diffraction (VT-XRD) was performed in the transmission mode using a PSD (λ = 0.82660 Å) on a powder sample of Li_7_SiO_5_Cl (*x* = 1) that was introduced into a 1 mm diameter silica capillary. The experiment was performed in the temperature range 298–873 K in 25 K steps by heating and then cooled directly to room temperature to assess reversibility. To identify the phases present, databases (ICSD,^26^ PCD^27^) were searched for known materials containing Li, Si, P, O, and Cl alongside possible contaminants originating from the synthesis procedure, such as Al, Zr, C, and H.

Rietveld refinements were carried out using TOPAS Academic.^28^ Initially, Pawley fits were performed on SXRD data, refining the lattice parameters and the background using a Chebyshev function with 12 parameters. Refined parameters from final Pawley fits were then used as starting points for Rietveld refinements where the following parameters were refined: (1) scale factor, (2) atomic coordinates, (3) isotropic (Li, Si, P, O, and Cl) displacement parameters, and (4) atomic occupancies. The occupancies of Cl and O were refined, while the occupancy of the mixed Si and P site was set to the values obtained from compositional analysis (Table S6). Li^+^ sites were identified in the Fourier difference map (FDM) through the presence of positive electron density, introduced into the model and refined (atomic coordinates, atomic occupancies, and isotropic displacement parameters). The overall Li^+^ content was fixed to charge balance the refined anion content.

### 2.3 Single-Crystal X-ray Diffraction

Colorless, block-shaped crystals of Li_7_SiO_5_Cl suitable for structure determination were selected under a polarizing microscope and then mounted on a Rigaku MicroMax-007 HF X-ray generator equipped with a Mo-Kα rotating-anode microfocus source and a Saturn 724+ detector. Li_7_SiO_5_Cl was also studied by single-crystal XRD on beamline I19 at Diamond Light Source U.K. using silicon double-crystal monochromated synchrotron radiation (λ = 0.6889 Å, Pilatus 2M detector). To analyze structural phase transitions, both the Rigaku data and the synchrotron data were collected at three different temperatures, that is, 100, 300, and 500 K. Data reduction was carried out with the CrysAlis^Pro^ Version 171.40_64.53 software.^29^ All structures were solved using the intrinsic phasing method provided by the ShelXT^30^ structure solution program and refined with anisotropic displacement parameters for all atoms by least-squares minimization with the ShelXL^31^ refinement package interfaced through Olex2.^32^ The structures were checked for additional symmetry elements using the *Addsym* subroutine of PLATON.^33^

### 2.4 NMR Spectroscopy

^6^Li, ^29^Si, and ^31^P magic angle spinning (MAS) NMR experiments were performed on a 9.4 T Bruker Avance III HD spectrometer using a 4 mm HXY MAS probe (in double resonance mode) with the X channel tuned to ^6^Li at ω_0_/2π(^6^Li) = 59 MHz, ^29^Si at ω_0_/2π(^29^Si) = 79.5 MHz, and ^31^P at ω_0_/2π(^31^P) = 162 MHz. One-pulse experiments with 90° flip angle of durations 3.6 μs at a radio frequency (rf) field amplitude of ω_1_/2π(^6^Li) = 70 kHz, 5 μs at an rf amplitude of ω_1_/2π(^29^Si) = 50 kHz, and 3.8 μs at an rf amplitude of ω_1_/2π(^31^P) = 65 kHz were used. The MAS frequency ω_r_/2π was set to 10 kHz for all experiments, and quantitative recycle delays of more than 5 times the corresponding spin–lattice relaxation times *T*_1_ were used. *T*_1_ times were measured through the saturation recovery pulse sequence and the data fit to the expression 1 – exp[−(τ/*T*_1_)^α^] where τ is a variable delay and α is a stretch exponential (ranging from 0.88 to 1). All samples were packed into 4 mm zirconia rotors under an inert Ar atmosphere in a dry box (O_2_ < 0.1 ppm O_2_, H_2_O < 0.1 ppm).

Static ^7^Li VT-NMR experiments were recorded on a 9.4 T Bruker Avance III HD spectrometer equipped with a 4 mm HX high-temperature MAS probe with the X channel tuned to ^7^Li at ω_0_/2π(^7^Li) = 156 MHz. All ^7^Li NMR spectra were obtained with a hard 90° flip angle of duration determined by the nutation frequency of the respective sample (values ranged from 2 to 3 μs) at an rf field amplitude of ω_1_/2π(^7^Li) = 83 kHz at each temperature under quantitative recycle delays of more than 5 times the value of *T*_1_. The ^7^Li *T*_1_ values were also measured through the saturation recovery pulse sequence, and the same expression was used to fit the data. The α values for ^7^Li VT experiments ranged from 0.8 to 1 and were used in order to take into account the expected distribution of correlation times and temperature gradients across the sample. All samples were flame-sealed in Pyrex inserts under vacuum (O_2_ < 0.1 ppm, H_2_O < 0.1 ppm). The temperature calibration of the 4 mm HX high-temperature MAS probe was performed with the chemical shift thermometer Pb(NO_3_)_2_ using ^207^Pb NMR,^34^ and the phase transitions of CuI and CuBr were measured using ^63^Cu NMR.^35,36^ The errors associated with this method were calculated using the line broadening of the isotropic peak and are in the 5–15 K range.

The ^6^Li and ^7^Li shifts were referenced to 10 M LiCl in D_2_O at 0 ppm, while ^29^Si chemical shifts were externally referenced to the lowest-frequency signal of octakis(trimethylsiloxy)silsesquioxane at −109 ppm (relative to tetramethylsilane primary reference at 0.0 ppm),^37^ and ^31^P chemical shifts were referenced to 85% H_3_PO_4_ at 0 ppm.

### 2.5 DSC

Heat flux profiles were measured on Li_7_SiO_5_Cl from 16.3 mg of powdered sample in a 40 μL aluminum crucible cold-welded under an Ar atmosphere (O_2_ < 0.1 ppm, H_2_O < 0.1 ppm). Data were collected from 298 to 523 K at heating and cooling rates of 10 K min^–1^ under a flow of helium (50 mL min^–1^) using a Netzsch DSC 404 F1 differential calorimeter.

### 2.6 Densification by Spark Plasma Sintering

Dense pellets of Li_6+*x*_P_1–*x*_Si_*x*_O_5_Cl (*x* = 0.7, 0.75, and 0.8) were prepared via spark plasma sintering (SPS) using a commercial Thermal Technology LLC DCS10 furnace. For each pellet, ∼0.3 g of powder was loaded into a 10 mm inner diameter tungsten carbide die set (WC with 6% Co binder) lined with graphite foil inside an Ar-filled dry box (O_2_ < 0.1 ppm, H_2_O < 0.1 ppm). Vacuum grease was applied to the punches to form a temporary air-tight seal as the die was transferred into the SPS furnace chamber which was then evacuated to <5 × 10^–2^ mbar. 800 MPa of uniaxial pressure was applied to the powders at a rate of 100 MPa min^–1^. Samples were heated to 728 K at a rate of 50 K min^–1^ and annealed for 30 s, before the applied current was turned off and the die set allowed to cool to room temperature and the pressure released at a rate of 100 MPa min^–1^. The temperature was monitored through a borehole in the side of the die via a pyrometer. The temperature of the sample would typically overshoot the target temperature by 10–15 K during this procedure. Once at room temperature, the die set was transferred to the dry box, the pellets were removed, and the graphite foil on the pellet surface was removed by lightly polishing with SiC polishing paper. This procedure resulted in pellets with densities of 94–96% relative to the theoretical densities of Li_6+*x*_P_1–*x*_Si_*x*_O_5_Cl (*x* = 0.7, 0.75, and 0.8).

### 2.7 AC Impedance Spectroscopy and DC Polarization

Pellets of Li_6+*x*_P_1–*x*_Si_*x*_O_5_Cl (*x* = 0.1, 0.3, 0.5, 0.6, 0.7, 0.8, 0.85, 0.9) were prepared by uniaxially pressing ∼30 mg of starting material in a 5 mm cylindrical steel dye at a pressure of 300 MPa. The pellets were annealed in an evacuated, flame-dried quartz tube for 3 h at 873 K. Using this method, a relative density of 74–85% was achieved. In addition, pellets of Li_6+*x*_P_1–*x*_Si_*x*_O_5_Cl (*x* = 0.7, 0.75, and 0.8) were prepared by SPS, and with this method, relative densities of 94–96% were achieved.

Alternating current (AC) impedance measurements were conducted using an impedance analyzer (Keysight impedance analyzer E4990A). A sputtered gold coating of ∼300 nm thickness was used as the ion-blocking electrodes. Sputtering was achieved in a glovebox using the sputter coater Q150R using a pressure of 10^–1^ mbar and a sputtering current of 60 mA. Temperature-dependent conductivity measurements were performed under a dry argon atmosphere in the frequency range of 2 MHz to 20 Hz (with an amplitude of 100 mV). Measurements were performed at room temperature and in the temperature range 323–423 K in 25 K steps. ZView2^38^ program was used to fit the impedance spectra with an equivalent circuit.

Direct current (DC) polarization data were collected on Au|Li_6+*x*_P_1–*x*_Si_*x*_O_5_Cl|Au (*x* = 0.7, 0.75, and 0.8) symmetric cells at 298 K. Constant voltages of 0.04–1 V were applied for 2 h, and the current variation with time was recorded. The DC polarization curves were recorded until a steady-state current was obtained at any applied voltage. Data were collected from samples prepared via reactive sintering and via SPS.

### 2.8 Electrochemical Li Plating/Stripping

A pellet of Li_6.75_P_0.25_Si_0.75_O_5_Cl was synthesized via SPS. Symmetric Li|Li_6.75_P_0.25_Si_0.75_O_5_Cl|Li cells were assembled inside an Ar-filled glovebox (O_2_ ≤ 0.1 ppm, H_2_O ≤ 0.1 ppm). The thickness of the Li_6.75_P_0.25_Si_0.75_O_5_Cl pellet was ∼0.9 mm. Two discs of Li metal (99.9%, 0.38 mm thickness, Sigma-Aldrich) were pressed onto steel discs. The steel/Li|Li_6.75_P_0.25_Si_0.75_O_5_Cl|Li/steel stack was carefully aligned, compressed, and sealed inside a two-electrode coin cell. The Li_6.75_P_0.25_Si_0.75_O_5_Cl|Li interface stability was evaluated by galvanostatic Li plating/stripping tests, which were performed at 298 K at a current density of 20 μA cm^–2^ (20 min per half-cycle) using a BioLogic VSP 300 potentiostat.

### 2.9 SEM

Scanning electron microscopy (SEM) images and energy-dispersive X-ray (EDX) spectroscopy data were obtained on a Hitachi S-4800 microscope. Imaging was performed at an acceleration voltage of 10 kV and a current of 10 μA. Pellets were attached to an aluminum stub using a carbon tape and Cr-coated to reduce charging effects. EDX spectroscopy was performed at a voltage of 20 kV and a current of 20 μA using a detector from Oxford Instruments (x-act). For SEM–EDX, powders were spread on a carbon tape attached to an aluminum stub. Ten measurements were collected for each composition using Aztec software. EDX data were corrected by measuring standards for each element.

## 3 Results and Discussion

### 3.1 Li_7_SiO_5_Cl: Synthesis, Thermal Behavior, and Crystal Structure

#### 3.1.1 Synthesis of Li_7_SiO_5_Cl

By fully replacing P^5+^ in Li_6_PO_5_Cl with Si^4+^, the composition Li_7_SiO_5_Cl was targeted experimentally under a range of reaction conditions (723–923 K for 6–72 h). At 873 K and an annealing time of 6 h, peaks were observed in the laboratory PXRD pattern that could not be indexed to any known phases in the Li–Si–O–Cl phase field (Al, Zr, C, and H were also considered as possible contaminants). It proved crucial to dry the starting materials under dynamic vacuum before synthesis (cf. Section 2.1.3: Synthesis of Li_6+*x*_P_1–*x*_Si_*x*_O_5_Cl) as Li_2_(OH)Cl and other impurities formed in large quantities if the starting materials were not dried completely. The use of completely dried reagents yielded the highest purity sample of 83.64% across the range of sampled synthesis conditions compared to ∼60% phase purity when incompletely dried reagents were used (Figure S1). A range of reaction conditions were explored to ensure the reaction had reached equilibrium. Annealing times below 6 h lead to the presence of starting materials (Li_4_SiO_4_ and LiCl), indicating that the reaction had not been completed, while annealing times above 6 h lead to larger amounts of decomposition products (Li_8_SiO_6_ and Li_6_SiO_4_Cl) (Figure S2). Synthesis at 873 K for 6 h yielded colorless block-shaped crystals which were selected for diffraction measurements, and high-resolution synchrotron single-crystal diffraction data were collected at 100, 300, and 500 K.

#### 3.1.2 Structure Determination of Li_7_SiO_5_Cl

The structures of Li_7_SiO_5_Cl at 100, 300, and 500 K were solved from high-resolution single crystal X-ray diffraction data collected at beamline I19 at Diamond Light Source. All investigated crystals showed twinned agglomerates at 100 and 300 K, which were solved in the cubic space group *P*2_1_3 and refined applying a twin law 1̅00, 001, 010 with domain fractions of approximately 1:1. The 500 K structure was solved in the higher symmetry space group *F*4̅3*m*, indicating the presence of a phase transition between 300 and 500 K. The final FDMs did not indicate any additional atomic sites. There are no systematic deviations from the least-squares line for the observed and calculated structure factors of all final models. The final anisotropic structure refinement converged at *R*_1_/w*R*_2_ = 0.0238/0.0605 (*I* ≥ 2σ (*I*)) for 100 K, *R*_1_/w*R*_2_ = 0.0229/0.0603 (*I* ≥ 2σ (*I*)) for 300 K, and *R*_1_/w*R*_2_ = 0.0422/0.1017 (*I* ≥ 2σ (*I*)) for 500 K.^39−41^ The twin domain ratios were established to be equal to 0.653(3):0.347(3) for the 100 K model and 0.655(3):0.345(3) for the 300 K model. Lattice parameters were 8.25320(10), 8.27250(10), and 8.3032(2) Å at 100, 300, and 500 K, respectively. Crystallographic data for the structures at 100, 300, and 500 K are available in the Supporting Information (Tables S2–S5).

High-resolution synchrotron PXRD data were collected on the highest purity powder sample (83.64% purity) of Li_7_SiO_5_Cl from 298 to 898 K in 25 K steps. Peaks that could not be assigned to previously reported phases in the Li–Si–O–Cl phase field were assigned to *P*2_1_3 symmetry (*h*00: *h* = 2*n*) adopted by Li_7_SiO_5_Cl. A Pawley fit (Figure S3b) to the 300 K dataset gave lattice parameters of *a* = 8.284296(5) Å, consistent with that from single-crystal diffraction measurement, with Li_4_SiO_4_ (8.65%), Li_6_SiO_4_Cl_2_ (4.50%), Li_8_SiO_6_ (0.78%), and LiCl (2.43%) present as impurity phases. At ∼473 K, the disappearance of small peaks associated with the *P*2_1_3 symmetry indicated a phase transition to a higher symmetry phase (Figure S3d). The systematic absences in patterns recorded above 473 K were consistent with the higher symmetry *F*4̅3*m* space group (*hkl*: *h* + *k*, *h* + *l*, *k* + *l* = 2*n*, 0*kl*: *k, l* = 2*n*, *hhl*: *h* + *l* = 2*n*, *h*00: *h* = 2*n*). A plot of lattice parameters versus temperature shows a discontinuous change in slope at 473 K (Figure S4a) associated with this phase transition. This reversible phase transition from *P*2_1_3 to *F*4̅3*m* symmetry is also observed through DSC carried out on Li_7_SiO_5_Cl powder which shows reversible endothermic and exothermic events at 472.1(6) K on heating and cooling (Figure S4b).

#### 3.1.3 Structure Description of Li_7_SiO_5_Cl at 100, 300, and 500 K

**Figure 2:**
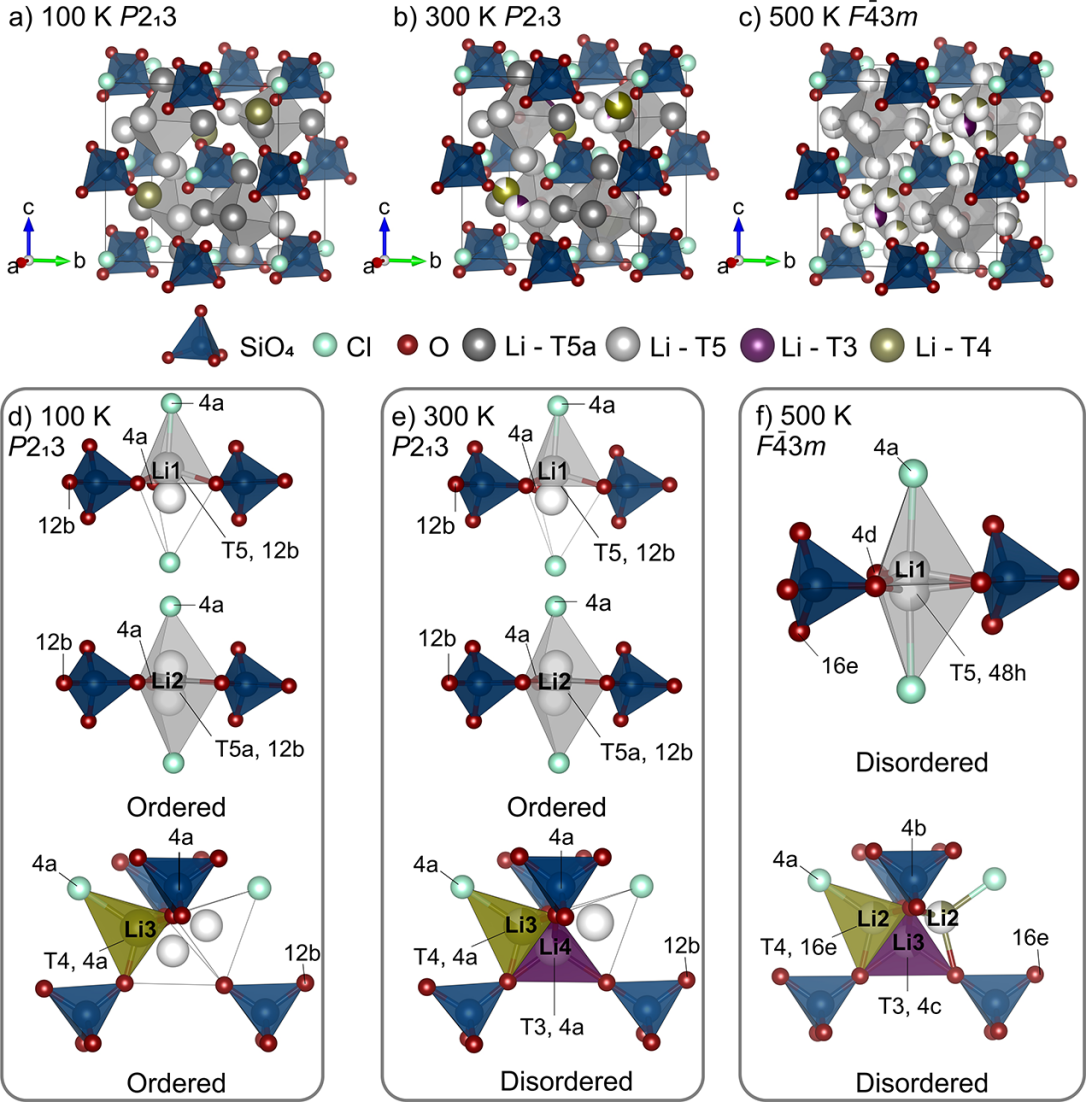
Crystal structure of Li_7_SiO_5_Cl at 100, 300, and 500 K. Atom and polyhedra colors: SiO_4_ tetrahedra (dark blue), O (red), Cl (light blue), Li—T5a site (dark gray), Li—T5 site (gray), Li—T3 site (purple), Li—T4 site (green), and vacant sites (white). Unit cell at (a) 100, (b) 300, and (c) 500 K; lithium ion environments at (d) 100 (e) 300, and (f) 500 K; panels (d–f) highlight how the Li sublattice transitions form a fully ordered arrangement at 100 K (*P*2_1_3), with disorder around the T4 and T3 sites at 300 K (*P*2_1_3), to a fully Li site disordered structure at 500 K (*F*4̅3*m*). To facilitate comparison, sites which are partially occupied in the higher symmetry 500 K structure are drawn as vacant sites as white spheres in the (d) 100, (e) 300 K structures. For (d–f), two out of the four possible T4 sites around the T3 site are omitted for clarity.

The unit cell of Li_7_SiO_5_Cl obtained from single-crystal diffraction at 100 K contains one Cl site (4a), three O sites [4a (O1, O2) and 12b (O3)], one Si site (4a) (as determined by ^29^Si MAS NMR spectrum, which displays a single narrow signal at −65 ppm corresponding to a SiO_4_^4–^ unit, Figure S5), and three fully ordered Li^+^ sites [12b (Li1, Li2) and 4a (Li3)] as shown in Figure 2a. The structure at 100 K (space group: *P*2_1_3) is an ordered version of the high-symmetry *F*4̅3*m* structure adopted by many cubic lithium argyrodites. Li1 and Li2 fully occupy T5 (12b) and T5a (12b) sites in tetrahedral LiO_3_Cl and trigonal bipyramidal LiO_3_Cl_2_ environments, respectively. Vacant sites corresponding to T5 sites in the disordered high symmetry 500 K structure (*F*4̅3*m*) are drawn as white spheres in Figure 2d–f to facilitate comparison. The Li3 atoms fully occupy T4 (4a) sites in tetrahedral LiO3Cl environments. Vacant T4 (12b) and T3 (4c) sites, which are partially occupied at higher temperatures, are drawn as white spheres (Figure 2d). The full occupancy of the T4 (4a) site by Li2 causes a displacement of Li1 away from the central trigonal bipyramidal T5a onto one of the proximal tetrahedral T5 sites. In comparison, the T4 sites are unoccupied in Li_6_PO_5_Cl resulting in the full occupancy of trigonal bipyramidal T5a sites only. Li_7_SiO_5_Cl represents the first Li^+^ argyrodite material with *P*2_1_3 symmetry, which is adopted by some Ag and Cu argyrodites such as Ag_7_PS_6_ and Cu_7_PS_6_. In these argyrodites, the mobile cations, Ag and Cu, fully occupy a combination of tetrahedral (T5), trigonal bipyramidal (T5a), and linear environments,^9^ whereas the T5a, T5, and tetrahedral T4 sites are occupied by Li^+^ in Li_7_SiO_5_Cl.

At 300 K, the *P*2_1_3 symmetry is retained (Figure 2b), but the distribution of occupied Li^+^ sites changes significantly. The lithium atoms occupying the T5 and T5a sites (Li1 and Li2) remain unchanged, but Li^+^ atoms which fully occupy the T4 site at 100 K partially occupy T4 (4a) and proximal T3 (4c) sites at 300 K, introducing Li^+^ site disorder into the material (Figure 2e). This result is supported through the room-temperature ^6^Li MAS NMR of Li_7_SiO_5_Cl (Figure S6). The spectrum displays an intense resonance at 1.1 ppm assigned to Li^+^ atoms occupying the T5 and T5a sites (Li1, Li2) as well as a smaller second resonance at 0.6 ppm integrating 18% of the main peak. This second resonance corresponds to Li^+^ atoms partially occupying the T4 and T3 sites (Li3 and Li4), and this is further evidenced by the increased linewidth of this resonance compared with the main peak (65 vs 35 Hz) attributed to inhomogeneous broadening arising from the local site disorder at the T4 and T3 sites. While the combined occupancy of T2 and T3 or T2 and T4 sites has been observed in sulfide argyrodites,^5,16,17^ this is the first time the combined occupancy of T3 and T4 sites alongside T5 and T5a sites has been observed in Li^+^ argyrodites and is generated by oxide anions.

Above 472.1(6) K, Li_7_SiO_5_Cl undergoes a reversible phase transition to the higher-symmetry *F*4̅3*m* space group as confirmed by the change in lattice parameters, disappearance of superlattice reflections, and exo- and endothermic peaks observed via DSC (Figures S3d and S4). The structure solved at 500 K contains one Cl site (4a), 2 O sites [16e (O1) and 4d (O2)], one Si site (4b), and three Li^+^ sites [48h (Li1), 16e (Li2), and 4c (Li3)] and is shown in Figure 2c. The Li^+^ atoms which are ordered below 473 K on the tetrahedral T5 and trigonal bipyramidal T5a sites partially occupy T5 (Li1, 48h, site occupancy factor (*s.o.f.*) 0.490) sites above 473 K with the T5a site unoccupied. This change in symmetry and the corresponding increase in the number of equivalent sites introduce further Li^+^ disorder into the material (Figure 2f). The Li^+^ atoms on the T3 [4a, *s.o.f.*: 0.160(1)] and one 4a T4 [*s.o.f.*: 0.840(15)] sites at 300 K are now disordered over the T3 site [Li3, 4c, *s.o.f.*: 0.4434(12)] and the symmetry-equivalent 16e T4 sites [Li2, *s.o.f.:* 0.1886(3)]. The T3 site occupancy increases from 0.160(1) to 0.4434(12) when the temperature is increased, and the symmetry is changed alongside a decrease in T4 site occupancy from 0.840(15) (4a) to 0.1886(3) (16e), as shown in Figure 2f.

Li^+^ ions form octahedral cages around O^2–^ anions on the 4a (*P*2_1_3) and 4d (*F*4̅3*m*) sites. These cages are shown as gray octahedra in Figure 2a–c. In the 300 K *P*2_1_3 structure of Li_7_SiO_5_Cl, the octahedral cages are formed by Li1 and Li2 atoms, and the intracage distances between neighboring T5 and T5a lithium sites are ∼2.65 Å [Li1–Li1: 2.700(7) Å, Li1–Li2: 2.532(7) Å, Li2–Li2: 2.718(9) Å]. The additional lithium sites in Li_7_SiO_5_Cl (T4 and T3) introduce new Li–Li distances that are not accessible in Li_6_PO_5_Cl (in which T4 and T3 sites are unoccupied). The shortest intercage distances in Li_7_SiO_5_Cl are 2.185(5) Å (T5a–T3) and 2.229(6) Å (T5–T4) compared to 3.08(9) Å in Li_6_PO_5_Cl (T5a–T5a), in which Li^+^ distances are maximized as Li^+^ atoms occupy T5a sites only (Figure S7).

In the 500 K *F*4̅3*m* structure, both the intracage and intercage distances are reduced as Li^+^ atoms partially occupy all T5 sites. The intracage distances (T5–T5) reduce from ∼2.65 to 2.45(6) Å, and the shortest intercage distances (T5–T4) reduce from 2.229(5) to 1.66(7) Å. In contrast, the T3–T4 distances are increased from 1.251(17) to 1.40(6) Å compared to the *P*2_1_3 (300 K) structure (Figure S7).

### 3.2 Solid Solution: Synthesis and Structure

#### 3.2.1 Synthesis of Li_6+*x*_P_1–*x*_Si_*x*_O_5_Cl

A solid solution between Li_6_PO_5_Cl and Li_7_SiO_5_Cl was investigated with the aim of introducing excess Li^+^ and disordered partially occupied Li^+^ sites in Li_6+*x*_P_1–*x*_Si_*x*_O_5_Cl, analogous to those observed in the 500 K structure of Li_7_SiO_5_Cl. Values of *x* = 0.1, 0.3, 0.5, 0.6, 0.7, 0.75, 0.8, 0.85, and 0.9 were targeted through solid-state reactions. The starting materials were dried thoroughly and ball milled in the stoichiometric ratios for 6 h before annealing in evacuated quartz ampoules under optimized conditions at 823 K for 3 h. It was crucial to dry and confirm the absence of water in the reagents through FTIR spectroscopy as the presence of even small amounts of water gave products of significantly lower purity with impurities of Li_2_(OH)Cl, Li_4_SiO_4_, and Li_3_PO_4_ (Figure S8). Ball milling the reagent mixtures ensured adequate mixing before heat treatment and was necessary to achieve single-phase argyrodites at these compositions (Figure S8).

All values of *x* crystallize in a cubic space group (*P*2_1_3 or *F*4̅3*m*), and a peak shift to lower 2θ values with increasing *x* confirms the successful incorporation of Si (*r*_P(V)_ = 0.17 Å, *r*_Si(IV)_ = 0.26 Å)^42^ into the structure (Figure S9). It was not possible to make phase pure powders of *x* = 0.9 and 1 under the synthetic conditions tested, and noticeable amounts of Li_4_SiO_4_ and Li_6_SiO_4_Cl_2_ impurities (>10%) were present in these compositions. Compositions where *x* = 0.1–0.85 were all >97% pure with impurities of Li_4_SiO_4_, Li_3_PO_4_, Li_2_OHCl, Li_6_SiO_4_Cl_2_, LiCl, Li_2_O, LiCl, and ZrO_2_ present in small amounts in some samples. The small amounts of impurities likely originate from unwanted side reactions as well as small amounts of unreacted starting materials. ZrO_2_ is introduced through the ball-milling process which is performed using zirconia media.

#### 3.2.2 Structure Determination of Li_6+*x*_P_1–*x*_Si_*x*_O_5_Cl

**Figure 3:**
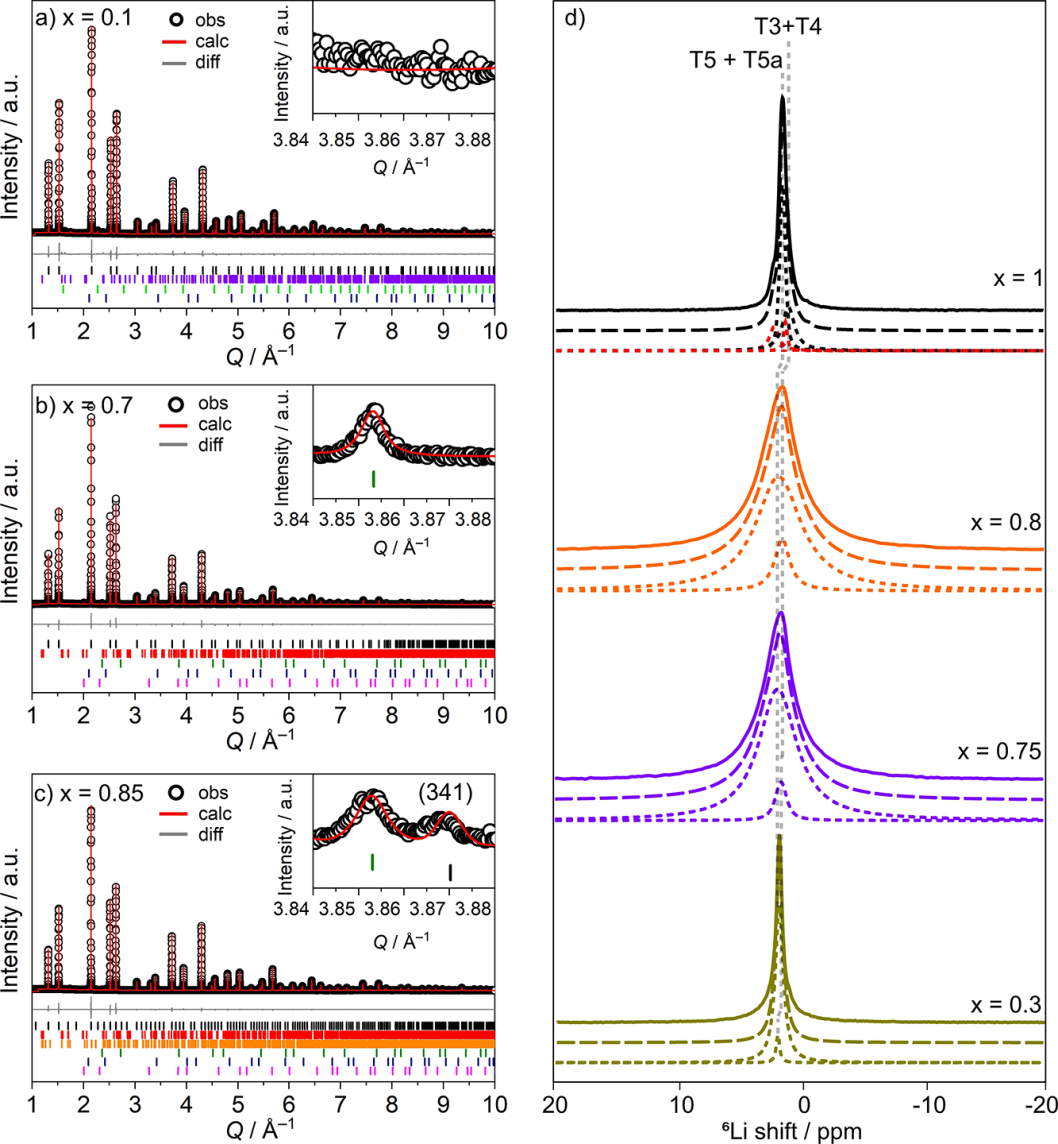
Rietveld refinement against SXRD of Li_6+*x*_P_1–*x*_Si_*x*_O_5_Cl (Diamond Light Source I11 beam line) with *I*_obs_ (black circles), *I*_calc_ (red line), *I*_obs_ – *I*_calc_ (gray line), and Bragg reflections (black tick marks for Li_6+*x*_P_1–*x*_Si_*x*_O_5_Cl, red tick marks for Li_4_SiO_4_, purple tick marks for Li_3_PO_4_, light green tick marks for Li_3_OCl, blue tick marks for LiCl, green tick marks for Li_2_O, pink tick marks for ZrO_2_, and orange tick marks for Li_6_SiO_4_Cl_2_). (a) *x* = 0.1; (b) *x* = 0.7; (c) *x* = 0.85; inset highlights (341) reflection consistent with *P*2_1_3 symmetry. (d) ^6^Li MAS NMR spectra of Li_6+*x*_P_1–*x*_Si_*x*_O_5_Cl (*x* = 0.3, 0.75, 0.8, and 1). Full width half-maximum values of 50, 130, 150, and 35 Hz were observed for *x* = 0.3, 0.75, 0.8, and 1, respectively. The experimental spectra (full lines), total fit (dashed lines), spectral deconvolution (dotted lines) of the two Li resonances (T5 + T5a and T3 + T4), and Li_4_SiO_4_ impurity (∼10 mol %) (red dotted lines) are also shown. The vertical dashed lines highlight the slight change in shift when *x* increases to 1 (spectrum also shown in Figure S5).

The pure silicate Li_7_SiO_5_Cl adopts the *P*2_1_3 symmetry, while the previously reported Li_6_PO_5_Cl adopts the higher *F*4̅3*m* symmetry at room temperature.^22^ Careful analysis of the room-temperature synchrotron powder diffraction data for the compositions *x* = 0.1–0.75 highlight the absence of additional reflections associated with the *P*2_1_3 space group symmetry, and the systematic absences for these compositions agree with the *F*4̅3*m* space group (*hkl*: *h* + *k*, *h* + *l*, *k* + *l* = 2*n*, 0*kl*: *k*, *l* = 2*n*, *hhl*: *h* + *l* = 2*n*, *h*00: *h* = 2*n*). For *x* = 0.8 and *x* = 0.85, broad, low-intensity reflections consistent with *P*2_1_3 symmetry (*h*00: *h* = 2*n*) are visible in the SXRD data (Figure 3c, inset). Pawley fits were carried out using *F*4̅3*m* symmetry for *x* = 0.1–0.75 and *P*2_1_3 symmetry for *x* = 0.8 and 0.85 (Figure S10). The additional peaks observed for *x* = 0.8 and *x* = 0.85 indicate a degree of structural ordering and are broader than the subcell peaks, and separate pseudo-Voigt functions were used to fit the supercell and subcell reflections.

Rietveld refinements against synchrotron PXRD data were carried out for compositions with *x* = 0.1–0.85. The Si and P occupancies were fixed at values obtained through compositional analysis (ICP and SEM–EDX), and positions (Si/P, O, Cl), occupancies (O, Cl), and isotropic displacement parameters (Si/P, O, Cl) were refined first before careful refinement of the Li^+^ atoms (atomic coordinates, atomic occupancies, and isotropic displacement parameters) was carried out. Rietveld refinements revealed four Li^+^ sites that are occupied to different extents in Li_6+*x*_P_1–*x*_Si_*x*_O_5_Cl: T5, T5a, T4, and T3, analogous to those observed in Li_7_SiO_5_Cl. The overall Li^+^ content was constrained to charge balance the refined anion content. The T5 and T5a and also the T3 and T4 sites are in close proximity, and the total Li^+^ content over both sets of sites was limited to a maximum value of unity. Compositional analysis carried out via SEM–EDX and ICP confirm the increase in Li^+^ and Si contents with increasing *x* (Table S6). The Rietveld refinements for compositions with *F*4̅3*m* (*x* = 0.1–0.75) and *P*2_1_3 (*x* = 0.8, 0.85) symmetries will be discussed separately and in more detail.

*x* = 0.1–0.75*:* The 500 K *F*4̅3*m* structure of Li_7_SiO_5_Cl was used as a starting model for Rietveld refinements. The starting model contained Li^+^ atoms on the T5 (48h) position, but additional electron density centered on the T5a (24g) positions was visible in the FDM for these compositions. Therefore, the T5a site was introduced into the model and refined alongside the T5 sites, which led to an improvement in fit quality for all values of *x*, for example, addition of the T5a site to the *x* = 0.7 model improved *R*_wp_ from 7.24 to 6.87%. The remaining Li^+^ atoms were located on the T3 (4c) and T4 (16e) sites for *x* = 0.3–0.75. For *x* = 0.1, additional Li^+^ atoms occupy the T3 site only with the T4 site empty. The refined isotropic displacement parameters of the T3 position were large for *x* = 0.1, so were fixed to 4 Å^2^. For *x* > 0.1, Li^+^ atoms occupy T3 and T4 sites. These positions refine stably, and excluding them from the model led to a worse fit, for example, excluding T3 and T4 sites in *x* = 0.7 led to an increase in *R*_wp_ from 5.60 to 6.34%. Removing these sites from the model also led to positive electron density on the sites in the FDM (Figure S11). For *x* = 0.3–0.75, the refined isotropic displacement parameters of the T4 sites are relatively large compared to other Li^+^ sites in the structure (*B*_iso_: ∼4 Å^2^ for the T4 site compared to ∼1 Å^2^ for T5, T5a, and T3 sites) and indicates evidence for local Li^+^ motion leading to delocalisation of the electron density around the T4 position.

*x* = 0.8–0.85: The additional small peaks observed in *x* = 0.8 and 0.85 are consistent with *P*2_1_3 symmetry (Figure 3c, inset), indicating a degree of structural ordering for these compositions. Therefore, the *F*4̅3*m* structure of *x* = 0.7 was transformed to *P*2_1_3 and used as a starting point for Rietveld refinement for *x* = 0.8 and 0.85. The lower symmetry of *P*2_1_3 enables refinement of the anion positions which occupy special Wyckoff sites in the higher symmetry *F*4̅3*m* space group. In *x* = 0.85, refining the Si/P, Cl, O1, O2, and O3 positions and isotropic displacement parameters and Cl, O1, O2, and O3 occupancies led to an improvement in *R*_wp_ from 3.89 to 3.75% as the Cl, Si/P, and O3 anions move off the ideal high symmetry positions, that is, Cl is displaced by 0.0348(1) Å along the body diagonal in *x* = 0.85 compared to a displacement of 0.1265(5) Å in *x* = 1 (*P*2_1_3, 300 K). When the symmetry is lowered from *F*4̅3*m* to *P*2_1_3, the 48-fold T5 site splits into four 12b sites, and the 24-fold T5a site splits into two 12b sites. Refining the occupancies of these sites gave *s.o.f.* of 0.213(2), 0.291(2), 0.176(2), and 0.355(2) for the T5 sites (Li1a, Li1b, L2a, and Li2b, respectively). The T5a sites refined to *s.o.f.* of 0.487(2) and 0.470(2) (Li1 and Li2, respectively). This led to an improvement in *R*_wp_ from 3.75 to 3.70% and visually improved the fit to the low-intensity ordering peaks, consistent with partial ordering of Li^+^ over T5 sites that are no longer equivalent symmetrically.

Similarly, the 16-fold T4 sites in the *F*4̅3*m* setting (16e) are split into 12b and 4a positions in *P*2_1_3. The T3 site is 4-fold in both *F*4̅3*m* (4c) and *P*2_1_3 (4a) symmetries. Refining the occupancies of the T3 and T4 sites led to *s.o.f.* of 0.373(16) and 0.081(6) for T4 (4a) and T4 (12b) sites, respectively, and 0.240(3) for the T3 (4a) site. This onset of ordering on the T4 sites improved the *R*_wp_ from 3.70 to 3.65% and led to a visible improvement of the fit quality, particularly to the ordering peaks. The final Rietveld fits for *x* = 0.1, 0.7, and 0.85 are shown in Figure 3, and the Pawley and Rietveld fits for all values of *x* can be found in the Supporting Information (Tables S7 and S8 and Figures S10 and S12).

^6^Li, ^29^Si, and ^31^P MAS NMR spectra collected for *x* = 0.3, 0.75, 0.8, and 1 further confirm the results obtained from Rietveld analysis. A single ^29^Si resonance at approximately −65 ppm was observed and indicates a single SiO_4_^4–^ unit (Figure S5). ^31^P MAS NMR spectra are given in Figure S13, and although phosphorus atoms occupy only one site in the long-range crystal structure refined by diffraction, multiple contributions are observed in the overall ^31^P MAS NMR signal. These signals arise from the sensitivity of the ^31^P nucleus to the second coordination sphere, namely, the different T3, T4, and T5 and T5a Li^+^ environments local to the P (4b) position. The chemical shift of the main ^31^P resonance decreases with increasing *x*, highlighting the increased statistical probability of a greater number of Li^+^ atoms in the second coordination sphere of the P (4b) position, arising from the increased Li^+^ content. The observed resonances and line shapes compare well with contributions assigned from Li^+^ site occupancies obtained from Rietveld refinement (Table S8, Figure S15) and NMR resonances based on the relationship between the coordination number and the chemical shift (Table S9, Figure S16), corresponding to the different possible second nearest neighbor environments for phosphorous. These results complement and confirm the Li^+^ site occupancy factors obtained from Rietveld refinement (Figure S16 and see the Supporting Information for extended details). ^6^Li MAS NMR spectra are given in Figure 3d, and each show two resonances associated with Li_6+*x*_P_1–*x*_Si_*x*_O_5_Cl; the most intense resonance corresponds to Li^+^ atoms occupying the T5 and T5a sites and a second less intense resonance corresponding to Li^+^ atoms partially occupying the T4 and T3 sites. The resonances associated with the T4/T3 Li^+^ sites integrate at 5, 11, and 15% of the main peak for *x* = 0.3, 0.75, and 0.8, respectively, and compare well with the expected ratios from Rietveld refinement of 4, 13, and 17%. Moreover, the full width at half-maximum values of the overall resonance increase as *x* increases from 0.3 to 0.75 which is due to the increased inhomogeneous broadening associated with the increased site disorder. The peak width then decreases again as *x* increases from 0.8 to 1 as the structure becomes ordered once more.

#### 3.2.3 Structure Description

All compositions in Li_6+*x*_P_1–*x*_Si_*x*_O_5_Cl adopt a cubic argyrodite structure with tetrahedral close packing of oxide and chloride anions and Si/P and Li^+^ cations filling tetrahedral holes, analogous to Li_7_SiO_5_Cl and other *F*4̅3*m* oxide and sulfide argyrodites. The O and Cl anions are fully ordered in Li_6+*x*_P_1–*x*_Si_*x*_O_5_Cl with no mixing of O^2–^ (4d) and X^–^ (X = Cl, Br, I; 4a) anions which is often reported for S^2-^ and X^-^ anions in sulfide argyrodites. This is likely due to the large relative size differences between O^2-^ (1.42 Å) and Cl^–^ (1.81 Å) compared to S^2–^ (1.84 Å) and Cl^–^ (1.81 Å), Br^–^ (1.96 Å), and I^–^ (2.2 Å).^42^ The lithium ions fill interstitial T5, T5a, T3, and T4 sites, a Li^+^ distribution distinct from sulfide Li^+^ argyrodites.

**Figure 4:**
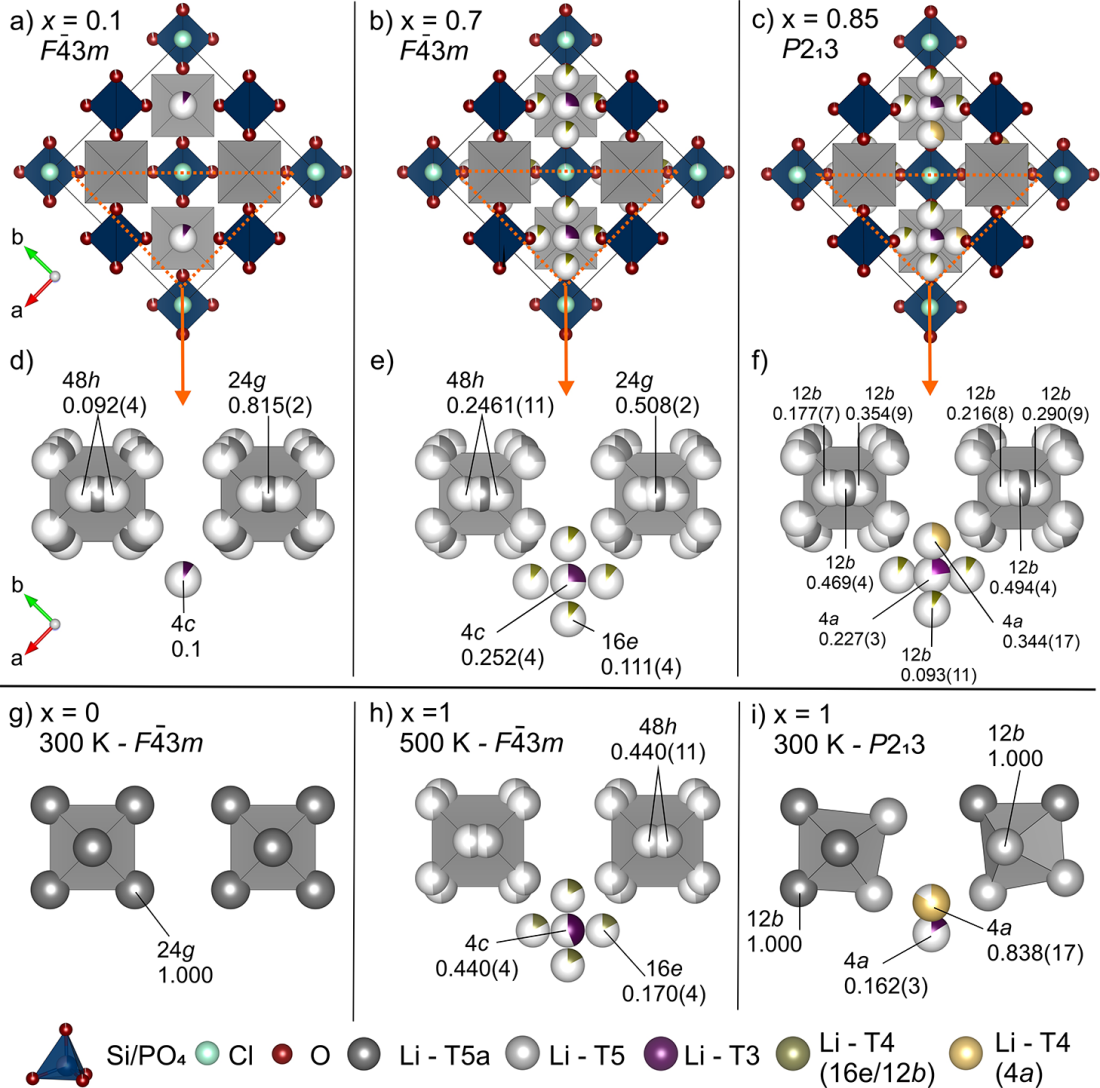
Structures of *x* = 0, 0.1, 0.7, 0.85, and 1 in Li_6+*x*_P_1–*x*_Si_*x*_O_5_Cl highlighting the different Li sites occupied and associated symmetry settings as a function of composition and temperature in Li_7_SiO_5_Cl; atom and polyhedra colors: SiO_4_ tetrahedra (dark blue), O (dark red), Cl (light blue), Li—T5a site (dark gray), Li—T5 site (light gray), Li—T3 site (purple), Li—T4 site [green (16e/12b), and yellow (4a)]. Unit cell for (a) *x* = 0.1, (b) *x* = 0.7, and (c) *x* = 0.85 (T5 and T5a Li atoms are omitted for clarity with octahedral cages shown in gray); orange triangle highlights the structural subsection shown in panels (d–f); (d–i) octahedral Li ion cages (T5 and T5a) surrounding central oxide anions and interstitial T3 and T4 sites with their *s.o.f.* for (d) *x* = 0.1, (e) *x* = 0.7, (f) *x* = 0.85, (g) *x* = 0, (h) *x* = 1, (500 K), and (i) *x* = 1 (300 K).

Li^+^ atoms in *x* = 0.1 (*F*4̅3*m*) occupy three separate sites: the T3 site and the T5 and T5a sites which form octahedral cages around central oxide anions (4d). The majority of Li^+^ atoms occupy the T5a sites with some occupancy of the T5 site giving rise to slight disorder on the octahedral cages. The remaining Li^+^ atoms occupy the T3 site (Table 1, Figure 4a,d). This is distinct from Li_6_PO_5_Cl (*x* = 0),^22^ in which Li^+^ occupies the T5a site only (Figure 4g).

**Table 1:** Lithium Atoms per Formula Unit on Wyckoff Sites in Li_6+*x*_P_1–*x*_Si_*x*_O_5_Cl Highlighting How Li Distribution Is Controlled through Composition

		compositions *F*4̅3*m*						
Li sites	Wyckoff site	0	0.1	0.3	0.5	0.6	0.7	0.75
T5a	24g	1.000	5.046(12)	3.126(6)	3.092(5)	2.736(8)	3.075(6)	3.048(5)
T5	48h		0.96(3)	2.824(13)	2.842(13)	3.25(2)	2.924(13)	2.894(10)
T3	4c		0.099(4)	0.070(4)	0.159(3)	0.164(4)	0.284(3)	0.209(3)
T4	16e			0.172(16)	0.353(3)	0.468(4)	0.360(3)	0.538(3)

		compositions *P*2_1_3		
Li sites	Wyckoff site	0.8	0.85	1
T5a	12b	1.533(17)	1.462(4)	3.000
	12b	1.432(18)	1.409(4)	
T5	12b	0.654(6)	0.639(8)	3.000
	12b	0.801(6)	0.873(7)	
	12b	0.531(6)	0.528(7)	
	12b	1.0302(9)	1.065(5)	
T3	4a	0.224(3)	0.240(3)	0.162(3)
T4	4a	0.23(2)	0.373(16)	0.838(1)
	12b	0.34(2)	0.243(6)	

When *x* is increased to 0.3–0.75, the relative occupancy of T5 over T5a sites increases (Table 1), enhancing the Li^+^ site disorder within the octahedral cages. This Li^+^ site disorder is strongly linked to increased ionic conductivity in sulfide argyrodites where disorder within the octahedral cages (T5 and T5a) is present in a large number of compositions (e.g., Li_6_PS_5_X where X = Cl or Br).^15^ Li_6+*x*_P_1–*x*_Si_*x*_O_5_Cl presents the first example of such Li^+^ site disorder in an oxide argyrodite at room temperature. The additional Li^+^ atoms (*x* = 0.3–0.75) introduced through heterovalent cation substitution occupy the T4 sites in addition to the T3 site occupied in *x* = 0.1 in a disordered manner. This unique combination of T5, T5a, T3, and T4 sites is analogous to the site occupancies observed in Li_7_SiO_5_Cl at 500 K (Figure 4h). The composition *x* = 0.7 is shown as an illustrative example for the compositional range *x* = 0.3–0.75 (Figure 4b,e) all of which crystallize in the *F*4̅3*m* space group and show T5, T5a, T3, and T4 sites with different site occupancies (Table 1).

As *x* is increased to 0.8, the material transitions from *F*4̅3*m* to *P*2_1_3 symmetry (Figure 4c). The transition between *P*2_1_3 and *F*4̅3*m* that occurs as a function of composition in Li_6+*x*_P_1–*x*_Si_*x*_O_5_Cl (at *x* = 0.8) is equivalent to the order–disorder transition observed at 472.1(6) K in Li_7_SiSO_5_Cl (Figure 4h,i).

Varied Li^+^ site occupancies in *x* = 0.8 and 0.85 (*P*2_1_3) for the T5 sites are obtained from refinements indicating partial ordering of the T5 positions (Table 1). The T4 (4a) site has a higher occupancy compared to other T4 (12b) sites, highlighting partial ordering of these T4 positions in *x* = 0.8 and 0.85 (Table 1). The Li^+^ site occupancies in *x* = 0.8 and 0.85 (Figure 4c,f) resemble those of Li_7_SiO_5_Cl at 300 K (Table 1, Figure 4i) in which only the T4 (4a) site is occupied and the T4 (12b) sites are empty, indicating that the order–disorder behavior of Li_7_SiO_5_Cl observed as a function of temperature is accessible in Li_6+*x*_P_1–*x*_Si_*x*_O_5_Cl through control of composition.

### 3.3 Transport Properties and Stability

#### 3.3.1 Ionic Transport

The ionic conductivity of Li_6+*x*_P_1.*x*_Si_*x*_O_5_Cl was investigated through AC impedance spectroscopy and static ^7^Li NMR spectroscopy. For impedance, pellets were prepared by reactive sintering (*x* = 0.1, 0.3, 0.5, 0.6, 0.7, 0.8, and 0.85) and by SPS (*x* = 0.7, 0.75, and 0.85). Pellets were polished, and SEM images were collected revealing the decreased porosity and the more homogeneous microstructure of pellets prepared via SPS compared to pellets prepared via reactive sintering (Figure S17). Using reactive sintering, ∼74–85% of the theoretical density was achieved, and SPS sintered pellets were ∼96% of the theoretical density. Samples prepared via SPS show conductivities similar to samples prepared via reactive sintering. For NMR, powdered samples were used.

**Figure 5:**
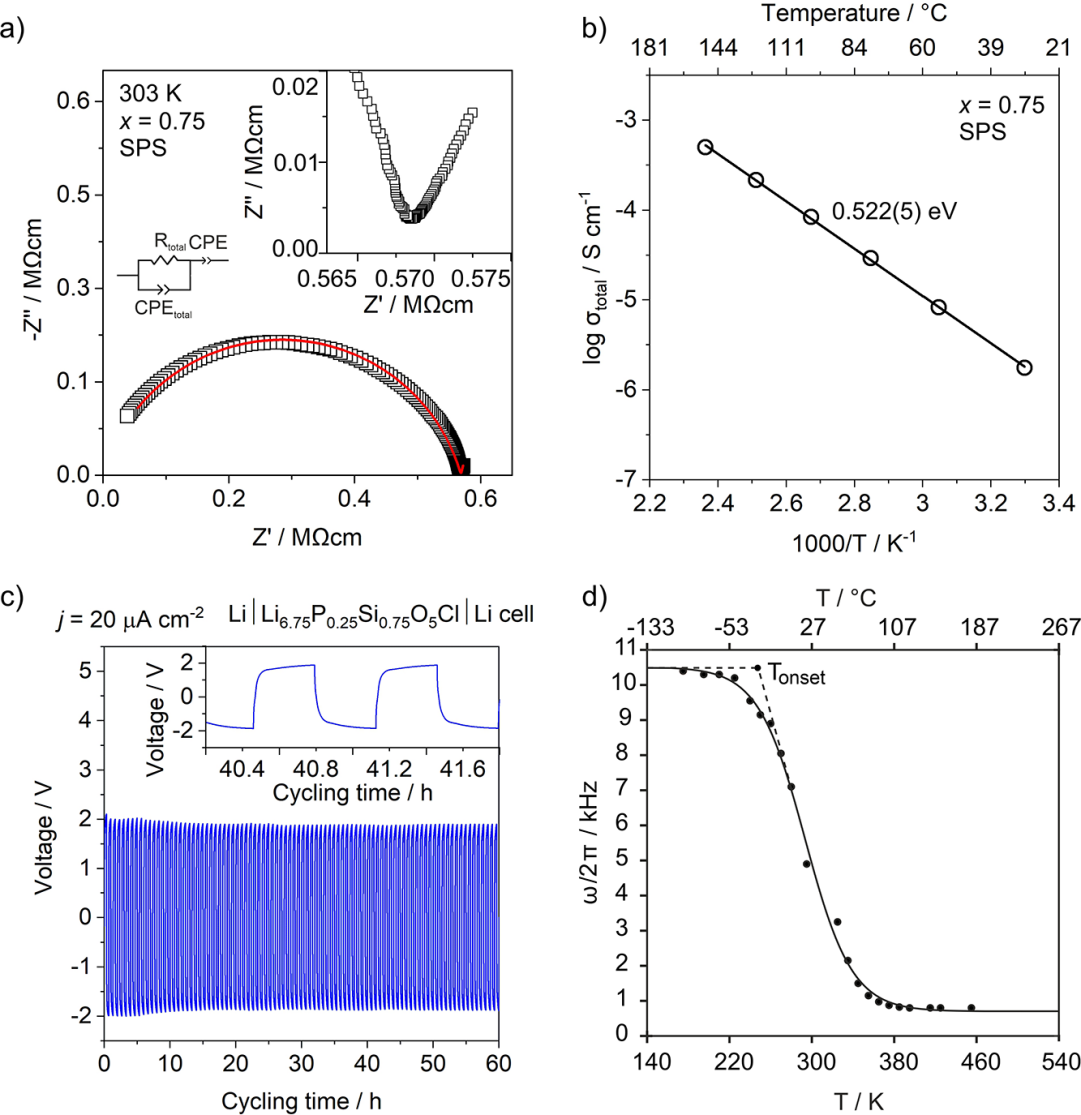
(a) Typical Nyquist plot for *x* = 0.75 of Li_6+*x*_P_1–*x*_Si_*x*_O_5_Cl prepared via SPS, inset highlighting the low-frequency spike characteristic of an electrode type response. (b) Arrhenius plot for *x* = 0.75 prepared via SPS. (c) Galvanostatic Li plating/stripping voltage profile of a symmetric Li|Li_6.75_P_0.25_Si_0.75_O_5_Cl|Li cell measured at 20 μA cm^–2^ at 298 K. (d) Temperature dependence of the NMR linewidth ω/2π of the ^7^Li central transition of Li_6.75_P_0.25_Si_0.75_O_5_Cl. The onset temperature of ^7^Li line narrowing is shown with dashed lines showing the tangents of the curve used to extract *T*_onset_. The solid black line is a fit to the data based on a sigmoidal regression given in eq S2 and is used to determine the inflection point of the curve. A comparison of the line narrowing curves for *x* = 0.3, 0.5, 0.75, and 1, as well as a comparison of the extracted Li-ion jump rates and activation energies are available in Figure S23 and Table S11, respectively.

A typical set of AC impedance data measured at 303 K in an inert atmosphere are shown in Figure 5a for *x* = 0.75 prepared via SPS (other compositions can be found in Figures S18a and S19). The impedance complex plane plots, Z*, consist of a high-frequency arc with the presence of a small low-frequency inclined spike (Figure 5a inset). The large single arc is attributed to the sample bulk, and this is confirmed by overlapping peaks in the combined *Z*″/*M*″ spectra (Figure S20). The capacitance of the high-frequency arc (1.7 pF cm^–1^) is also consistent with the bulk response. The low-frequency spike is indicative of an electrode-type response. To a first approximation, the data could be modeled using a single equivalent circuit. The *Z*″/*M*″ peaks are broader than expected for an ideal Debye peak,^43,44^ indicating the presence of a CPE (Figure S20). Therefore, total resistances, which correspond approximately to the bulk resistances, were obtained and used to calculate the total ionic conductivity for compositions in the *x* = 0.1–0.85 range (Table S10). The highest total ionic conductivity at 303 K was obtained for Li_6.75_P_0.25_Si_0.75_O_5_Cl prepared via SPS and calculated to be 1.82(1) × 10^–6^ S cm^–1^. DC polarization experiments were performed on several samples (*x* = 0.7 and 0.8 prepared via reactive sintering and SPS), and the electronic contribution was found to be <1.5% of the total conductivity (Figure S21).

The Arrhenius plot for the highest conducting sample *x* = 0.75 prepared via SPS is shown in Figure 5b with an extracted activation energy of 0.522(5) eV. This is lower compared to the Li^+^ site ordered Li_6_PO_5_Cl which has an activation energy of ∼0.60 eV^22^ and is the lowest activation energy for Li^+^ ion mobility reported for any oxide argyrodite.^22,23^ Arrhenius plots for all compositions (*x* = 0.1, 0.3, 0.5, 0.6, 0.7, 0.75, 0.8, and 0.85) alongside the calculated activation energies can be found in the Supporting Information (Figure S18b and Table S10).

Local Li^+^-ion mobility in Li_6+*x*_P_1–*x*_Si_*x*_O_5_Cl was probed by static ^7^Li NMR spectra that were collected over the 150–500 K temperature range for *x* = 0.3, 0.5, 0.75, and 1 (Figures 5d and S22 and S23). At low temperatures (<225 K), the ^7^Li NMR lineshape of the 1/2 ↔ 1/2 central transition displays a lineshape broadened by the strong ^7^Li–^7^Li homonuclear dipolar coupling. The static ^7^Li NMR linewidth at half-height (ω/2π)_rl_ in this so-called rigid lattice regime is approximately 10 kHz. As the sample temperature is increased, the linewidths of the central transition begins to significantly decrease at the onset temperatures *T*_onset_ for each composition, which is approx. 225 K for *x* = 0.75. This narrowing effect arises from the continuous averaging of the aforementioned strong ^7^Li–^7^Li homonuclear dipolar coupling due to the increased Li^+^-ion motion at frequencies exceeding ω/2π. Further heating above room temperature yields significantly narrower lines with ω/2π on the order of 800 Hz for *x* = 0.75. This corresponds to a lithium-ion jump rate τ^–1^ that greatly exceeds ω/2π, with a ^7^Li–^7^Li homonuclear dipolar coupling (fast motional regime) that is largely averaged out, and the residual linewidth is mainly governed by non-homonuclear dipolar interactions and inhomogeneities of the external magnetic field *B*_0_.^441^

Using a simple expression introduced by Waugh and Fedin^442^ (eq S1) correlating *T*_onset_ with *E*_a_ of the diffusion process, an approximate *E*_a_ value of 0.42 eV was estimated for *x* = 0.75, which is slightly lower than the value obtained from ACIS. This is to be somewhat expected as NMR spectroscopy determines the activation barrier for Li^+^-ion mobility to neighboring sites as well as unsuccessful Li^+^ ion hops over a much shorter length scale, whereas impedance measurements probe longer-range translational Li^+^ diffusion. Additionally, the inflection points of the line narrowing curves, *T*_inflection_, define the Li^+^-ion jump rate τ^–1^ which is of the order of (ω/2π)_rl_ and yields a value of 6.6(1) × 10^4^ s^–1^ for *x* = 0.75 at 290(3) K. The value of *T*_inflection_ was determined through fitting the linewidth data Boltzmann sigmoid regression curve (eq S2) (Figure 5d). A comparison of the static ^7^Li VT line narrowing profiles of the compositions *x* = 0.3, 0.5, 0.75, and 1 is available in Figure S22 where the corresponding τ^–1^ values and activation energies are listed in Table S11. The observed trend in the extracted activation energies agrees very well with that obtained through ACIS (Table S10).

Electrochemical stability of Li_6.75_P_0.25_Si_0.75_O_5_Cl against Li metal was tested on a pellet of the composition synthesized via SPS. The steady operation of galvanostatic Li plating/stripping at 20 μA cm^–2^ and 298 K over 60 h in a Li|Li_6.75_P_0.25_Si_0.75_O_5_Cl|Li symmetric cell confirms the reversible Li^+^ transport through the Li_6.75_P_0.25_Si_0.75_O_5_Cl solid electrolyte and the solid electrolyte/Li interface and good chemical compatibility between the solid electrolyte and Li metal (Figure 5c). The good stability of Li_6.75_P_0.25_Si_0.75_O_5_Cl against metallic lithium likely arises from stable structural units such as SiO_4_^4–^ and PO_4_^3–^.

#### 3.3.2 Stability in Air

The stability of the oxide argyrodite Li_6.7_P_0.3_Si_0.7_O_5_Cl to air was tested and compared to the sulfide argyrodite Li_6_PS_5_Cl. Li_6_PS_5_Cl completely decomposes within 1 h of air exposure as indicated by the loss of argyrodite peaks in the diffraction pattern (Figure S24). In comparison, the oxide argyrodite remains phase pure after 1 h of air exposure with no formation of impurity phases. The complete decomposition of Li_6_PS_5_Cl in the same time frame demonstrates the superior air and moisture stability of Li_6.7_P_0.3_Si_0.7_O_5_Cl. The XRD pattern of Li_6.7_P_0.3_Si_0.7_O_5_Cl recorded after 3 h of air exposure indicates slow decomposition observed by the broadening of the highlighted argyrodite peaks and an increase in the intensity of impurity phase peaks. The most intense cubic argyrodite peaks are still present after 60 h but are significantly broadened, and the intensity of peaks corresponding to impurity phases has increased significantly. Analysis of the XRD pattern after 60 h of exposure of air shows the presence of Li_2_CO_3_ in Li_6.7_P_0.3_Si_0.7_O_5_Cl indicating that the oxide argyrodite reacts with CO_2_ in the atmosphere to form lithium carbonate. The severe instability of highly conductive sulfide argyrodites such as Li_6_PS_5_Cl to air and moisture is one of their limiting factors,^45^ so the superior air stability offered by oxide argyrodites enables potential routes to processing and handling for scale up and use in devices for which argyrodite solid electrolytes were previously not suitable.

### 3.4 Structure–Property Relationship

**Figure 6:**
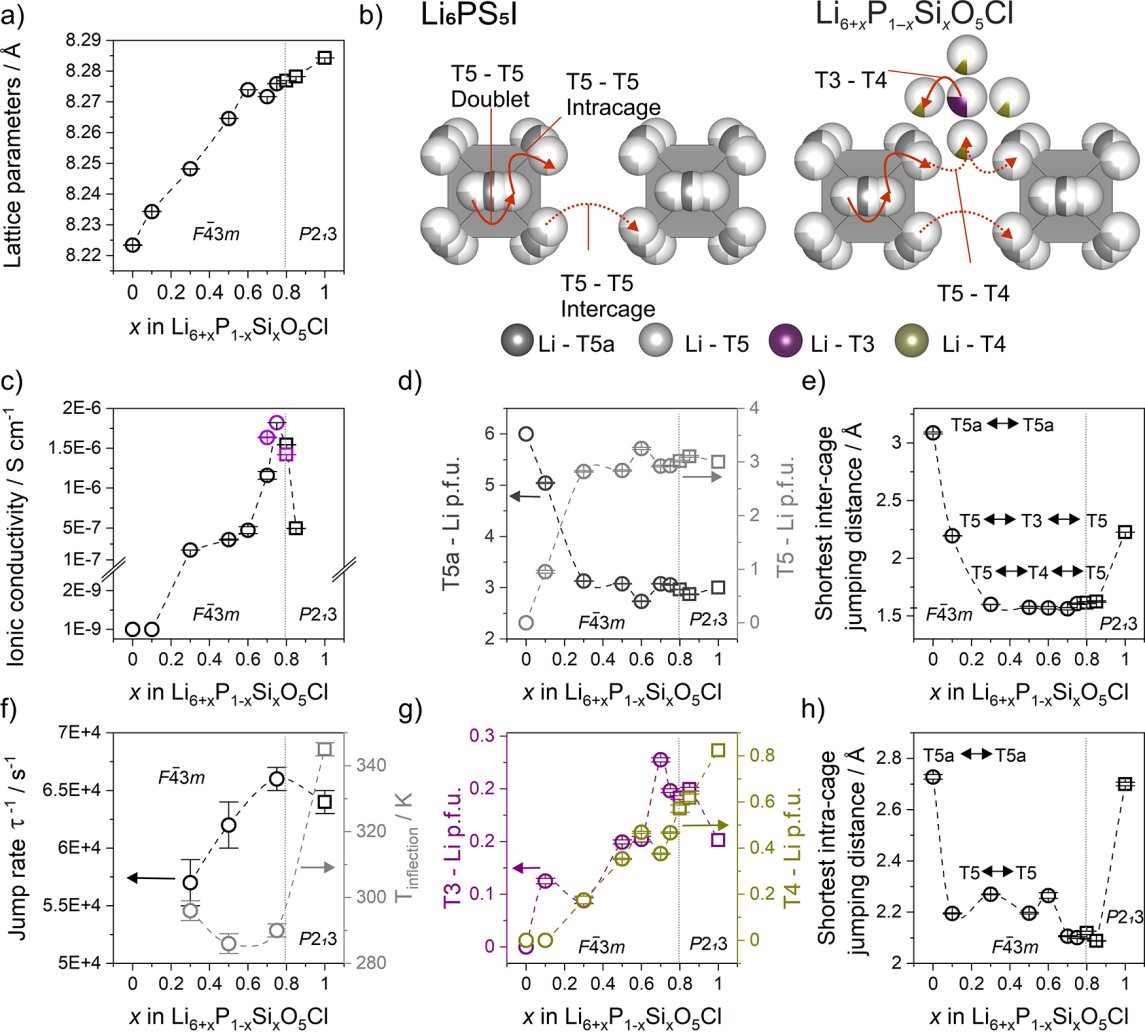
(a) Lattice parameters as a function of *x* in Li_6+*x*_P_1–*x*_Si_*x*_O_5_Cl. (b) T5–T5 Li^+^-ion pathways in Li^+^ argyrodite Li_6_PS_5_I and alternative Li^+^-ion pathways in Li_6+*x*_P_1–*x*_Si_*x*_O_5_Cl. (c) Total ionic conductivity as a function of *x* in Li_6+*x*_P_1–*x*_Si_*x*_O_5_Cl on which samples prepared via reactive sintering and SPS are shown in black and purple, respectively. (d) T5 and T5a Li^+^ atoms per formula unit, (e) shortest intercage jumping distances, and (f) Li^+^-ion jump rate and *T*_inflection_ determined through ^7^Li NMR. (g) T3 and T4 Li^+^ atoms per formula unit and (h) shortest intracage jumping distances as a function of *x* in Li_6+*x*_P_1–*x*_Si_*x*_O_5_Cl. Compositions crystallizing in *F*4̅3*m* symmetry are shown as circles, and compositions crystallizing in *P*2_1_3 symmetry are shown as squares.

Optimizing both the Li^+^ and anion sublattices can lead to substantial improvements in ionic conductivity in Li^+^ argyrodites. The connectivity of Li^+^ sites both within and between the octahedral Li^+^ cages, formed from T5 and T5a sites surrounding the central anion (4d), is considered as one of the limiting factors for ionic conductivity in Li^+^ argyrodites.^46^ The Li^+^-ion pathways in Li^+^ argyrodites consist of T5–T5 intercage and T5–T5 intracage jumps which form a 3D network throughout the structure (Figure 6b).^47^ Recently, the importance of additional Li^+^ sites (T2, T3, and T4) which facilitate long-range Li^+^ diffusion by providing shorter distances between occupied Li^+^ sites have been highlighted in sulfide argyrodites.^18^ For example, incorporating additional Li^+^ into Li_6_PS_5_I on the T2 and T4 sites alongside T5 and T5a positions increases the ionic conductivity from ∼10^–6^ S cm^–1^ to ∼10^–2^ S cm^–1^ in Li_6+*x*_P_1–*x*_Ge_*x*_S_5_I.^16^ The other main factor controlling the ionic conductivity in argyrodites is the disorder on the anion sublattice, for example, increasing the site mixing of sulfide and bromide anions leads to a 4-fold increase in ionic conductivity in Li_6_PS_5_Br.^19,48^

Four separate Li^+^ sites are occupied in Li_6+*x*_P_1–*x*_Si_*x*_O_5_Cl: the T5 and T5a sites which form the octahedral cages centered around O^2–^ anions (4d) and the T3 and T4 sites located in between neighboring octahedral cages (Figure 6b). The distribution of Li^+^ across these sites as a function of *x* (Figure 6d,g) is strongly correlated with the trend in lattice parameters (Figure 6a), Li^+^-ion jumping distances (Figure 6e,h), and the ionic conductivity (Figure 6c,f).

The ionic conductivity of *x* = 0.1 is low (∼10^–9^ S cm^–1^), similar to Li_6_PO_5_Cl which corresponds to *x* = 0 (Figure 6c). Li_6_PO_5_Cl adopts a fully Li^+^ site ordered structure, and all six Li^+^ atoms per formula unit (p.f.u.) reside on the T5a site.^22^ In *x* = 0.1, disorder is introduced with 1.1 Li^+^ atoms p.f.u. on the T5 site and 4.9 Li^+^ atoms occupying the T5a site (Figure 6d). The additional 0.1 Li^+^ atoms occupy the T3 site, which introduces a shorter intercage pathway with shorter T5–T3 jumps compared to the larger T5a–T5a intercage jumps in Li_6_PO_5_Cl (Figure 6e). Intracage jumping distances are also shortened when *x* is increased from 0 to 0.1, resulting from the (low) occupancy of T5 sites in *x* = 0.1 compared to the sole occupancy of T5a sites in Li_6_PO_5_Cl (Figure 6h, Table S12); however, due to the low occupancies of T5 and T3 sites, the ionic conductivity remains low for *x* = 0.1.

As *x* is increased to 0.3, the ionic conductivity increases by 2 orders of magnitude from ∼10^–9^ to 2.2 × 10^–7^ S cm^–1^ (Figure 6c). This sudden increase in ionic conductivity can be attributed to the increased disorder across all Li^+^ sites. In *x* = 0.3–0.6, approximately equal amounts of Li^+^ atoms occupy both the T5 and T5a sites (Figure 6d) which facilitates more intracage mobility. Furthermore, Li^+^ ions partially occupy the T4 site alongside the T3 site (Figure 6g). Occupancy of the T4 site provides an alternative pathway for Li^+^-ion movement consisting of shorter intercage Li^+^ ion jumps through face-sharing tetrahedral environments. In addition to the T5–T5 intercage jumps through corner sharing tetrahedra which exist in most Li^+^ argyrodites, Li^+^ ions are also connected to neighboring octahedral cages via T5–T4–T5 or T5–T4–T3–T4–T5 jumps in Li_6+*x*_P_1–*x*_Si_*x*_O_5_Cl. The T5–T4 and T3–T4 distances are significantly shorter than the T5–T5 intercage distances (Figure 6b,e; Table S12) and consist of face-sharing tetrahedra facilitating Li-ion motion between octahedral cages. This alternative pathway increases the long-range connectivity of octahedral cages and leads to the observed improvement in transport properties. The ionic conductivity increases slightly for *x* = 0.5 and 0.6 (Figure 6c) as the Li^+^ content increases, and Li^+^ atoms preferentially occupy sites directly involved in intercage jumps (i.e., T5 and T4 sites) (Figure 6d,g).

When *x* = 0.7, the ionic conductivity increases further to 1.4 × 10^–6^ S cm^–1^ (Figure 6c), and the lithium-ion jump rate τ^–1^ is at its highest (Figure 6f). Coinciding with this sudden increase, the lattice parameters, which increase linearly for *x* = 0–0.6, decrease unexpectedly (Figure 6a) accompanied by changes in relative Li^+^ site occupancies (Figure 6d,g). At *x* = 0.7, the number of Li^+^ atoms occupying the T5a and T3 sites increases relative to the T5 and T4 sites in contrast to the trend observed for *x* < 0.7 potentially resulting from increased electrostatic repulsion as an increasing number of Li^+^ atoms are added to the lattice (Figure 6d,g). This preferential occupancy of T5a and T3 sites over T4 and T5 sites leads to a contraction of the octahedral lithium-ion cages as shown by reduced T5–T5 intracage distances (Figure 6h, Table S12), leading ultimately to the observed decrease in lattice parameters (Figure 6a). This leads to the observed increase in the ^6^Li NMR linewidths (Figure 3d) from 50 Hz in *x* = 0.3 to 130 Hz in *x* = 0.75 as the number of partially occupied Li^+^ sites increases, directly increasing the extent of inhomogeneous broadening. This correlates with the increase in ionic conductivity (Figure 6f) and the changes in relative site occupancies (Figure 6d,g).

The behavior observed in Li_6+*x*_P_1–*x*_Si_*x*_O_5_Cl is distinctive to sulfide argyrodites. Recent studies suggest that an expansion of the octahedral cages is beneficial to the ionic conductivity in sulfide argyrodites as this shortens the intercage jumping distances usually considered to be the rate-limiting step for lithium-ion diffusion in Li^+^ argyrodites.^19^ In Li_6+*x*_P_1–*x*_Si_*x*_O_5_Cl, the introduction of partially occupied T4 sites, even at low values of *x*, shortens these crucial intercage distances significantly, and the ionic conductivity is further enhanced only when the intracage jumping distances are also shortened at *x* = 0.7 and above. This highlights the importance of both inter- and intracage jumps within oxide argyrodite materials and yields the maximum ionic conductivity of 1.82(1) × 10^–6^ S cm^–1^ for *x* = 0.75 in Li_6+*x*_P_1–*x*_Si_*x*_O_5_Cl prepared via SPS.

When *x* > 0.75, the ionic conductivity decreases for *x* = 0.8 (1.54(2) × 10^–6^ S cm^–1^) and 0.85 (4.93(4) × 10^–7^ S cm^–1^) (Figure 6c). The inter- and intracage Li^+^-jumping distances in *x* = 0.8 and 0.85 are almost identical to *x* = 0.3–0.75 (Figure 6e,h). The sudden drop in conductivity at *x* = 0.8 coincides with the change in structural symmetry from *F*4̅3*m* to *P*2_1_3 and the onset of Li^+^ site ordering. A minimum critical total occupancy of the T3 and T4 sites is reached and results in this site ordering which consequently affects the ordering on the T5 and T5a sites, resulting in a more inhomogeneous lithium distribution in *x* = 0.8 and 0.85. This is analogous to the complete ordering of Li^+^ sites observed in Li_7_SiO_5_Cl (*x* = 1), resulting in significantly longer intra- and intercage jumping distances (Figure 6e,h; Table S12), and is the reason for the dramatic reduction in ionic conductivity and lithium-ion jump rate at *x* > 0.8 (Figure 6c,f).

## 4 Conclusions

We present the first cubic Li-rich oxide argyrodite, Li_7_SiO_5_Cl, and solid solution Li_6+*x*_P_1–*x*_Si_*x*_O_5_Cl. Li_7_SiO_5_Cl crystallizes as a Li^+^ site ordered cubic oxide argyrodite (*P*2_1_3) at room temperature and exhibits order–disorder behavior at elevated temperatures analogous to disordered *F*4̅3*m* sulfide argyrodites. The high-temperature *F*4̅3*m* structure of Li_7_SiO_5_Cl represents the first Li argyrodite in which simultaneous occupation of T5, T5a, T3, and T4 sites is observed. By controlling composition via cation substitution, the Li_6+*x*_P_1–*x*_Si_*x*_O_5_Cl solid solution between the ordered end members, Li_6_PO_5_Cl and Li_7_SiO_5_Cl, stabilizes a highly disordered Li^+^ distribution to room temperature, producing the Li^+^ site disorder in an oxide argyrodite previously only seen in sulfides. The Li^+^ distribution in these novel oxide argyrodites differs from the Li^+^ sites reported for sulfide argyrodites and gives rise to a distinctive structure–property relationship in which the Li^+^ distribution over T5, T5a, T4, and T3 sites controls the transport properties through the creation of face-sharing tetrahedral pathways. Full structural understanding was crucial to elucidate the underlying mechanism responsible for controlling the ionic conductivity in these materials. The ionic conductivity, which is low (∼10^–9^ S cm^–1^) for small values of *x*, increases by 3 orders of magnitude to a maximum value of 1.82(1) × 10^–6^ S cm^–1^ at *x* = 0.75. The significant change in transport properties can be attributed to the introduction of Li^+^ site disorder on the octahedral Li^+^ cages as well as a unique combination of partially occupied Li^+^ sites, which affords a pathway for Li^+^-ion diffusion that consists of face-sharing tetrahedra connecting neighbouring octahedral Li^+^ ion cages via T5–T4–T3–T4–T5 and T5–T4–T5 jumps. This increases the connectivity of octahedral cages and facilitates long-range Li^+^-ion movement. The new structural motif defined by the Li^+^ site occupancy in the oxide argyrodites gives distinct control of properties by composition from that in sulfide argyrodites. The materials exhibit superior air stability compared to sulfide argyrodites and are stable against Li metal.

The heterovalent cation substitution reported here shows the extent of chemical space yet to be explored in the field of oxide argyrodites. Building on the wealth of chemical knowledge already established in the mature field of sulfide Li^+^ argyrodites, many routes can be explored to further increase the transport properties, making this a promising area of research in the field of solid-state electrolytes.

## Supporting Information Available

PXRD patterns highlighting effect of ball milling and dehydrated starting materials on the phase purity of Li_6+*x*_P_1–*x*_Si_*x*_O_5_Cl, DSC and lattice parameter changes as a function of temperature in Li_7_SiO_5_Cl, structural information of Li_6+*x*_P_1–*x*_Si_*x*_O_5_Cl determined by single-crystal diffraction and powder diffraction refinement (refinement details, atomic positions, bond distances, and angles), Pawley and Rietveld fits of Li_6+*x*_P_1–*x*_Si_*x*_O_5_Cl, comparison of Rietveld refinement before and after refinement of T3 and T4 positions, ICP and SEM–EDX results for Li_6+*x*_P_1–*x*_Si_*x*_O_5_Cl, ^29^Si MAS NMR, ^31^P MAS NMR, and static ^7^Li NMR spectra as a function of temperature, SEM images, impedance results, and PXRD patterns showing air stability (PDF)
Crystal structures of Li_6.1_P_0.9_Si_0.1_O_5_Cl, Li_6.3_P_0.7_Si_0.3_O_5_Cl, Li_6.5_P_0.5_Si_0.5_O_5_Cl, Li_6.6_P_0.4_Si_0.6_O_5_Cl, Li_6.7_P_0.3_Si_0.7_O_5_Cl, Li_6.75_P_0.25_Si_0.75_O_5_Cl, Li_6.8_P_0.2_Si_0.8_O_5_Cl, and Li_6.85_P_0.15_Si_0.85_O_5_Cl are deposited with ICSD 2182331, 2182521, 2182520, 2182522, 2182526, 2182525, 2182524, and 2182523, respectively. Crystal structures of Li_7_SiO_5_Cl at 100, 300, and 500 K are deposited with ICSD 2183068, 2183067, and 2183066, respectively

## Notes

Data availability: Underlying data collected as part of this work are available via the University of Liverpool data repository at https://datacat.liverpool.ac.uk/id/eprint/1741.
