# Supplementary material for: Control of Ionic Conductivity by Lithium Distribution in Cubic Oxide Argyrodites Li6+xP1–xSixO5Cl
Source: J Am Chem Soc. 2022 Nov 22;144(48):22178–92. doi: 10.1021/jacs.2c09863 (PMC9732874; doi:10.1021/jacs.2c09863)
Supplement: Supplementary file 1 — ja2c09863_si_001.pdf [file ja2c09863_si_001.pdf]

Supplementary Information

Control of ionic conductivity by lithium distribution in the cubic oxide argyrodites $Li_{6+x}P_{1-x}Si_xO_5Cl$

Alexandra Morscher,[a] Benjamin B. Duff,[a,b] Guopeng Han,[a] Luke M. Daniels,[a] Yun Dang,[a] Marco Zanella,[a] Manel Sonni,[a] Ahmad Malik,[a] Matthew S. Dyer, [a] Ruiyong Chen,[a] Frédéric Blanc,[a,b] John B. Claridge[a] and Matthew J. Rosseinsky*[a]

[a] Department of Chemistry, University of Liverpool, Crown Street, L69 7ZD Liverpool, UK.

[b] Stephenson Institute for Renewable Energy, University of Liverpool, Peach Street L69 7ZF Liverpool, UK.

*Corresponding Author: m.j.rosseinsky@liverpool.ac.uk

Table S1: Exact masses of precursors used in the synthesis of $Li_{6+x}P_{1-x}Si_xO_5Cl$

	$x = 0.1$	$x = 0.3$	$x = 0.5$	$x = 0.6$	$x = 0.7$	$x = 0.75$	$x = 0.8$	$x = 0.85$
**Li_4SiO_4 / g**	0.0654	0.1899	0.3152	0.3775	0.4394	0.4703	0.5012	0.5319
**Li_3PO_4 / g**	0.5529	0.4282	0.3046	0.2431	0.1820	0.1515	0.1211	0.0907
**Li_2O / g**	0.1585	0.1579	0.1572	0.1569	0.1565	0.1564	0.1562	0.1560
**LiCl / g**	0.2249	0.2240	0.2230	0.2225	0.2221	0.2218	0.2216	0.2214

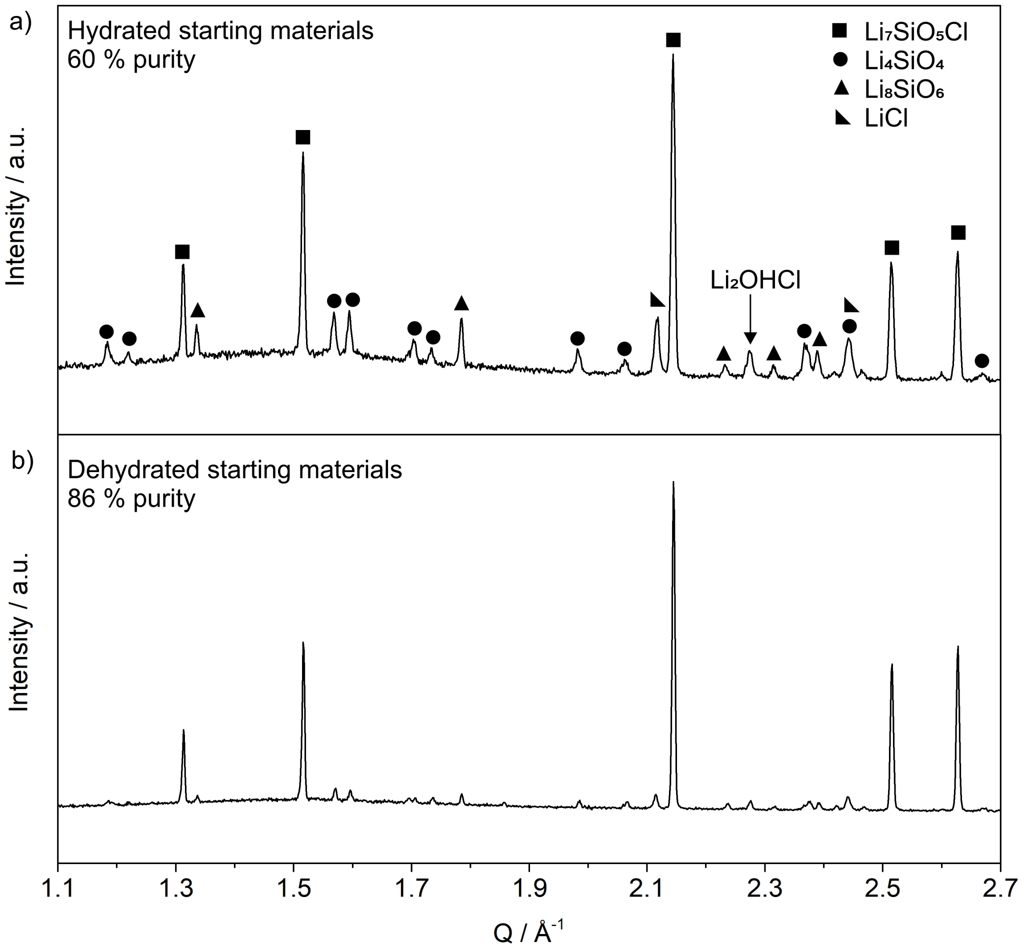

Figure S1: Comparison of XRD patterns and extracted sample purity for powder samples of Li_7SiO_5Cl when using (a) hydrated and (b) dehydrated Li_4SiO_4, Li_2O and LiCl precursors.

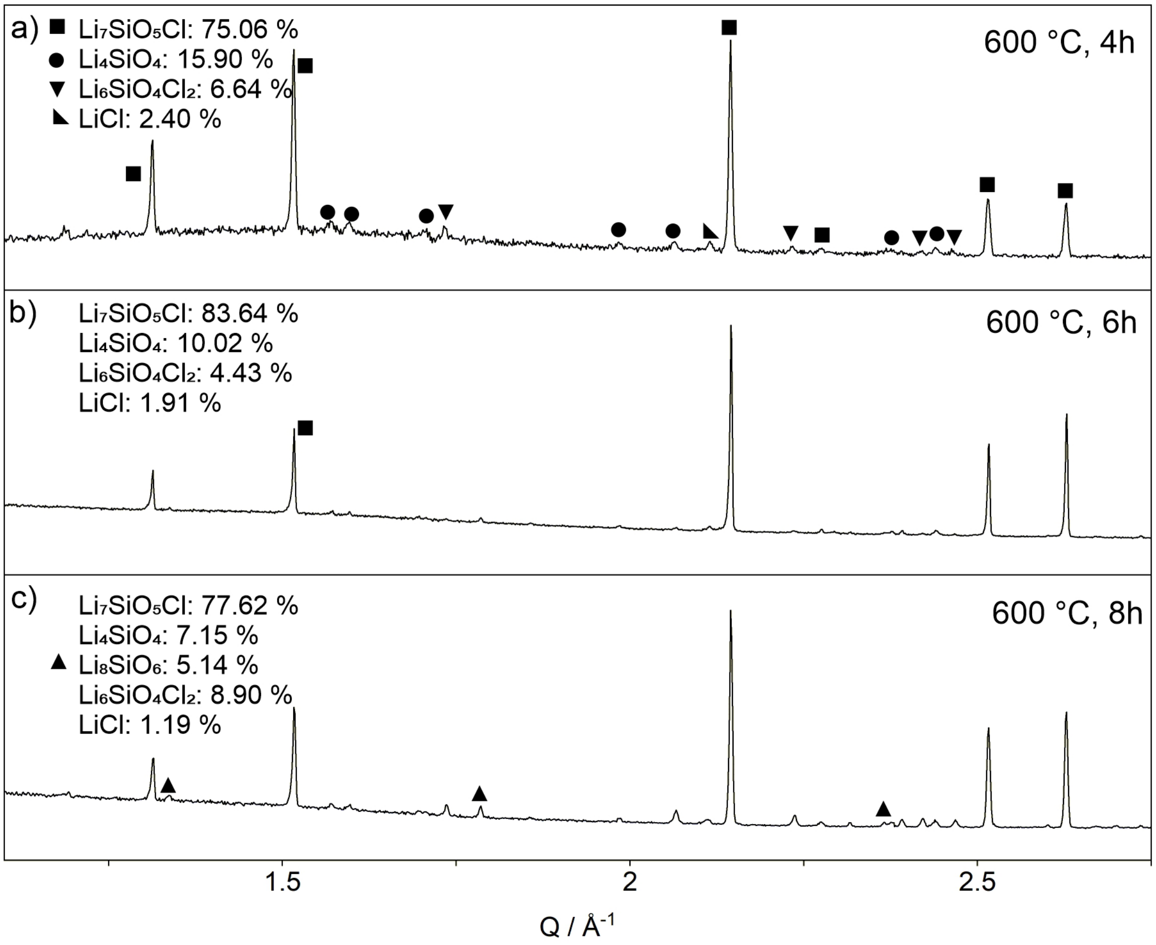

Figure S2: Comparison of XRD patterns and extracted weighted phase fraction for powder samples of Li_7SiO_5Cl when annealing for (a) 4h, (b) 6h and (c) 8h at 600 °C. The reaction reaches equilibrium at an annealing time of 6 h with a maximum phase fraction of 83.64 % for Li_7SiO_5Cl. Shorter reaction times lead to the presence of larger amounts of starting materials (Li_4SiO_4, LiCl) indicating incomplete reaction, whilst longer reaction times lead to the formation of decomposition products (Li_8SiO_6, $Li_6SiO_4Cl_2$)

Table S2. Single crystal data, data collection and structure refinement parameters of Li_7SiO_5Cl.

**Empirical formula**	**Li_7SiO_5Cl**					
Formula weight	192.12					
Radiation	MoKα (λ = 0.71073 Å)			synchrotron (λ = 0.6889 Å)		
Temperature / K	100.0	291.2	500.0	100.0	300.0	500.0
Crystal system, Space group	cubic, $P2_13$	cubic, $P2_13$	cubic, $F\bar{4}3m$	cubic, $P2_13$	cubic, $P2_13$	cubic, $F\bar{4}3m$
a / Å	8.26050(10)	8.2697(2)	8.2972(2)	8.25320(10)	8.27250(10)	8.3032(2)
Volume / Å^3	563.66(2)	565.55(4)	571.21(4)	562.17(2)	566.12(2)	572.45(4)
Z	4	4	4	4	4	4
Reflections collected/ Independent reflections	16881/439 [R_{int} = 0.0486]	9973/545 [R_{int} = 0.0458]	2705/111 [R_{int} = 0.0297]	10801/726 [R_{int} = 0.0578]	11212/726 [R_{int} = 0.0525]	2730/139 [R_{int} = 0.0982]
Crystal size / mm	0.11 × 0.07 × 0.07			0.03 × 0.02 × 0.02		
Data/restraints/ parameters	439/0/44	545/0/48	111/1/20	726 /0/44	726/0/48	139/1/20
Goodness-of-fit on F^2	1.224	1.046	1.170	1.114	1.136	1.113

Completeness / %	100	100	100	100	100	100
Twin ratio	0.509(3):0.491(3)	0.506(4):0.494(4)	—	0.653(3):0.347(3)	0.655(3):0.345(3)	—
Final *R* indexes [I≥2σ (I)]	R_1 = 0.0163, wR_2 = 0.0381	R_1 = 0.0223, wR_2 = 0.0526	R_1= 0.0179, wR_2 = 0.0456	R_1 = 0.0238, wR_2 = 0.0605	R_1 = 0.0229, wR_2 = 0.0603	R_1 = 0.0423, wR_2 = 0.1019
Final *R* indexes [all data]	R_1 = 0.0163, wR_2 = 0.0381	R_1 = 0.0251, wR_2 = 0.0533	R_1 = 0.0179, wR_2 = 0.0456	R_1 = 0.0239, wR_2 = 0.0607	R_1 = 0.0231, wR_2 = 0.0605	R_1 = 0.0424, wR_2 = 0.1020
Largest diff.peak/hole / e Å^{-3}	0.17/-0.46	0.21/-0.31	0.15/-0.37	0.61/-0.24	0.47/-0.21	0.41/-0.30
Flack parameter	-0.38(4)	-0.38(6)	0.04(6)	-0.19(5)	-0.25(5)	-0.3(3)

Table S3a. Fractional atomic coordinates (×10^4) and equivalent isotropic displacement parameters (Å^2×10^3) for Li_7SiO_5Cl at 100 K. U_{eq} is defined as 1/3 of the trace of the orthogonalised U_{IJ} tensor.

**Site**	***wyck.***	***sof***	***x***	***y***	***z***	**U(eq)**
Li1	12*b*	1	7100(4)	7033(4)	10342(3)	15.6(6)
Li2	12*b*	1	4934(5)	7630(4)	7395(4)	15.4(6)
Li3	4*a*	1	8445(4)	8445(4)	8445(4)	18.3(11)
Si1	4*a*	1	5060.1(4)	9939.9(4)	10060.1(4)	6.30(17)
O1	4*a*	1	3924.9(12)	11075.1(12)	8924.9(12)	8.5(4)
O2	12*b*	1	6274.9(12)	8853.7(14)	8925.8(13)	8.7(2)
O3	4*a*	1	7559.6(13)	7440.4(13)	12559.6(13)	8.0(4)
Cl1	4*a*	1	5098.5(4)	4901.5(4)	9901.5(4)	9.40(14)

Table S3b Fractional atomic coordinates (×10^4) and equivalent isotropic displacement parameters (Å^2×10^3) for Li_7SiO_5Cl at 300 K. U_{eq} is defined as 1/3 of the trace of the orthogonalised U_{IJ} tensor.

**Site**	***wyck.***	***sof***	***x***	***y***	***z***	**U(eq)**
Li1	12*b*	1	2839(3)	4556(4)	386(4)	25.1(7)
Li2	12*b*	1	2425(6)	94(4)	117(4)	24.6(7)
Li3	4*a*	0.840(15)	934(4)	5934(4)	-934(4)	20.3(15)
Li4	4*a*	0.160(15)	70(20)	5070(20)	-70(20)	13(7)
Si1	4*a*	1	2553.7(4)	7446.3(4)	2446.3(4)	9.22(18)
O1	4*a*	1	1419.0(12)	8581.0(12)	3581.0(12)	12.7(4)
O2	12*b*	1	1419.4(12)	6354.6(13)	1241.6(11)	12.8(2)
O3	4*a*	1	5055.6(14)	4944.4(14)	-55.6(14)	11.9(4)
Cl1	4*a*	1	2411.4(5)	2411.4(5)	2411.4(5)	16.35(16)

Table S3c. Fractional atomic coordinates (×10^4) and equivalent isotropic displacement parameters (Å^2×10^3) for Li_7SiO_5Cl at 500 K. U_{eq} is defined as 1/3 of the trace of the orthogonalised U_{IJ} tensor.

**Site**	***wyck.***	***sof***	***x***	***y***	***z***	**U(eq)**
Li1	4*d*	0.4434(12)	7500	2500	2500	14(6)
Li2	48*h*	0.4835(1)	7730(30)	5180(20)	2730(20)	55(9)
Li3	16*e*	0.1886(3)	8460(90)	3460(90)	3460(90)	40(20)
Si1	4*b*	1	5000	5000	5000	21.2(7)
O1	16*e*	1	6138(3)	3862(3)	3862(3)	25.9(9)
O2	4*c*	1	7500	7500	2500	27.1(16)
Cl1	4*a*	1	10000	5000	5000	36.0(8)

Table S4a. Anisotropic displacement parameters ($Å^2 \times 10^3$) for Li_7SiO_5Cl at 100 K. The anisotropic displacement factor exponent takes the form: $-2\pi^2[h^2a^{*2}U_{11}+2hka^*b^*U_{12}+\ldots]$.

Site	U_{11}	U_{22}	U_{33}	U_{23}	U_{13}	U_{12}
Li1	14.4(12)	18.7(15)	13.8(13)	-2.5(11)	-0.7(11)	2.9(12)
Li2	13.1(14)	19.1(16)	13.8(14)	-4.2(10)	-1.2(11)	-0.8(10)
Li3	18.3(11)	18.3(11)	18.3(11)	2.7(12)	2.7(12)	2.7(12)
Si1	6.30(17)	6.30(17)	6.30(17)	-0.08(15)	0.08(15)	-0.08(15)
O1	8.5(4)	8.5(4)	8.5(4)	0.6(4)	-0.6(4)	0.6(4)
O2	8.6(5)	8.3(5)	9.2(5)	0.1(5)	0.6(4)	0.3(4)
O3	8.0(4)	8.0(4)	8.0(4)	-0.2(3)	0.2(3)	-0.2(3)
Cl1	9.40(14)	9.40(14)	9.40(14)	-0.04(17)	0.04(17)	0.04(17)

Table S4b Anisotropic Displacement Parameters ($Å^2 \times 10^3$) for Li_7SiO_5Cl at 300 K. The anisotropic displacement factor exponent takes the form: $-2\pi^2[h^2a^{*2}U_{11}+2hka^*b^*U_{12}+\ldots]$

Site	U_{11}	U_{22}	U_{33}	U_{23}	U_{13}	U_{12}
Li1	18.1(13)	31.2(16)	25.9(14)	-6.1(13)	1.8(12)	-2.2(12)
Li2	19.0(15)	23.9(16)	31.0(18)	9.8(11)	-2.5(10)	-1.3(11)
Li3	20.3(15)	20.3(15)	20.3(15)	-2.4(12)	-2.4(12)	2.4(12)
Li4	13(7)	13(7)	13(7)	5(4)	5(4)	-5(4)
Si1	9.22(18)	9.22(18)	9.22(18)	0.34(16)	-0.34(16)	-0.34(16)
O1	12.7(4)	12.7(4)	12.7(4)	-0.6(4)	0.6(4)	0.6(4)
O2	13.5(5)	12.3(5)	12.7(5)	-1.1(4)	-1.6(4)	-1.0(4)
O3	11.9(4)	11.9(4)	11.9(4)	-0.1(3)	0.1(3)	0.1(3)
Cl1	16.35(16)	16.35(16)	16.35(16)	-0.17(18)	-0.17(18)	-0.17(18)

Table S4c. Anisotropic displacement parameters ($Å^2 \times 10^3$) for Li_7SiO_5Cl at 500 K. The anisotropic displacement factor exponent takes the form: $-2\pi^2[h^2a^{*2}U_{11}+2hka^*b^*U_{12}+\ldots]$.

Site	U_{11}	U_{22}	U_{33}	U_{23}	U_{13}	U_{12}
Li1	14(6)	14(6)	14(6)	0	0	0
Li2	57(12)	53(9)	57(12)	13(7)	22(14)	13(7)
Li3	40(20)	40(20)	40(20)	7(19)	7(19)	7(19)
Si1	21.1(7)	21.1(7)	21.1(7)	0	0	0
O1	25.9(9)	25.9(9)	25.9(9)	-2.9(11)	2.9(11)	2.9(11)
O2	27.0(16)	27.0(16)	27.0(16)	0	0	0
Cl1	36.0(8)	36.0(8)	36.0(8)	0	0	0

Table S5a. Selected bond lengths (Å) and bond angles (˚) for Li_7SiO_5Cl from single crystal X-ray diffraction at 100 K.

Atoms	Distance / Å	Atoms	Angles / °
Li1—O2	2.042(3)	O2—Li1—O2	97.38(15)
	2.022(3)	O2—Li1—Cl1	100.90(13)
Li1—O3	1.899(3)		102.79(12)
Li1—Cl1	2.440(3)	O3—Li1—O2	119.51(17)
Li2—O1	1.929(4)		119.13(16)
Li2—O2	1.960(3)	O3—Li1—Cl1	113.98(14)
Li2—O3	1.961(4)	O1—Li2—O2	115.5(2)
Li2—Cl1	2.666(3)	O1—Li2—O3	118.61(16)
Li3—O2	1.865(2)	O1—Li2—Cl1	94.08(12)
Li3—Cl1	2.364(5)	O2—Li2—O3	124.6(2)
Si1—O1	1.6227(18)	O2—Li2—Cl1	95.84(13)
Si1—O2	1.6387(13)	O3—Li2—Cl1	91.41(16)
		O2—Li3—O2	109.86(16)
		O2—Li3—Cl1	109.08(16)
		O1—Si1—O2	109.83(4)
		O2—Si1—O2	109.11(4)

Table S5b. Selected bond lengths (Å) and bond angles (˚) for Li_7SiO_5Cl from single crystal X-ray diffraction at 300 K.

Atoms	Distance / Å	Atoms	Angles / °
Li1—O2	2.043(3)	O2—Li1—O2	98.29(15)
	2.023(3)	O2—Li1—Cl1	100.25(13)
Li1—O3	1.897(3)		102.02(13)
Li1—Cl1	2.466(4)	O3—Li1—O2	119.65(17)
Li2—O1	1.932(4)		120.26(17)
Li2—O2	1.958(3)	O3—Li1—Cl1	113.05(15)
Li2—O3	1.961(5)	O1—Li2—O2	115.3(2)
Li2—Cl1	2.698(4)	O1—Li2—O3	119.16(16)
Li3—O2	1.876(2)	O1—Li2—Cl1	93.52(12)
Li3—Cl1	2.371(5)	O2—Li2—O3	124.5(2)
Li4—O1	2.14(3)	O2—Li2—Cl1	95.08(13)
Li4—O2	1.883(10)	O3—Li2—Cl1	91.46(17)
Si1—O1	1.6259(19)	O2—Li3—O2	110.09(16)
Si1—O2	1.6399(13)	O2—Li3—Cl1	108.84(17)
		O2—Li4—O1	109.4(8)
		O2—Li4—O2	109.6(8)
		O1—Si1—O2	109.78(3)
		O2—Si1—O2	109.16(3)

Table S5c. Selected bond lengths (Å) and bond angles (˚) for Li_7SiO_5Cl from single crystal X-ray diffraction at 500 K.

Atoms	Distance / Å	Atoms	Angles / °
Li1—O1	1.959(5)	O1—Li1—O1	109.471(1)
Li2—O1	1.956(13)	O1—Li2—O1	109.7(11)
Li2—O2	1.945(19)	O1—Li2—Cl1	96.1(8)
Li2—Cl1	2.67(3)	O2—Li2—O1	122.4(6)
Li3—O1	1.99(5)	O2—Li2—Cl1	101.1(11)
Li3—Cl1	2.21(13)	O1—Li3—O1	107(4)
Si1—O1	1.637(5)	O1—Li3—Cl1	112(3)
		O1—Si1—O1	109.471(1)

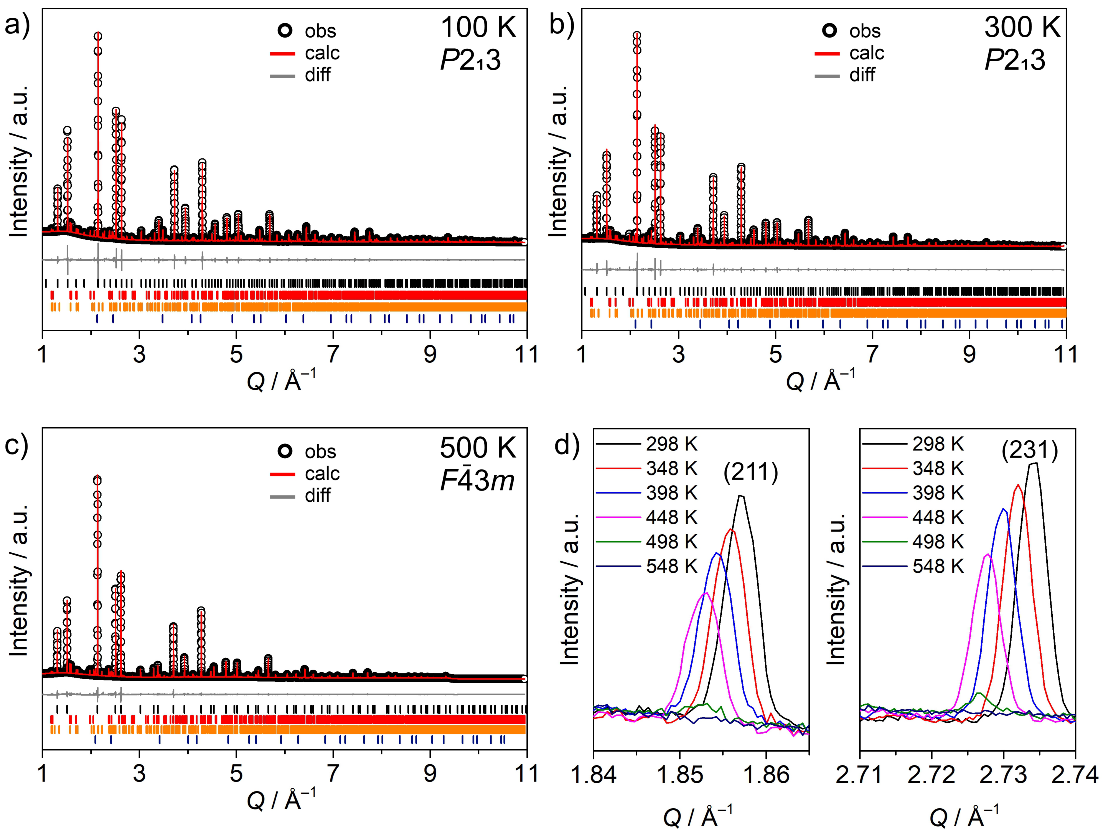

Figure S3: Pawley fits against SXRD data at a)100 K, b) 300 K and c) 500 K for Li_7SiO_5Cl; I_{obs} (black circles), I_{calc} (red line), I_{obs}–I_{calc} (grey line) and Bragg reflections (black tick marks for $Li_{6+x}P_{1-x}Si_xO_5Cl$, red tick marks for Li_4SiO_4, orange tick marks for $Li_6SiO_4Cl_2$ and blue tick marks for LiCl; d) Ordering peaks disappearing as temperature is increased and Li_7SiOCl transitions from $P2_13$ to $F\bar{4}3m$

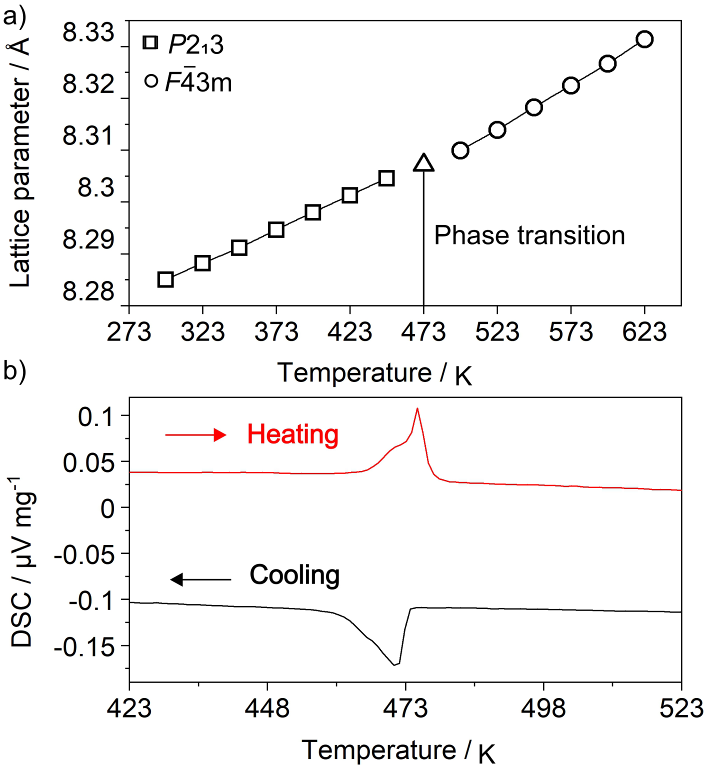

Figure S4: a) Lattice parameters as a function of temperature for Li_7SiO_5Cl b) DSC data collected for Li_7SiO_5Cl, the peak at 472.1(6) K corresponds to the phase transition from $P2_13$ to $F\bar{4}3m$ in Li_7SiO_5Cl, the peak is broadened by the presence of the impurity phase of $Li_6SiO_4Cl_2$ which exhibits a phase transition at a similar temperature.[1]

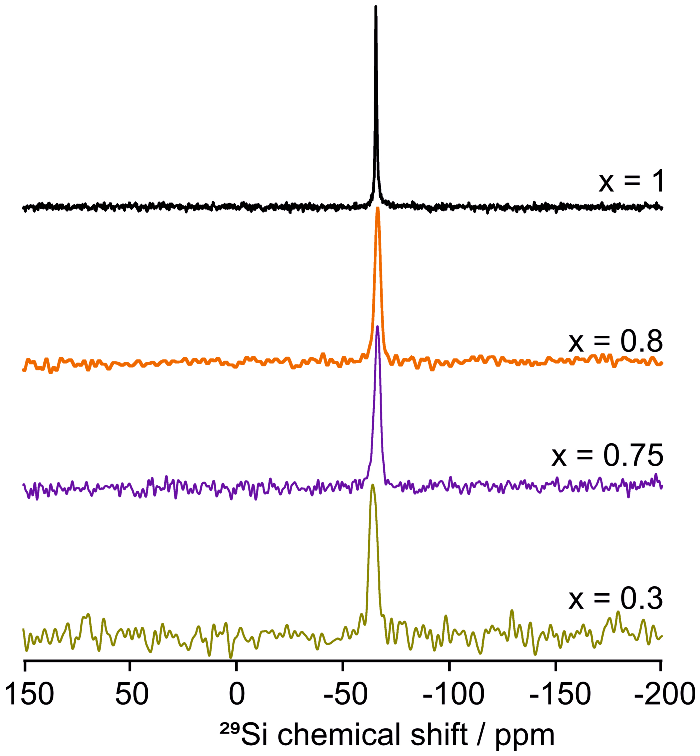

Figure S5: ^{29}Si MAS NMR spectra of $Li_{6+x}P_{1-x}Si_xO_5Cl$ (x = 0.3, 0.75, 0.8 and 1). Full-width half maximum values of 350, 330, 250 and 60 Hz were observed for x = 0.3, 0.75, 0.8 and 1, respectively.

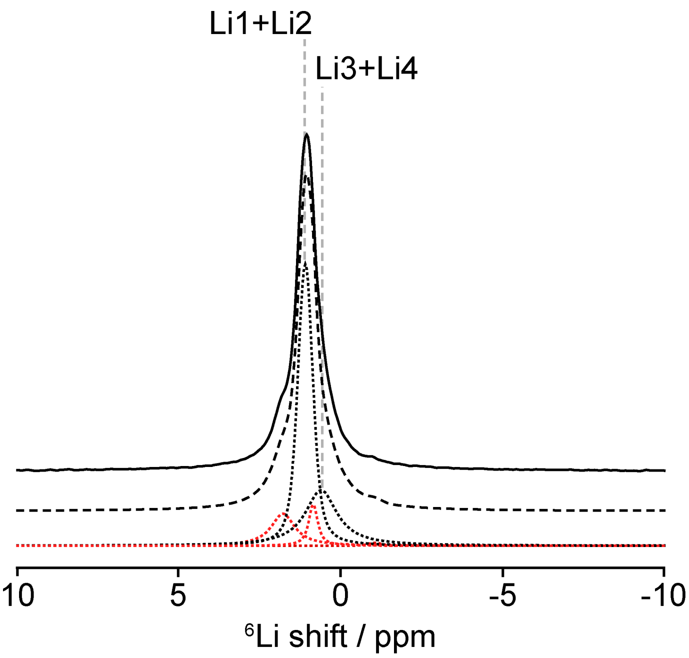

Figure S6. ^{6}Li MAS NMR spectrum of Li_7SiO_5Cl. The experimental spectra (full lines), total fit (dashed lines) and spectral deconvolution (dotted lines) of the ordered Li1 (T5a) and Li2 (T5) as well as the mixed occupancy Li3 (T3) and Li4 (T4) environments. The resonances associated with the largest Li_4SiO_4 impurity (~10 mol%) at 2, 1.2, and -0.7 ppm (red dotted lines)[2] as observed in XRD are also shown while the other impurities are below the NMR detection limit or unresolved due to the small shift range of ^{6}Li NMR.

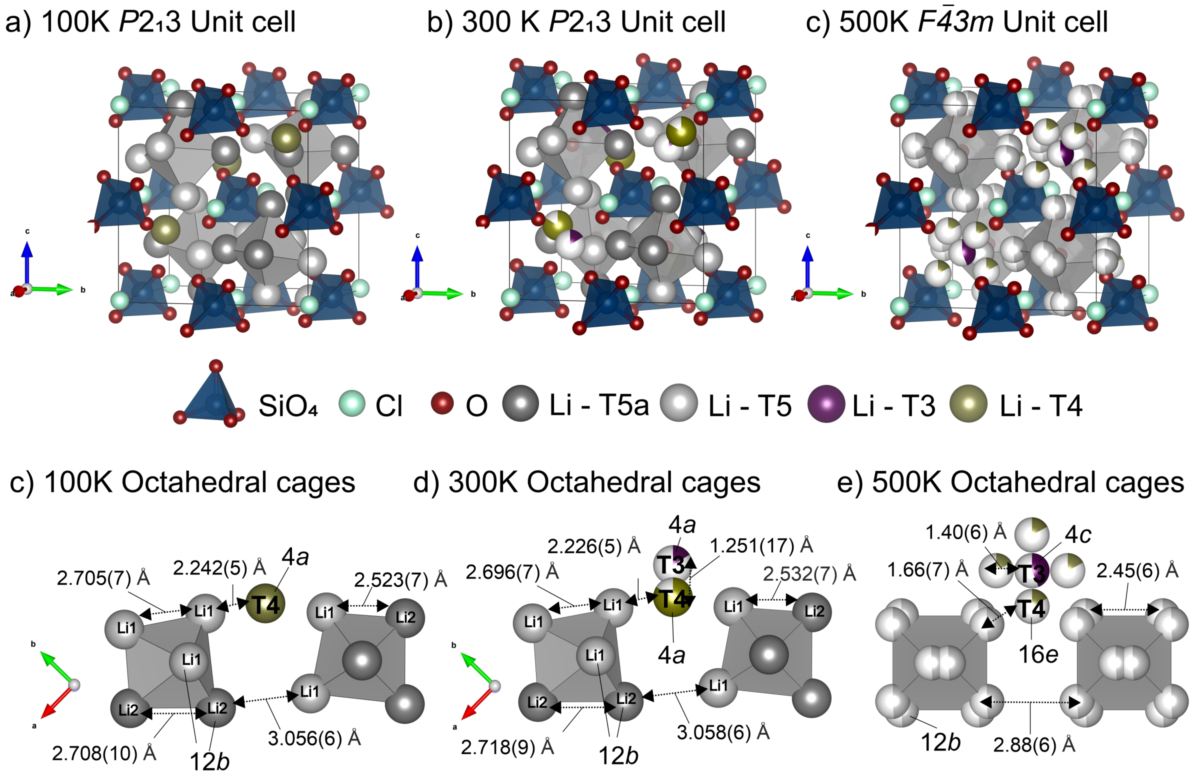

Figure S7: Crystal structure of Li_7SiO_5Cl at 100, 300 and 500 K. Atom and polyhedra colours: SiO_4 tetrahedra (dark blue), O (red), Cl (light blue), Li – T5 site (grey), Li – T3 site (purple), Li – T4 site (green), Vacant sites (white); Unit cell at a) 100 K, b) 300 K, c) 500 K; Octahedral Li ion cages and Li–Li distances at d) 100 K, e) 300 K, and f) 500 K

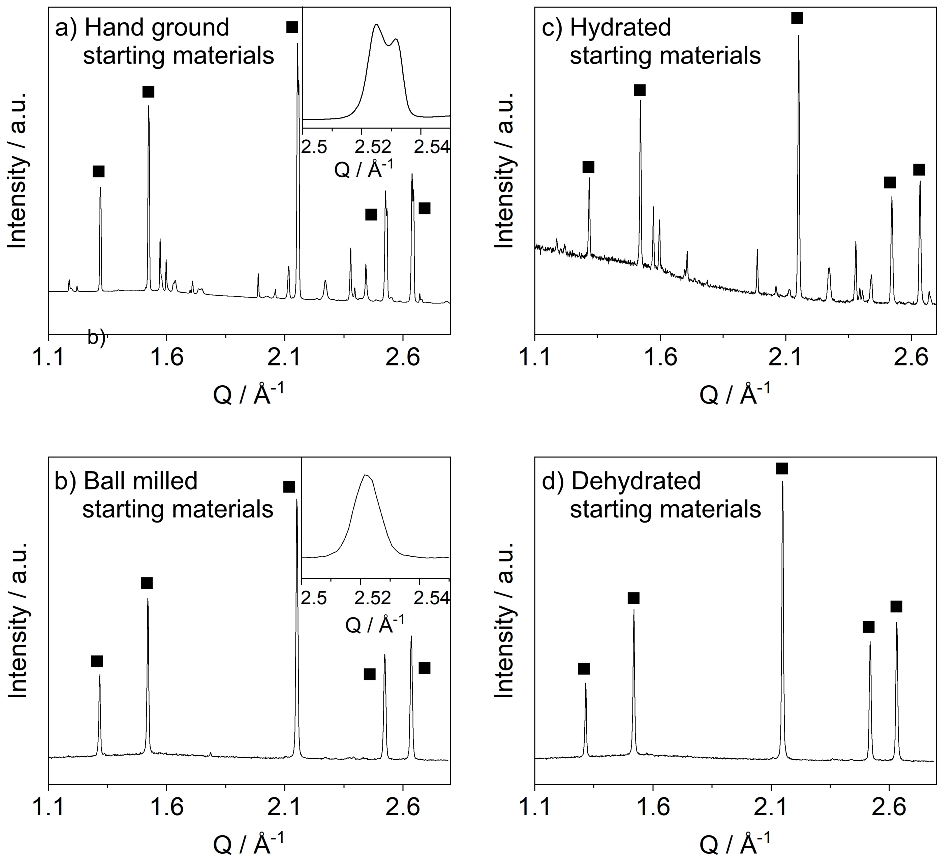

Figure S8: XRD patterns obtained when starting materials are a) hand ground and b) ball milled during the synthesis of $Li_{6.3}P_{0.7}Si_{0.3}O_5Cl$. Black squares denote peaks corresponding to the cubic argyrodite phase. Insets highlight the asymmetric peak shape in hand ground samples due to the presence of several distinct cubic argyrodite phases. When the starting materials are ball milled, a single argyrodite phase is formed as indicated by the symmetric peak shape. XRD patterns obtained when using c) hydrated and d) dehydrated Li_4SiO_4, Li_3PO_4, Li_2O and LiCl starting materials.

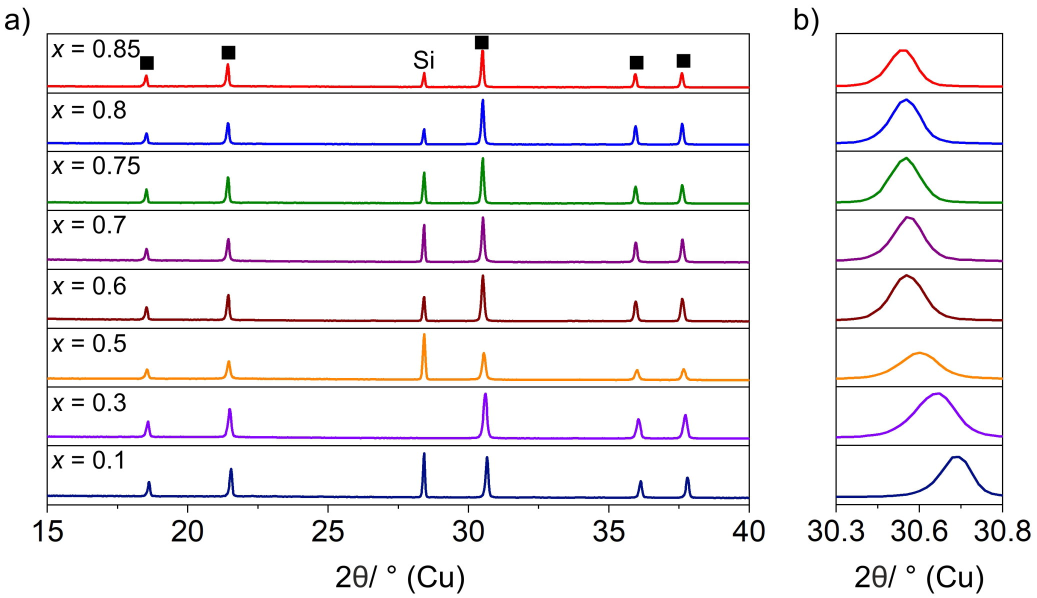

Figure S9: a) XRD patterns for $Li_{6+x}P_{1-x}Si_xO_5Cl$. Black squares denote peaks corresponding to the cubic argyrodite phase and peaks corresponding to Si used as an internal standard are also present in some samples. b) Magnified views of the XRD patterns to highlight peak shift confirming the incorporation of Si and Li into the material.

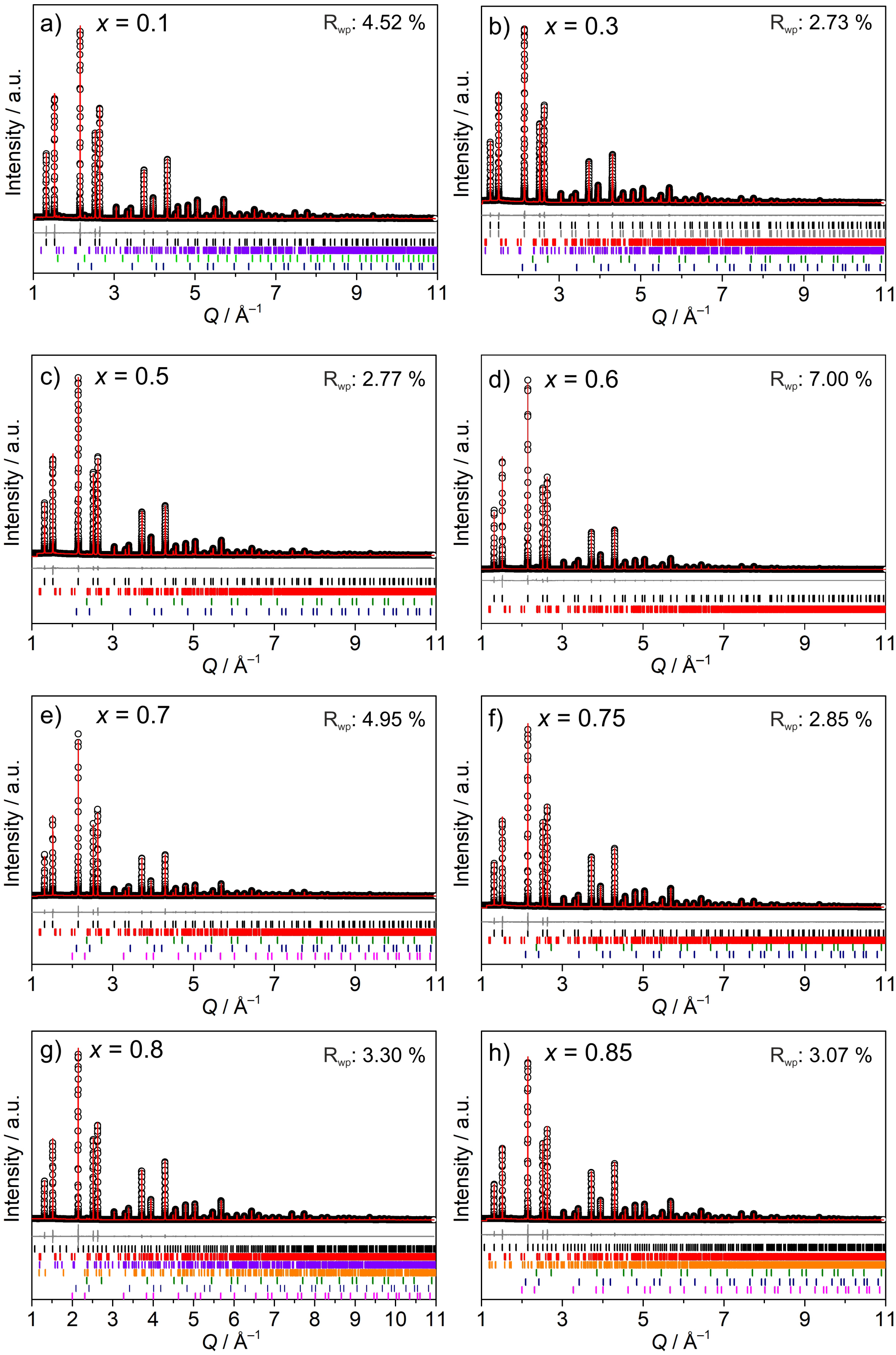

Figure S10: a) Pawley fit against SXRD data of $Li_{6+x}P_{1-x}Si_xO_5Cl$ (Diamond Light Source I11 beam line) with I_{obs} (black circles), I_{calc} (red line), $I_{obs}–I_{calc}$ (grey line) and Bragg reflections (black tick marks for $Li_{6+x}P_{1-x}Si_xO_5Cl$, red tick marks for Li_4SiO_4, purple tick marks for Li_3PO_4, light green tick marks for Li_3OCl, blue tick marks for LiCl, green tick marks for Li_2O, pink tick marks for ZrO_2 and orange tick marks for $Li_6SiO_4Cl_2$); a) $x = 0.1$; b) $x = 0.3$; c) $x = 0.5$; d) $x = 0.6$; e) $x = 0.7$; f) $x = 0.75$; g) $x = 0.8$; h) $x = 0.85$.

Table S6: Empirical formula determined from ICP and SEM-EDX compositional analysis confirming the incorporation of Si and P into the lattice and the increase of Li content with increasing x in $Li_{6+x}P_{1-x}Si_xO_5Cl$

**x in $Li_{6+x}P_{1-x}Si_xO_5Cl$**	**ICP**	**SEM - EDX**	**Empirical formula**
0.1	/	$Si_{0.16(2)}P_{0.84(3)}Cl_{1.04(13)}$	/
0.3	$Li_{6.42(5)}Si_{0.3233(1)}P_{0.6767(3)}$	$Si_{0.36(4)}P_{0.63(3)}Cl_{1.11(2)}$	$Li_{6.42(5)}Si_{0.3233(1)}P_{0.6767(3)}Cl_{1.11(2)}$
0.5	$Li_{6.57(2)}Si_{0.5138(3)}P_{0.4862(5)}$	$Si_{0.55(3)}P_{0.45(1)}Cl_{0.88(3)}$	$Li_{6.57(2)}Si_{0.5138(3)}P_{0.4862(5)}Cl_{0.88(3)}$
0.6	$Li_{6.66(2)}Si_{0.6176(3)}P_{0.3824(2)}$	$Si_{0.66(4)}$ $P_{0.34(3)}Cl_{1.05(1)}$	$Li_{6.66(2)}Si_{0.6176(3)}P_{0.3824(2)}Cl_{1.05(1)}$
0.7	$Li_{6.79(2)}Si_{0.6816(2)}P_{0.3184(3)}$	$Si_{0.72(5)}P_{0.27(3)}Cl_{0.98(6)}$	$Li_{6.79(2)}Si_{0.6816(2)}P_{0.3184(3)}Cl_{0.98(6)}$
0.75	$Li_{6.84(2)}Si_{0.7665(2)}P_{0.2335(5)}$	$Si_{0.79(5)}P_{0.21(3)}Cl_{0.96(6)}$	$Li_{6.84(2)}Si_{0.7665(2)}P_{0.2335(5)}Cl_{0.96(6)}$
0.8	$Li_{6.89(4)}Si_{0.8064(4)}P_{0.1935(3)}$	$Si_{0.82(4)}P_{0.17(2)}Cl_{0.85(6)}$	$Li_{6.89(4)}Si_{0.8064(4)}P_{0.1935(3)}Cl_{0.85(6)}$
0.85	$Li_{6.91(4)}Si_{0.8642(4)}P_{0.1358(3)}$	$Si_{0.88(4)}P_{0.12(1)}Cl_{0.92(8)}$	$Li_{6.91(4)}Si_{0.8642(4)}P_{0.1358(3)}Cl_{0.92(8)}$

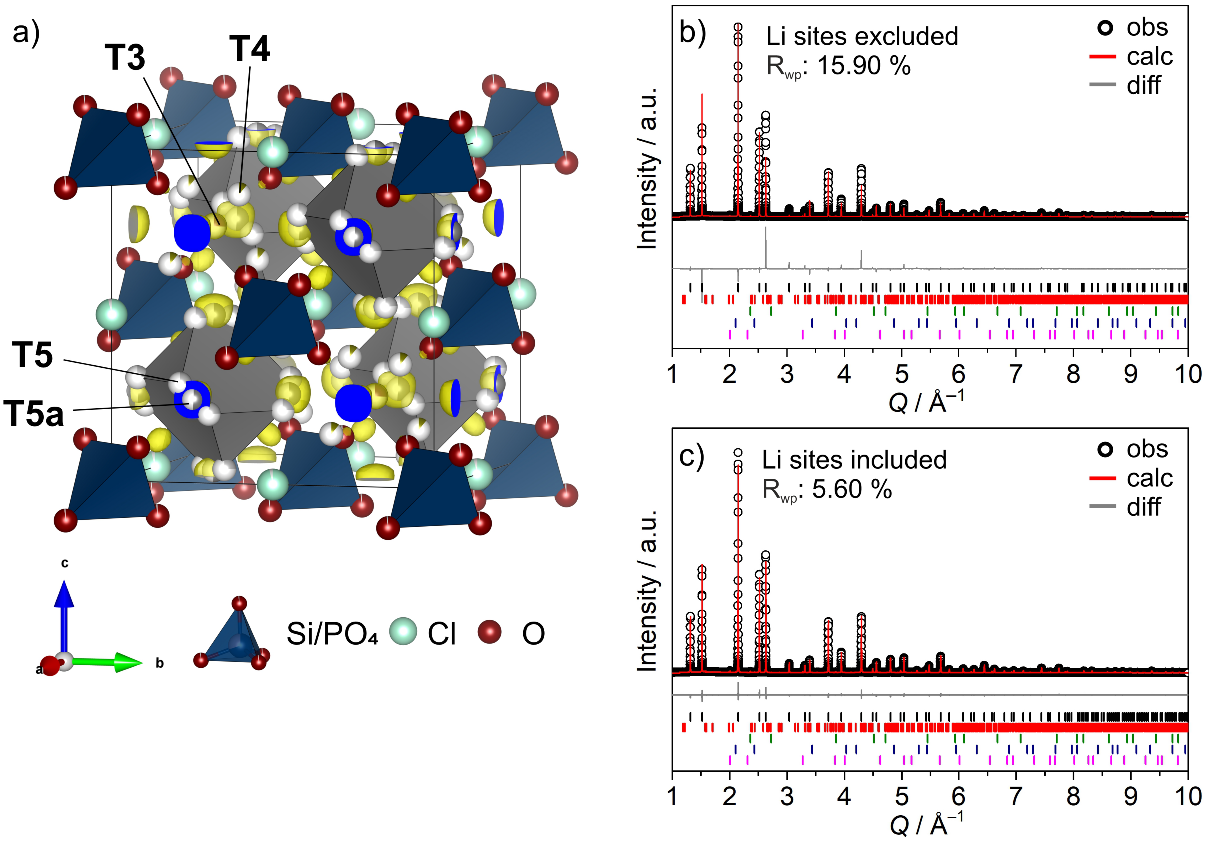

Figure S11A: a) Fourier Difference map for x = 0.7 when the Li sites are not included in the model. Li atoms contribute ~ 20% to the total electron count and additional electron density is clearly visible surrounding the T5, T5a and T3 positions, b) x = 0.7: Rietveld fit against high resolution data excluding all Li^+ sites compared to c) which includes all identified Li^+ sites in the model, highlighting the improvement in visual fit quality and R_{wp} from 15.90 % (excluding Li^+ sites) to 5.60 % (including Li^+ sites); (black tick marks for $Li_{6.7}P_{0.3}Si_{0.7}O_5Cl$, red tick marks for Li_4SiO_4, blue tick marks for LiCl, green tick marks for Li_2O and pink tick marks for ZrO_2).

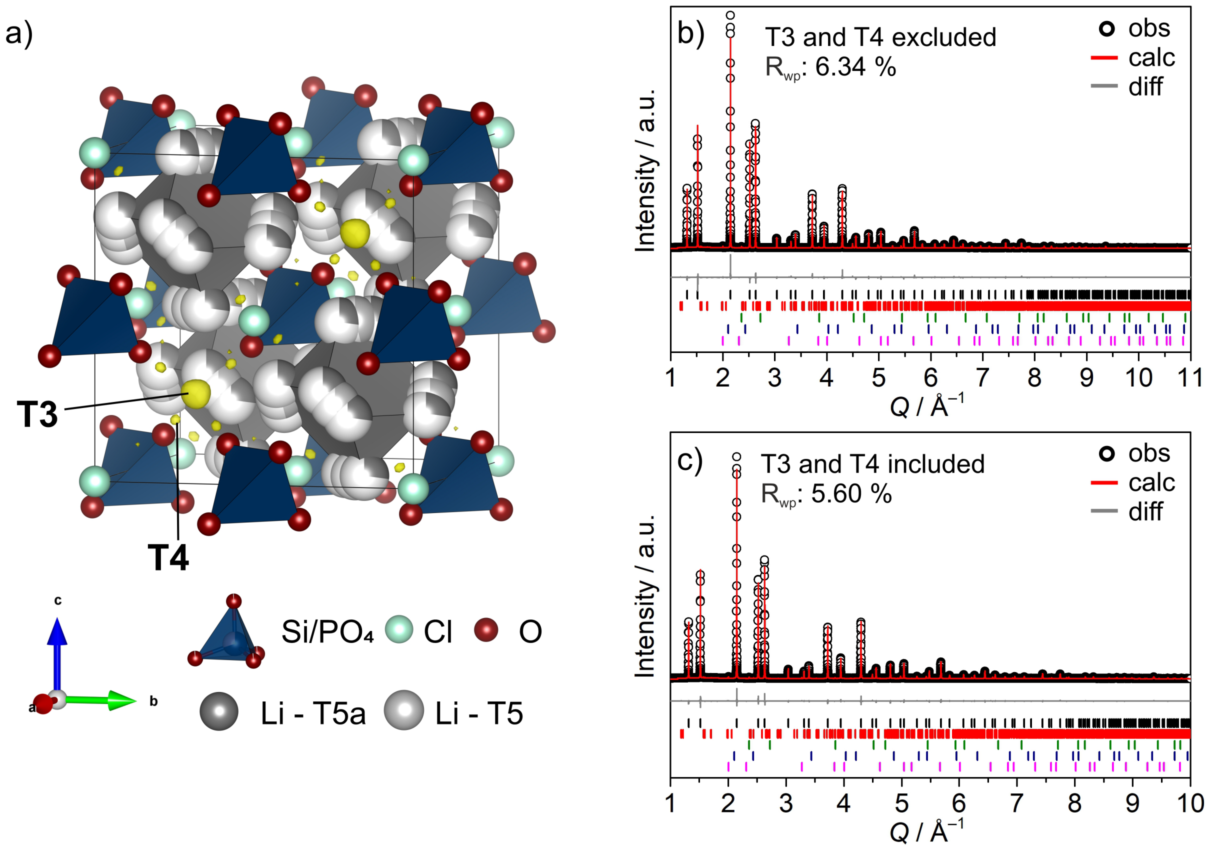

Figure S11B: a) Fourier Difference map for $x = 0.7$ highlighting additional electron density surrounding the T3 and T4 positions when these sites are not included in the model. b) $x = 0.7$: Rietveld fit against high resolution data excluding T3 and T4 sites compared to c) including the T3 and T4 sites in the model, highlighting the improvement in visual fit quality and R_{wp} (black tick marks for $Li_{6.7}P_{0.3}Si_{0.7}O_5Cl$, red tick marks for Li_4SiO_4, blue tick marks for LiCl, green tick marks for Li_2O and pink tick marks for ZrO_2).

Table S7: Structure refinement against powder diffraction data for $Li_{6+x}P_{1-x}Si_xO_5Cl$

Phase	***x* = 0.1**	***x* = 0.3**	***x* = 0.5**	***x* = 0.6**
Empirical Formula	$Li_{6.10(3)}P_{0.90}Si_{0.10}O_{4.9999(14)}Cl_{1.000(2)}$	$Li_{6.200(16)}P_{0.70}Si_{0.30}O_{4.934(15)}Cl_{1.000(2)}$	$Li_{6.430(18)}P_{0.49}Si_{0.51}O_{4.964(3)}Cl_{0.9999(5)}$	$Li_{6.62(2)}P_{0.62}Si_{0.38}O_{4.999(5)}Cl_{0.999(7)}$
Formula Weight (g mol^{-1})	188.47	189.28	190.09	190.50
Space group	$F\bar{4}3m$	$F\bar{4}3m$	$F\bar{4}3m$	$F\bar{4}3m$
Z	4	4	4	4
Density (g cm^{-3})	1.350	1.349	1.347	1.345
Temperature (K)	293.15	293.15	293.15	293.15
Wavelength (Å)	0.826899	0.826899	0.826899	0.82677
d – spacing range (Å)	0.5716-5.9255	0.5716-5.9255	0.5716-5.9255	0.4298-6.3351
No. of reflections	110	110	110	236
No. of refined parameters	27	35	28	26
a (Å)	8.234301(17)	8.248176(16)	8.264610(15)	8.273920(16)
Volume (Å)	558.316(3)	561.143(3)	564.504(3)	566.414(3)
R_p	3.84	2.54	2.46	5.56
R_{wp}	5.80	3.48	3.34	8.13
R_{exp}	0.85	0.75	0.87	1.70
χ^2	46.64	21.53	14.74	22.86
**Phase**	***x* = 0.7**	***x* = 0.75**	***x* = 0.8**	***x* = 0.85**
Empirical Formula	$Li_{6.660(17)}P_{0.68}Si_{0.32}O_{4.983(3)}Cl_{0.9886(5)}$	$Li_{6.69(2)}P_{0.75}Si_{0.25}O_{4.926(3)}Cl_{0.9764(7)}$	$Li_{6.78(5)}P_{0.80}Si_{0.20}O_{4.995(11)}Cl_{0.9902(5)}$	$Li_{6.85(2)}P_{0.85}Si_{0.15}O_{4.987(7)}Cl_{0.982785)}$
Formula Weight (g mol^{-1})	190.90	191.105	191.308	191.511
Space group	$F\bar{4}3m$	$F\bar{4}3m$	$P2_13$	$P2_13$
Z	4	4	4	4
Density (g cm^{-3})	1.349	1.349	1.350	1.347
Temperature (K)	293.15	293.15	293.15	293.15
Wavelength (Å)	0.82677	0.826899	0.826899	0.826899
d – spacing range (Å)	0.4298-6.3351	0.5716-5.9255	0.5716-5.9255	0.5716-5.9255
No. of reflections	236	110	627	627
No. of refined parameters	28	32	46	43
a (Å)	8.271684(9)	8.275968(9)	8.276883(9)	8.278181(4)
Volume (Å)	565.955(2)	566.835(2)	567.023(3)	567.290(1)
R_p	4.38	2.37	2.61	2.51
R_{wp}	5.60	3.27	3.79	3.55
R_{exp}	2.07	0.90	1.04	1.07
χ^2	7.32	13.20	13.28	11.00

Table S8: Atomic positions, isotropic atomic displacement parameters (U_{iso}, Å^2×10^3), and site occupancy factors for $Li_{6+x}P_{1-x}Si_xO_5Cl$ obtained from refinement against powder diffraction data.

**Site**	**Wyckoff position**	**x**	**y**	**z**	**s.o.f.**	**U_{iso} (Å^2×10^3)**
			**$Li_{6.1}P_{0.1}Si_{0.9}O_5Cl$**			
Cl1	4*a*	0	0	0	1.000(2)	1.232(7)
Si1	4*b*	0.5	0.5	0.5	0.1	0.18(6)
P1	4*b*	0.5	0.5	0.5	0.9	0.528(7)
O1	16*e*	0.38962(5)	0.38962(5)	0.38962(5)	1.000(3)	0.292(9)
O2	4*d*	0.75	0.75	0.75	1.000(3)	0.470(15)
Li1	24*g*	0.51580(3)	0.25	0.25	0.841(17)	1.58(2)
Li1a	48*h*	0.700(2)	0.700(2)	0.986(3)	0.080(9)	1.0(4)
Li2	4*c*	0.25	0.25	0.25	0.1	5.00
			**$Li_{6.3}P_{0.7}Si_{0.3}O_5Cl$**			
Cl1	4*a*	0	0	0	1.000(2)	1.351(6)
Si1	4*b*	0.5	0.5	0.5	0.3	1.81(2)
P1	4*b*	0.5	0.5	0.5	0.7	0.103(5)
O1	16*e*	0.38862(4)	0.38862(4)	0.38862(4)	0.9837(4)	0.610(7)
O2	4*d*	0.75	0.75	0.75	1.000(2)	0.55(1)
Li1	24*g*	0.5169(5)	0.25	0.25	0.521(1)	0.61(2)
Li1a	48*h*	0.7123(9)	0.7123(9)	0.9814(7)	0.2353(6)	0.70(5)
Li2	4*c*	0.25	0.25	0.25	0.070(5)	4.6(8)
Li3	16*e*	0.163(4)	0.163(4)	0.163(4)	0.043(2)	2.5(6)
			**$Li_{6.5}P_{0.5}Si_{0.5}O_5Cl$**			
Cl1	4*a*	0	0	0	0.9999(5)	1.402(8)
Si1	4*b*	0.5	0.5	0.5	0.51	1.14(7)
P1	4*b*	0.5	0.5	0.5	0.49	0.33(5)
O1	16*e*	0.38770(3)	0.38770(3)	0.38770(3)	0.9911(5)	0.806(13)
O2	4*d*	0.75	0.75	0.75	1.0000(9)	0.61(2)
Li1	24*g*	0.5159(4)	0.25	0.25	0.5125(9)	0.88(8)
Li1a	48*h*	0.7092(7)	0.7092(7)	0.9789(5)	0.2369(6)	0.81(1)
Li2	4*c*	0.25	0.25	0.25	0.159(3)	1.0(5)
Li3	16*e*	0.1507(15)	0.1507(15)	0.1507(15)	0.0883(7)	3.0(6)
			**$Li_{6.6}P_{0.4}Si_{0.6}O_5Cl$**			
Cl1	4*a*	0	0	0	0.9999(7)	1.678(11)
Si1	4*b*	0.5	0.5	0.5	0.62	0.9(4)
P1	4*b*	0.5	0.5	0.5	0.38	1.0(6)
O1	16*e*	0.38810(5)	0.38810(5)	0.38810(5)	0.9999(9)	1.029(15)
O2	4*d*	0.75	0.75	0.75	1.000(15)	0.91(3)
Li1	24*g*	0.5146(7)	0.25	0.25	0.4561(14)	1.18(18)
Li1a	48*h*	0.7110(16)	0.7110(16)	0.9829(11)	0.2710(9)	3.3(1)
Li2	4*c*	0.25	0.25	0.25	0.164(4)	1.0(5)
Li3	16*e*	0.1563(9)	0.1563(9)	0.1563(9)	0.1171(11)	0.99(2)
			**$Li_{6.7}P_{0.3}Si_{0.7}O_5Cl$**			
Cl1	4*a*	0	0	0	0.9886(5)	1.222(5)
Si1	4*b*	0.5	0.5	0.5	0.68	0.538(5)
P1	4*b*	0.5	0.5	0.5	0.32	2.18(2)
O1	16*e*	0.38743(3)	0.38743(3)	0.38743(3)	0.9965(6)	0.927(9)
O2	4*d*	0.75	0.75	0.75	0.9968(10)	0.871(17)
Li1	24*g*	0.5166(3)	0.25	0.25	0.5125(10)	1.00(7)

Li1a	48*h*	0.7046(7)	0.7046(7)	0.9754(5)	0.2437(7)	2.1(1)
Li2	4*c*	0.25	0.25	0.25	0.284(3)	0.61(17)
Li3	16*e*	0.1420(17)	0.1420(17)	0.1420(17)	0.090(3)	4.5(7)
			**$Li_{6.75}P_{0.25}Si_{0.75}O_5Cl$**			
Cl1	4*a*	0	0	0	0.976(2)	1.357(6)
Si1	4*b*	0.5	0.5	0.5	0.75	0.12(1)
P1	4*b*	0.5	0.5	0.5	0.25	3.2(1)
O1	16*e*	0.38744(3)	0.38744(3)	0.38744(3)	0.982(3)	0.84(1)
O2	4*d*	0.75	0.75	0.75	0.9968(19)	0.70(2)
Li1	24*g*	0.5155(3)	0.25	0.25	0.508(15)	0.99(7)
Li1a	48*h*	0.7066(2)	0.7066(2)	0.9733(5)	0.2412(11)	1.74(9)
Li2	4*c*	0.25	0.25	0.25	0.209(2)	0.23(2)
Li3	16*e*	0.1510(9)	0.1510(9)	0.1510(9)	0.1345(7)	3.3(4)
			**$Li_{6.8}P_{0.2}Si_{0.8}O_5Cl$**			
Cl1	4*a*	0.0011(3)	0.0011(3)	0.0011(3)	0.9901(5)	1.430(7)
Si1	4*a*	0.4989(3)	0.4989(3)	0.4989(3)	0.80	0.50(11)
P1	4*a*	0.4989(3)	0.4989(3)	0.4989(3)	0.20	1.0(5)
O1	4*a*	0.6119(5)	0.6119(5)	0.6119(5)	0.999(9)	1.45(15)
O2	12*b*	0.3842(4)	0.3878(5)	0.6108(5)	0.9990(4)	0.73(3)
O3	4*a*	0.2477(8)	0.2477(8)	0.2477(8)	0.998(1)	0.668(3)
Li1	12*b*	0.01331	0.25000	0.25000	0.5111(17)	1.0(6)
Li1a	12*b*	0.20757	0.20757	0.02923	0.218(2)	0.98(1)
Li1b	12*b*	0.79243	0.20757	0.97077	0.267(2)	2.1(3)
Li2	12*b*	0.48669	0.25000	0.25000	0.4773(18)	1.0(6)
Li2a	12*b*	0.79243	0.79243	0.02923	0.177(3)	1.4(3)
Li2b	12*b*	0.20757	0.79243	0.97077	0.344(2)	1.2(3)
Li3	4*a*	0.7561(14)	0.7561(14)	0.7561(14)	0.224(8)	0.4(2)
Li3a	4*a*	0.8455(11)	0.8455(11)	0.8455(11)	0.23(2)	1.7(2)
Li3b	12*b*	0.150(2)	0.152(2)	0.847(2)	0.114(8)	1.2(2)
			**$Li_{6.85}P_{0.15}Si_{0.85}O_5Cl$**			
Cl1	4*a*	0.00244(6)	0.00244(6)	0.00244(6)	0.9829(4)	1.349(4)
Si1	4*a*	0.49841(6)	0.49841(6)	0.49841(6)	0.85	0.43(3)
P1	4*a*	0.49841(6)	0.49841(6)	0.49841(6)	0.15	1.3(2)
O1	4*a*	0.6107(2)	0.6107(2)	0.6107(2)	0.999(2)	1.62(6)
O2	12*b*	0.38332(17)	0.38332(17)	0.38332(17)	1.000(3)	0.61(1)
O3	4*a*	0.2487(3)	0.2487(3)	0.2487(3)	0.9939(9)	0.675(9)
Li1	12*b*	0.01331	0.25000	0.25000	0.4874(16)	0.96(3)
Li1a	12*b*	0.20757	0.20757	0.02923	0.213(2)	1.2(2)
Li1b	12*b*	0.79243	0.20757	0.97077	0.291(2)	2.3(2)
Li2	12*b*	0.48669	0.25000	0.25000	0.4696(16)	1.00(3)
Li2a	12*b*	0.79243	0.79243	0.02923	0.176(2)	1.5(2)
Li2b	12*b*	0.20757	0.79243	0.97077	0.355(2)	1.16(12)
Li3	4*a*	0.7544(16)	0.7544(16)	0.7544(16)	0.240(3)	0.51(9)
Li3a	4*a*	0.8456(7)	0.8456(7)	0.8456(7)	0.373(16)	2.0(4)
Li3b	12*b*	0.153(3)	0.154(3)	0.847(3)	0.081(6)	0.56(17)

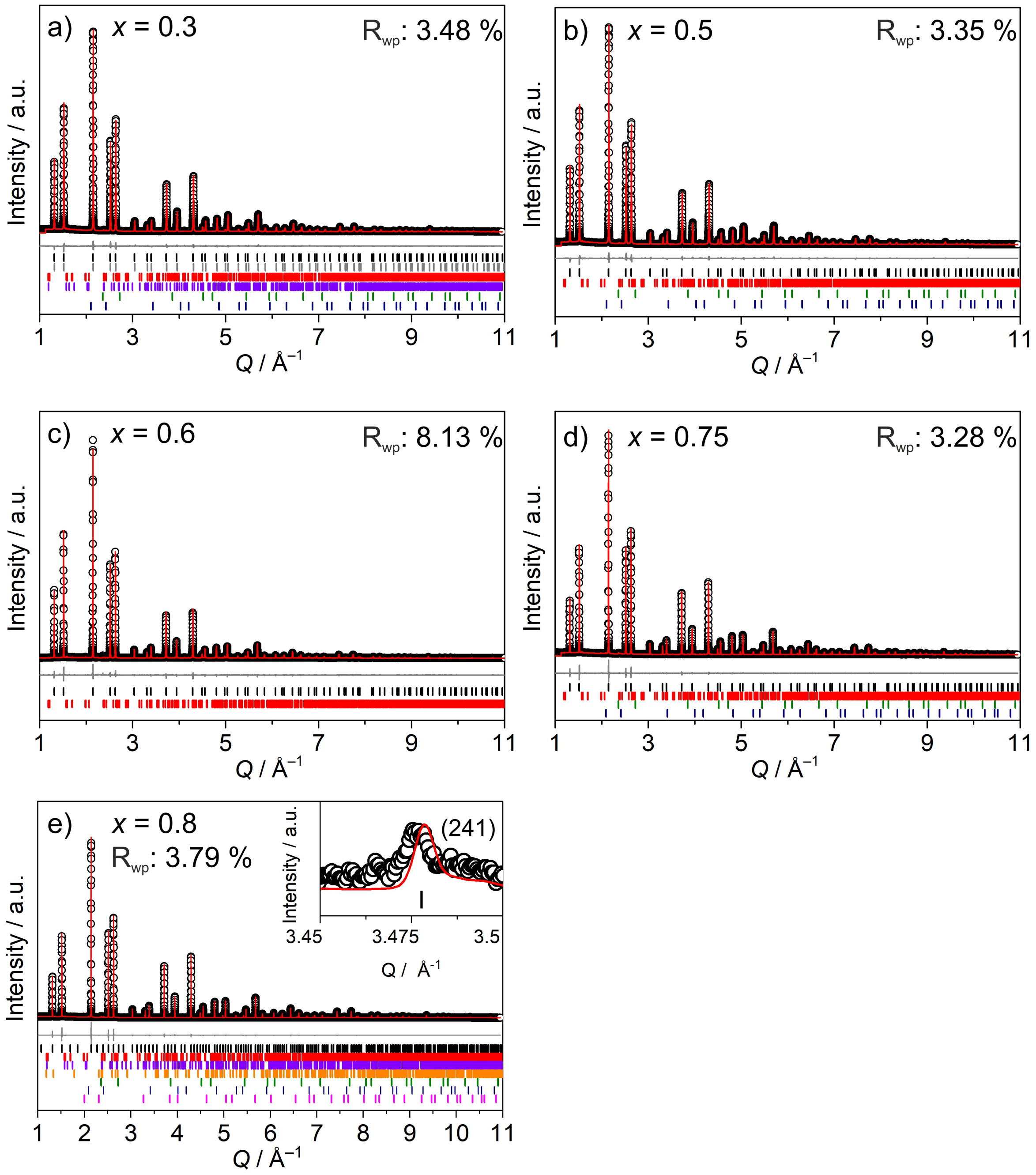

Figure S12: a) Rietveld refinements against SXRD of $Li_{6+x}P_{1-x}Si_xO_5Cl$ (Diamond Light Source I11 beam line) with I_{obs} (black circles), I_{calc} (red line), I_{obs}–I_{calc} (grey line) and Bragg reflections (black tick marks for $Li_{6+x}P_{1-x}Si_xO_5Cl$, red tick marks for Li_4SiO_4, purple tick marks for Li_3PO_4, light green tick marks for Li_3OCl, blue tick marks for LiCl, green tick marks for Li_2O, pink tick marks for ZrO_2 and orange tick marks for $Li_6SiO_4Cl_2$); a) $x = 0.3$; b) $x = 0.5$; c) $x = 0.6$; d) $x = 0.75$; e) $x = 0.8$, inset highlights one of the supercell reflections (241)

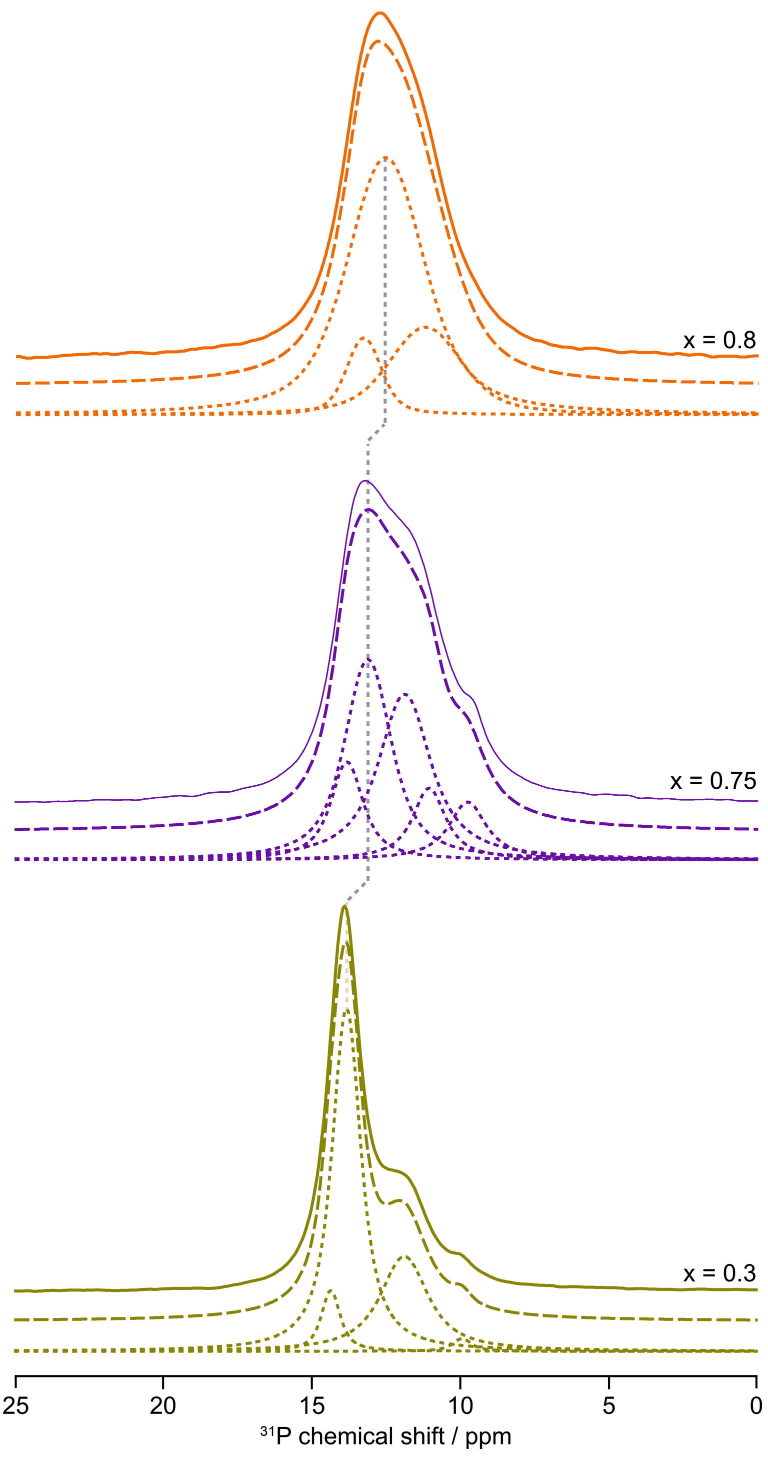

Figure S13: ^{31}P MAS NMR spectra of $Li_{6+x}P_{1-x}Si_xO_5Cl$ (x = 0.3, 0.75 and 0.8). The experimental spectra (full lines), total fit (dashed lines) and spectral deconvolution (dotted lines) of the different ^{31}P environments arising from differing second coordination spheres due to the site occupancies of neighbouring Li environments. The percentage contributions of each resonance to the overall line shape is available in Table S8. The vertical dashed lines highlight the decrease in chemical shift of the most intense resonance, arising from the increased statistical probability of a greater number of Li atoms in the ^{31}P second coordination sphere, due to the increasing Li content.

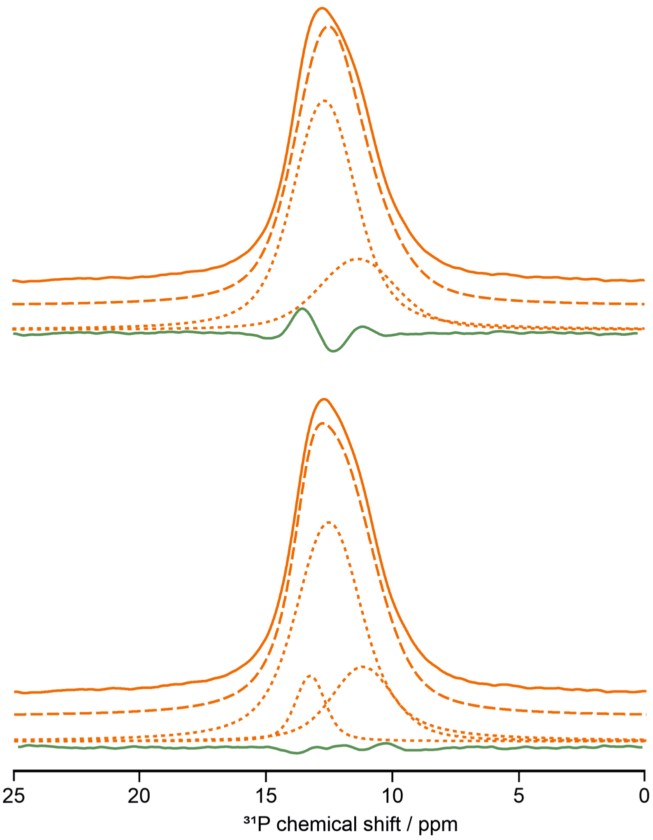

Figure S14: ^{31}P MAS NMR spectra of $Li_{6.8}P_{0.2}Si_{0.8}O_5Cl$ justifying the necessity of a resonance at higher chemical shift (13 ppm) with respect to the main peak (12.5 ppm), where a two-fit model (top) and a three-fit model (bottom) are shown with the residual fits (full green lines). The experimental spectra (full orange lines), total fit (dashed orange lines), spectral deconvolution (dotted orange lines) of the different ^{31}P environments are also shown.

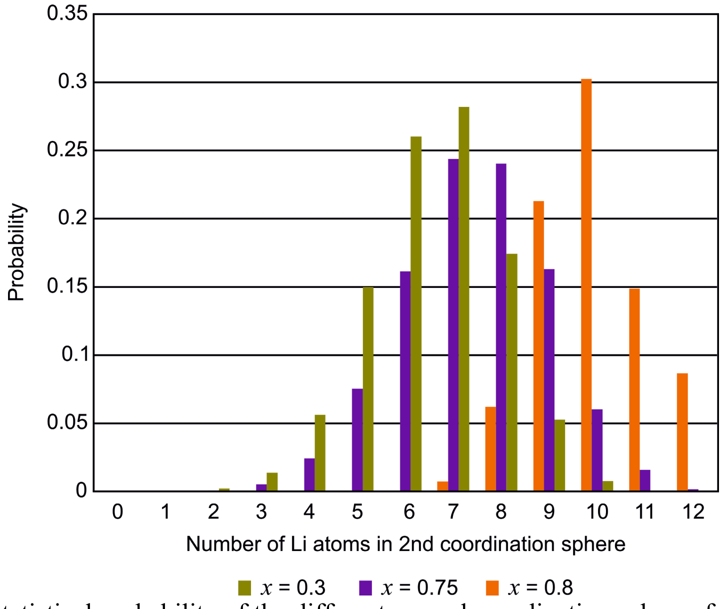

Figure S15: Relative statistical probability of the different second coordination spheres for $Li_{6+x}P_{1-x}Si_xO_5Cl$ (x = 0.3, 0.75 and 0.8) from the statistical probability according to the Li site occupancies observed in refinements against SXRD data (Table S7).

Table S9: Summary of the extracted ^{31}P NMR parameters and the tentative assignments for $Li_{6+x}P_{1-x}Si_xO_5Cl$ (x = 0.3, 0.75 and 0.8) from the statistical probability according to the Li site occupancies observed in refinements against SXRD data (Table S7).

x	**^{31}P Chemical shift / ppm**	**% of overall NMR signal**	**No of Li atoms in 2nd coordination sphere**	**% probability according to XRD**
0.8	13.3 ± 0.8	7 ± 2	7 + 8	7 ± 1
	12.6 ± 1.5	74 ± 7	9 + 10	52 ± 14
	11.3 ± 1.5	20 ± 5	11 + 12	24 ± 6
0.75	13.8 ± 0.6	11 ± 1	4 + 5	11 ± 1
	13.1 ± 0.9	38 ± 5	6 + 7	40 ± 2
	11.9 ± 1	34 ± 5	8 + 9	40 ± 2
	11.0 ± 0.6	8 ± 2	10	6 ± 1
	9.6 ± 0.7	5 ± 1	11 + 12	2 ± 1
0.3	14.3 ± 0.3	6 ± 2	4	6 ± 1
	13.9 ± 0.5	65 ± 3	5 + 6 + 7	69 ± 5
	11.9 ± 0.9	28 ± 3	8 + 9	23 ± 2
	10.0 ± 0.4	1.3 ± 3	10 + 11	1.0 ± 0.1

In order to probe the local phosphorous environments in $Li_{6+x}P_{1-x}Si_xO_5Cl$ (x = 0.3, 0.75 and 0.8), ^{31}P MAS NMR spectra were recorded (Figure S12). For all three compositions, multiple contributions to the overall NMR signal are observed and comprised of 3 to 5 resonances while a single average phosphorous environment is modelled in long-range Rietveld structural refinement. As x increases, individual contributions to the NMR signal become more difficult to resolve. In order to analyse the different components to the overall NMR signal, the spectra were deconvoluted with the fewest number of signals (Figure S13) and the relative area fractions of the distinguishable ^{31}P NMR signals were extracted (Figure S14). This analysis reveals a trend towards lower chemical shift for the most intense ^{31}P resonance and an overall decrease in the intensity of signals appearing at higher chemical shift with increasing x content.

These various NMR resonances arise as the ^{31}P nucleus is sensitive to the second coordination sphere, namely the T3, T4 and T5 Li sites. Hence when the site occupancy of these Li sites is less than 1, a statistical distribution of chemical shifts is observed. For example, the likelihood of a ^{31}P nucleus having 0 to 12 Li atoms in its second coordination environment will differ depending on the Li site occupancy of the varying sites. The second coordination sphere of ^{31}P in the three compositions analysed is complex as for all three phases there can be up to 12 second nearest neighbour Li atoms, 8 × T5 and 4 × T3/T4 Li atoms with varying site occupancies (Figure 6). The trend towards lower chemical shift with increasing x content can be explained through the higher concentration of Li atoms on the T3 and T4 position as this provides an increase in electron density in the second coordination sphere of the ^{31}P nucleus and hence an increase in the chemical shielding.

Moreover, the resonances were compared with the expected ratios of the different possible second nearest neighbour environments for phosphorous using the site occupancies of the different lithium sites obtained from structural refinement against SXRD data (Figure S15) and the NMR resonances assigned from the relationship between coordination number and chemical shift (Table S8). The assignment of the different contributions to the overall NMR line shape agrees reasonably well with the expected statistical probabilities obtained from XRD. For example, in x = 0.75 according to the Rietveld refinement, 4 or 5 Li atoms are expected within the second coordination sphere 11 % of the time while 6 or 7 Li atoms are predicted 40 % of the time, the corresponding resonances in the ^{31}P MAS NMR correspond to 11 and 38 % of the overall signal respectively. However due to the small chemical shift change expected when a single Li atom is added or removed from the second coordination sphere (due to the relatively small shielding effects of Li atoms at this atomic distance), coupled with the overlapping signals corresponding to the various different possible configurations, it was not possible to discriminate between all observable NMR signal contributions.

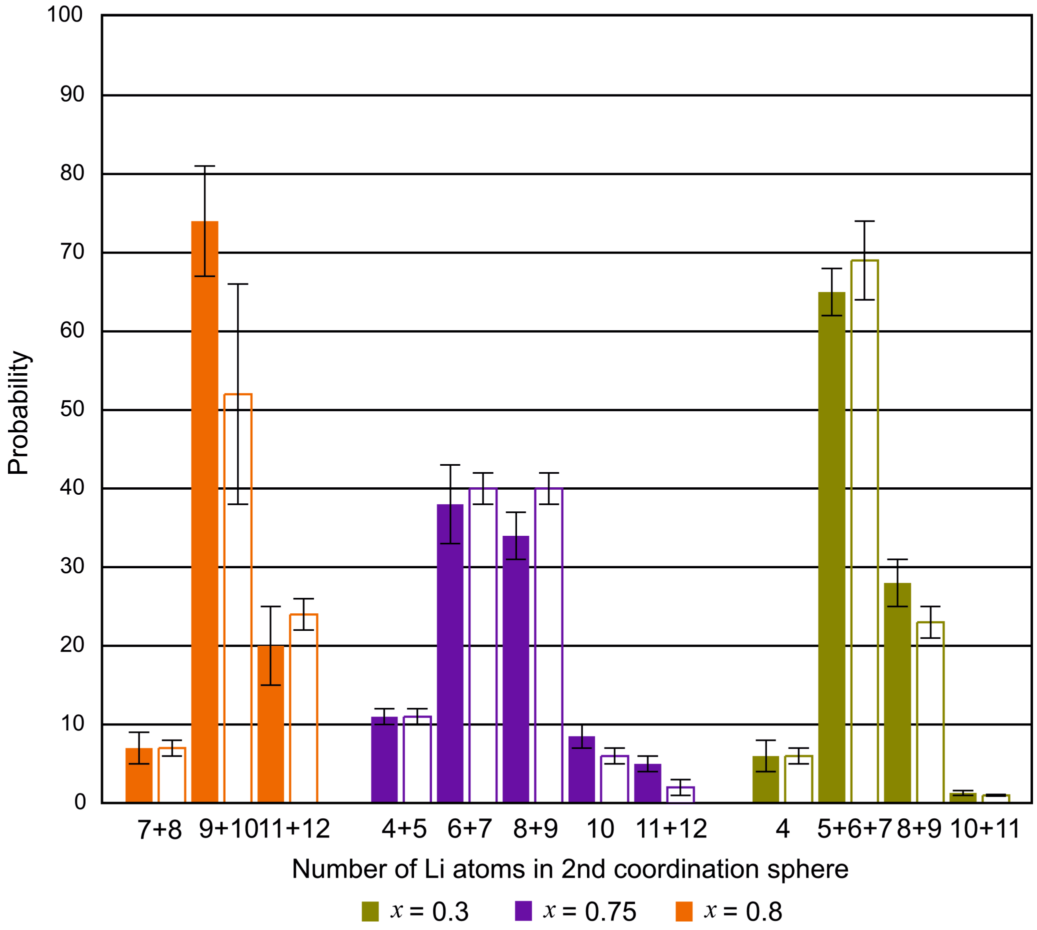

Figure S16: Comparison of percentage probability of the different second coordination spheres for $Li_{6+x}P_{1-x}Si_xO_5Cl$ (x = 0.3, 0.75 and 0.8) from the statistical probability according to the Li site occupancies observed in structural refinement against SXRD data (filled bars) (Table S7) and integrations of the ^{31}P MAS resonances (unfilled bars).

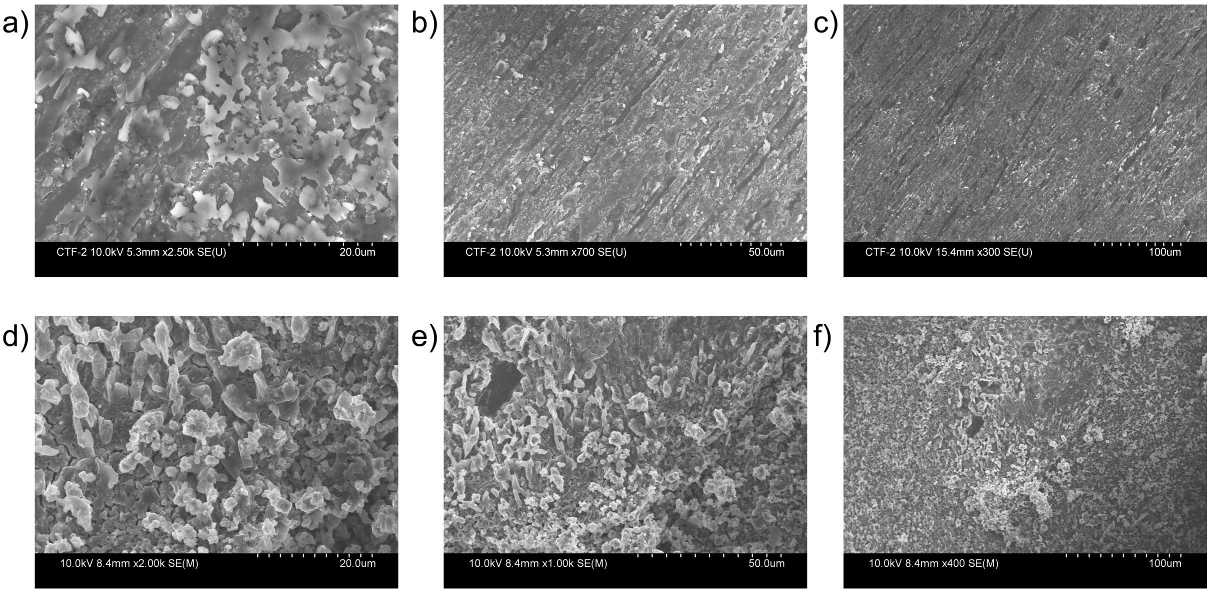

Figure S17: SEM Images of $Li_{6.7}P_{0.3}Si_{0.7}O_5Cl$ pellets prepared *via* SPS (a-c) and reactive sintering (d-f).

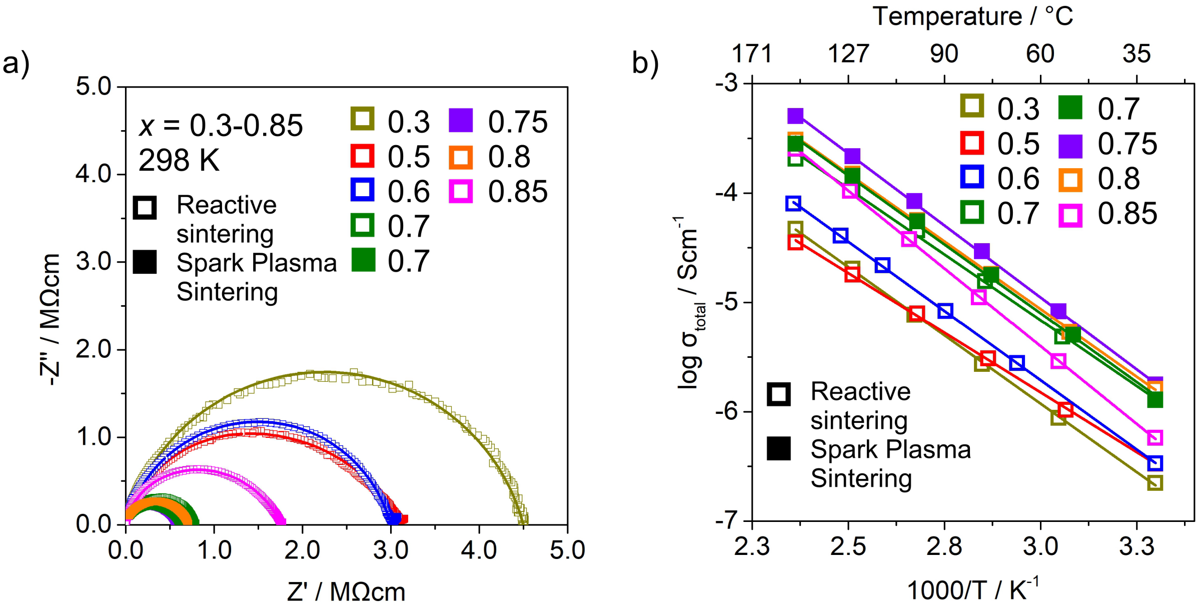

Figure S18: a) Nyquist plots for $Li_{6+x}P_{1-x}Si_xO_5Cl$; samples prepared via reactive sintering (RS) (x = 0.3, 0.5, 0.6, 0.7, 0.75, 0.8, 0.85) and SPS (x = 0.7, 0.75); b) Arrhenius plots for $Li_{6+x}P_{1-x}Si_xO_5Cl$ for samples prepared via reactive sintering (RS) (x = 0.3, 0.5, 0.6, 0.7, 0.8, 0.85) and SPS (x = 0.7, 0.75).

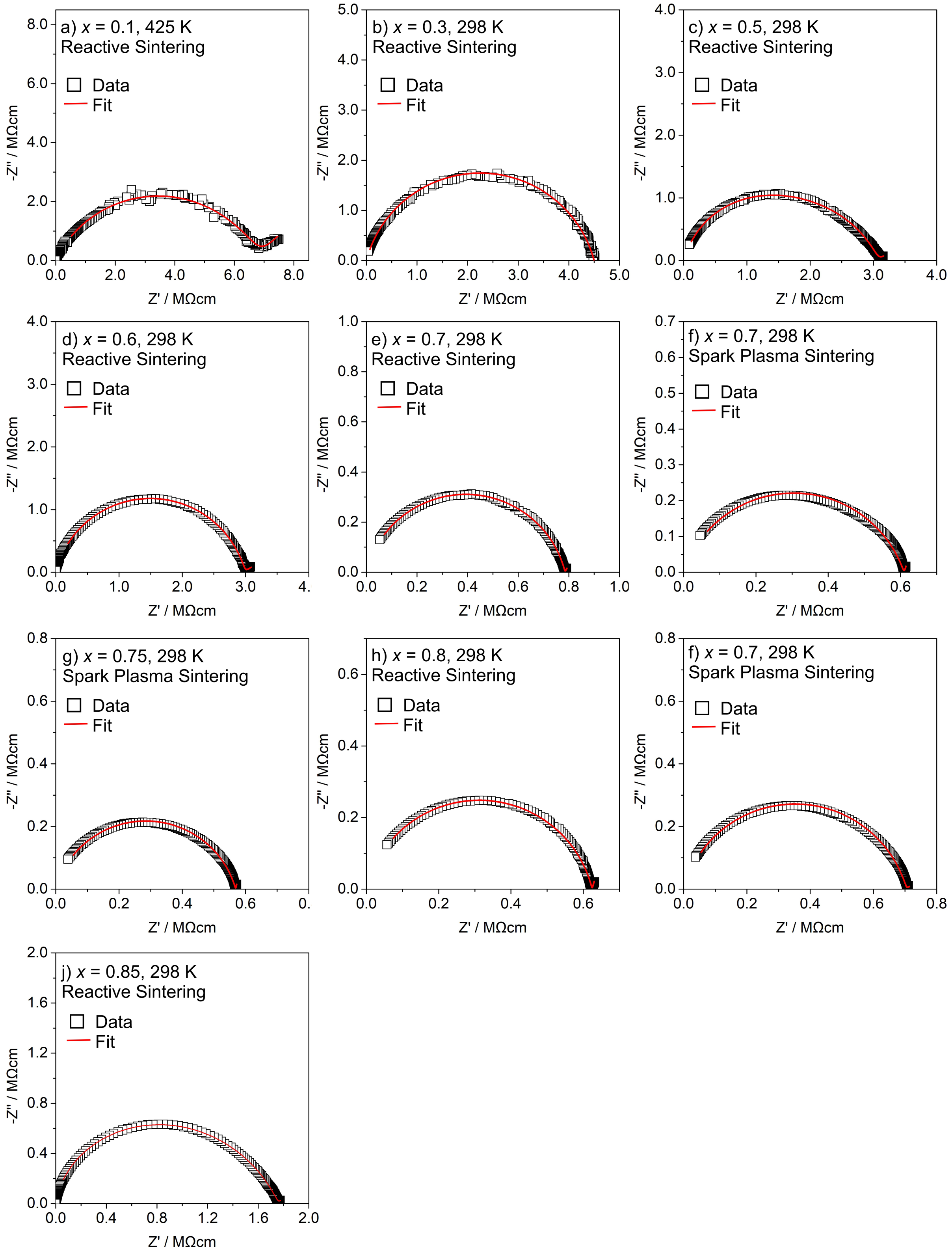

Figure S19: Nyquist plots for $Li_{6+x}P_{1-x}Si_xO_5Cl$; samples prepared via reactive sintering (RS) (x = 0.1, 0.3, 0.5, 0.6, 0.7, 0.8, 0.85) and spark plasma sintering (SPS) (x = 0.7, 0.75, 0.8); a) 0.1 (RS), b) 0.3 (RS), c) 0.5 (RS), d) 0.6 (RS), e) 0.7 (RS), f) 0.7 (SPS) g) 0.75 (SPS), h) 0.8 (RS), i) 0.8 (SPS), j) 0.85(RS); Data for x = 0.3-0.85 was collected at room temperature, due to the low room temperature ionic conductivity of x = 0.1 data was collected at 425 K

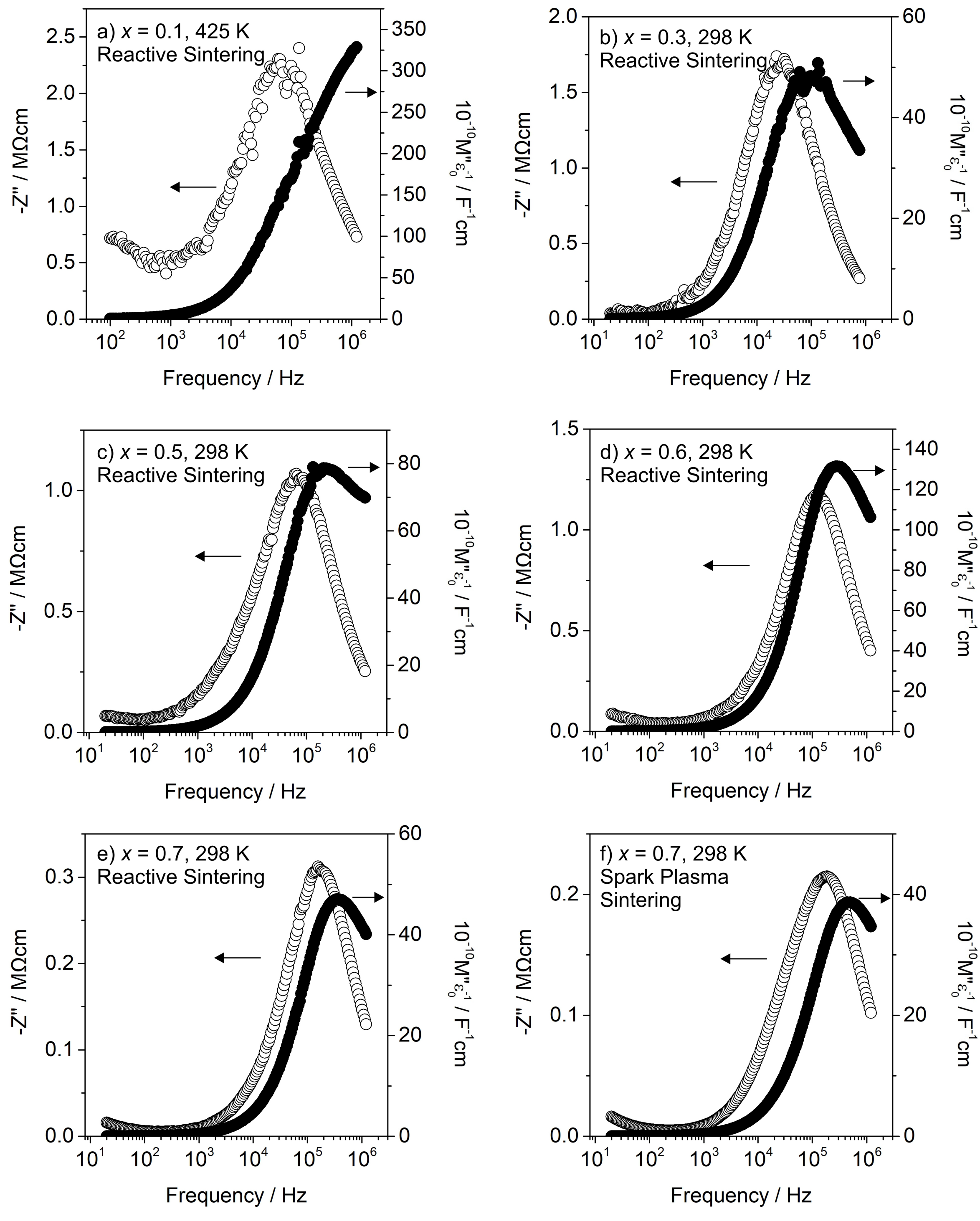

Figure S20A: a) Bode plots for $Li_{6+x}P_{1-x}Si_xO_5Cl$ measured on pellets produced *via* a) $x = 0.1$ reactive sintering (RS), b) $x = 0.3$ RS, $x =$ c) 0.5 RS, d) $x = 0.6$ RS, e) $x = 0.7$ RS, f) $x = 0.7$ spark plasma sintering (SPS).

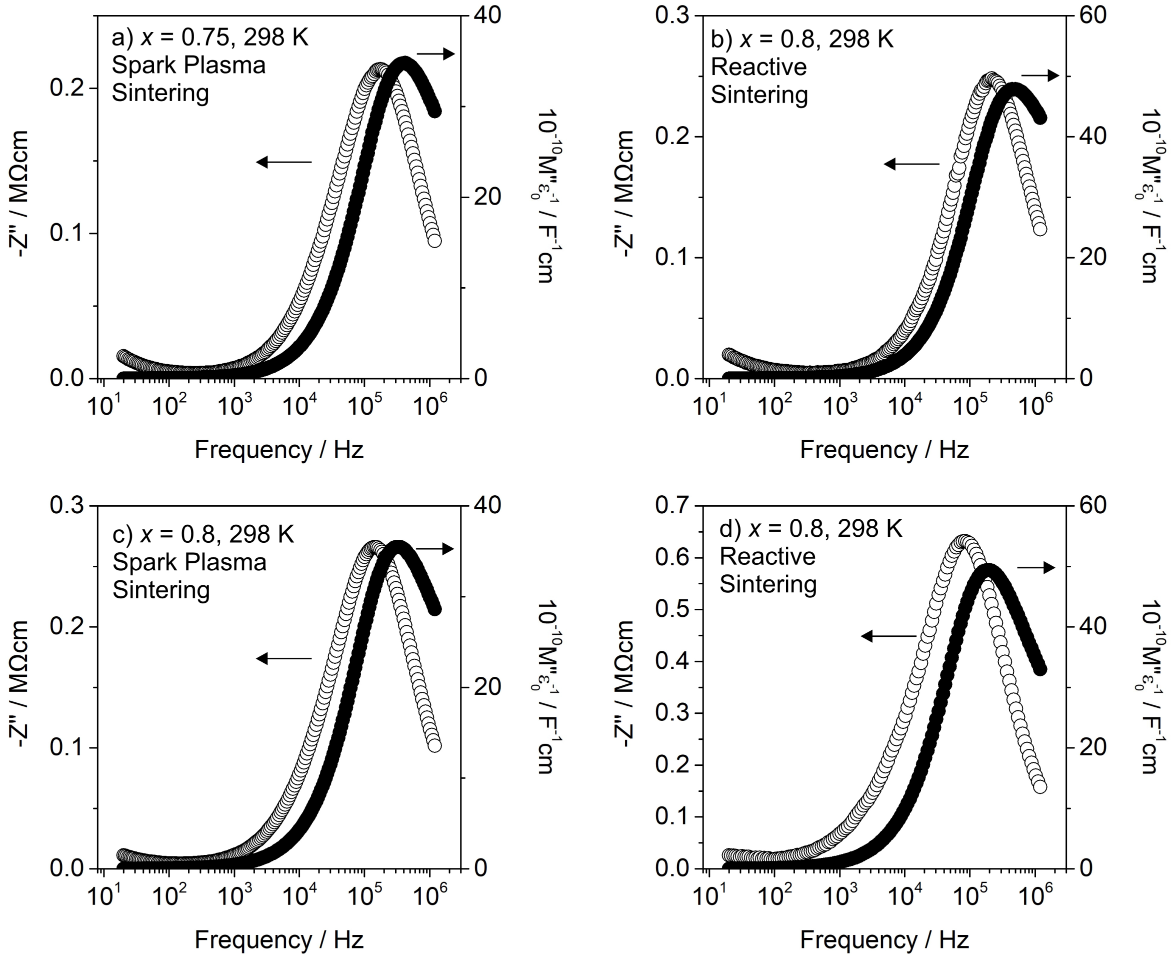

Figure S20B: a) Bode plots for $Li_{6+x}P_{1-x}Si_xO_5Cl$ measured on pellets produced via a) $x = 0.75$ SPS, b) $x = 0.8$ RS, $x =$ c) 0.8 SPS, d) $x = 0.85$ RS.

Table S10: Extracted total ionic conductivities and activation energies for $Li_{6+x}P_{1-x}Si_xO_5Cl$; samples prepared via reactive sintering (RS) ($x = 0.1, 0.3, .5, 0.6, 0.7, 0.75, 0.8, 0.85$) and spark plasma sintering (SPS) ($x = 0.7, 0.75, 0.8$)

**Composition**	**Sintering method**	**Relative density / %**	**Ionic conductivity / S cm^{-1}**	**Activation Energy / eV**
0.1	RS	73.4	~10^{-9}	0.687(13)
0.3	RS	79.0	2.23(1) × 10^{-7}	0.496(4)
0.5	RS	74.5	3.53(4) × 10^{-7}	0.433(4)
0.6	RS	77.1	4.72(3) × 10^{-7}	0.502(3)
0.7	RS	79.7	1.16(4) × 10^{-6}	0.478(11)
0.7	SPS	96.0	1.63(4) × 10^{-6}	0.50(1)
0.75	SPS	95.1	1.82(1) × 10^{-6}	0.522(5)
0.8	RS	85.0	1.54(2) × 10^{-6}	0.491(7)
0.8	SPS	96.5	1.42(5) × 10^{-6}	/
0.85	RS	78.4	4.93(4) × 10^{-7}	0.564(8)

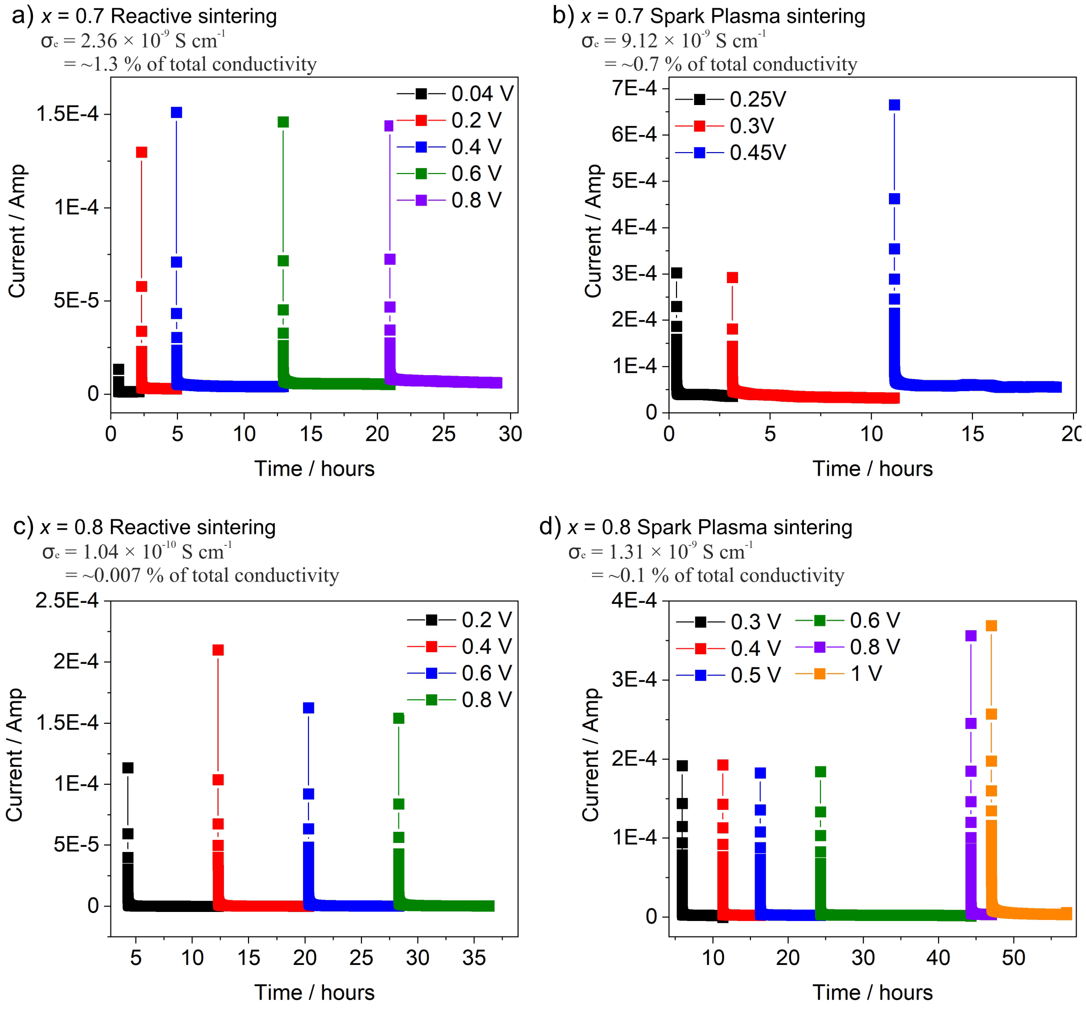

Figure S21: DC Polarisation data for $x = 0.7$ pellets produced through a) reactive sintering and b) spark plasma sintering; measurements from $x = 0.8$ pellets produced through c) reactive sintering, and d) spark plasma sintering. The electronic contribution to total conductivity was calculated to be < 1.5 % for all samples

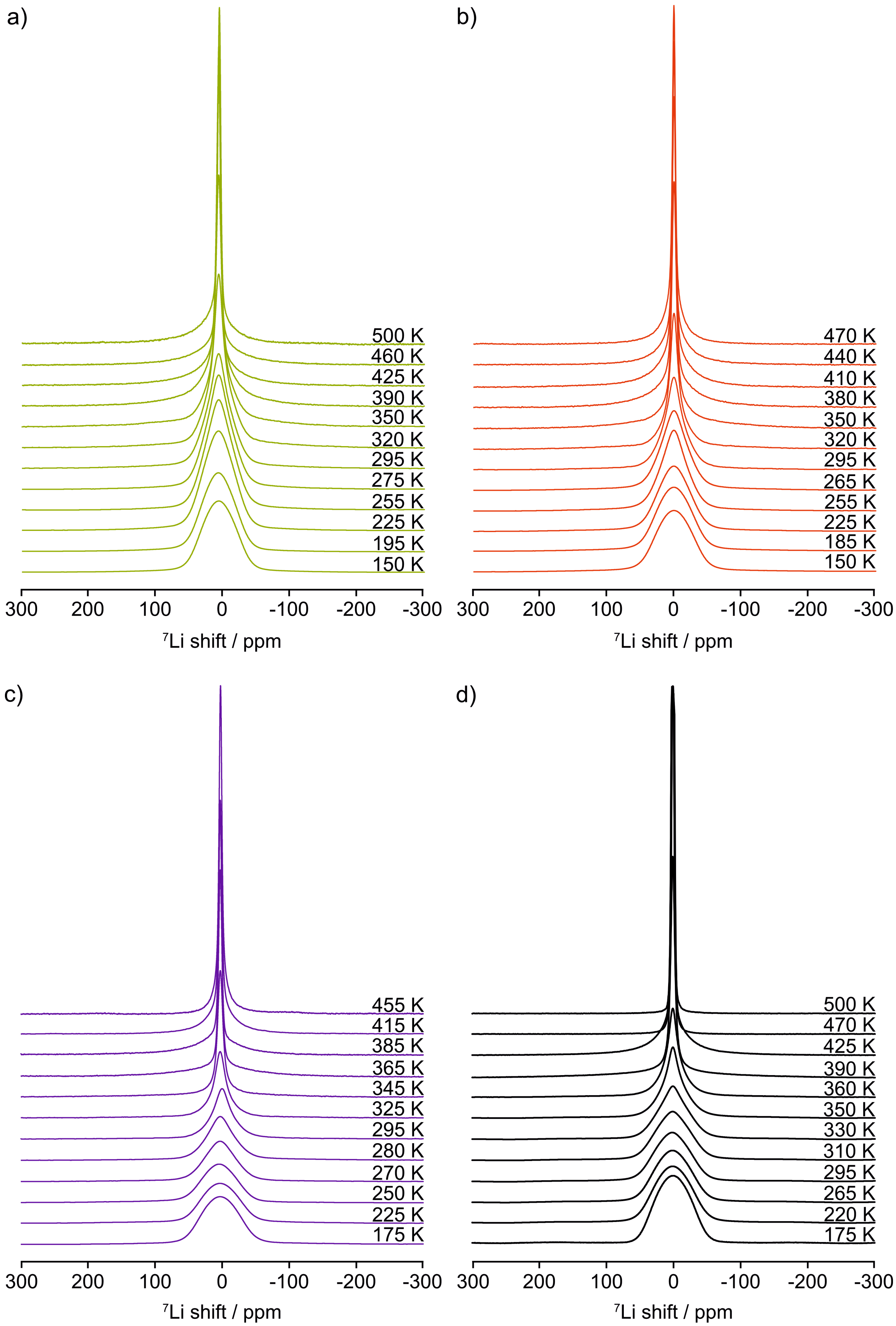

Figure S22: Static ^{7}Li NMR spectra of $Li_{6+x}P_{1-x}Si_xO_5Cl$ for x = a) 0.3 b) 0.5 c) 0.75 and d) 1 measured as a function of temperature.

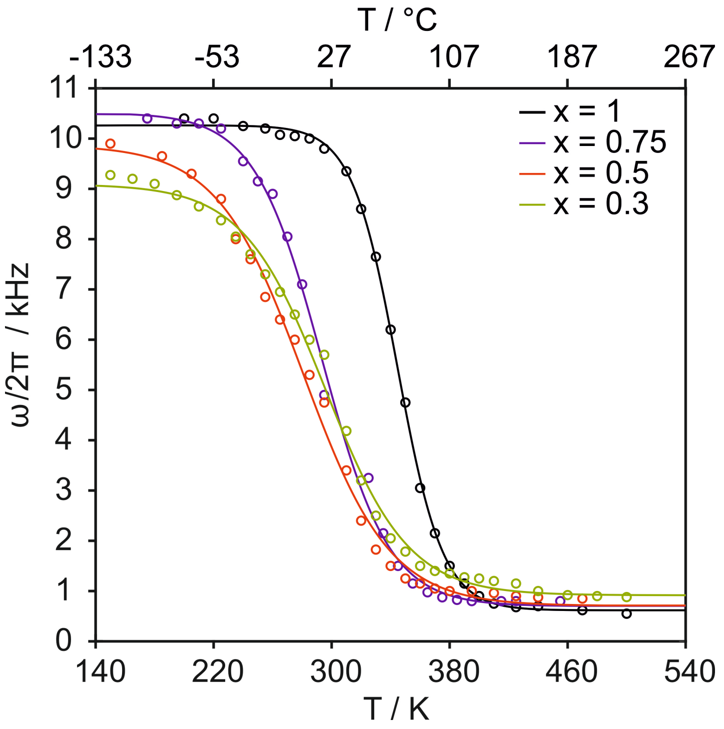

Figure S23: Temperature dependence of the NMR linewidth ω/2π of the ^{7}Li central transition of $Li_{6+x}P_{1-x}Si_xO_5Cl$ (x = 0.3, 0.5, 0.75 and 1). The solid coloured lines are fits to the data based on a sigmoidal regression given in Equation S2 and is used to determine the inflection points of the curves.

The energy of activation obtained through ^{7}Li VT NMR was determined through the correlation of the onset temperature of motional narrowing T_{onset} and E_a:

$$E_a = 1.67 \times 10^{-3} \cdot T_{onset} \qquad \text{(Eq S1)}$$

and the extracted E_a values are summarised in Table S10. The data in Figure S22 was fit to a Boltzmann sigmoid regression curve in order to determine the inflection point of the curve, $T_{inflection}$, where the Li ions have a jump rate, τ^{-1} of the order of rigid lattice regime, $(\omega/2\pi)_{rl}$, this expression takes the form of:

$$\frac{\omega(T)}{2\pi} = \left(\frac{\left(\frac{\omega}{2\pi}\right)_{\infty} - \left(\frac{\omega}{2\pi}\right)_{rl}}{1 + \exp\left(\frac{T_{inflection} - T}{a}\right)} \right) \qquad \text{(Eq S2)}$$

where $\omega(T)/2\pi$ is the central transition linewidth at temperature T, $(\omega/2\pi)_{\infty}$ is the residual linewidth in the fast motional regime and a is a fitting parameter.

Table S11: Comparison of Li ion jump rates τ^{-1} at the respective inflection points extracted from Equation 2, as well as the observed activation energies from Equation 1 obtained from static ^{7}Li VT line narrowing NMR of $Li_{6+x}P_{1-x}Si_xO_5Cl$ (x = 0.3, 0.5, 0.75 and 1).

**Compositions**	**Jump rate τ^{-1} / s^{-1}**	$\boldsymbol{T_{inflection}}$ **/ K**	$\boldsymbol{E_a}$ **/ eV**
0.3	5.7(2) × 10^4	296(3)	0.39
0.5	6.2(2) × 10^4	286(3)	0.34
0.75	6.6(1) × 10^4	290(2)	0.42
1	6.4(1) × 10^4	345(2)	0.52

The NMR lithium jump τ^{-1} values obtained for x = 0.3, 0.5 and 0.75 through Equation 2 increase slightly as Li content increases which is to be expected for decreasing inter and intra-cage jumping distances resulting in stronger ^{7}Li-^{7}Li homonuclear dipolar coupling and higher density of Li spins. The $T_{inflection}$ values for the three compositions are largely similar, however as x increases to 1, $T_{inflection}$ increases significantly, highlighting the reduced Li ion mobility upon the complete ordering of Li sites observed in Li_7SiO_5Cl.

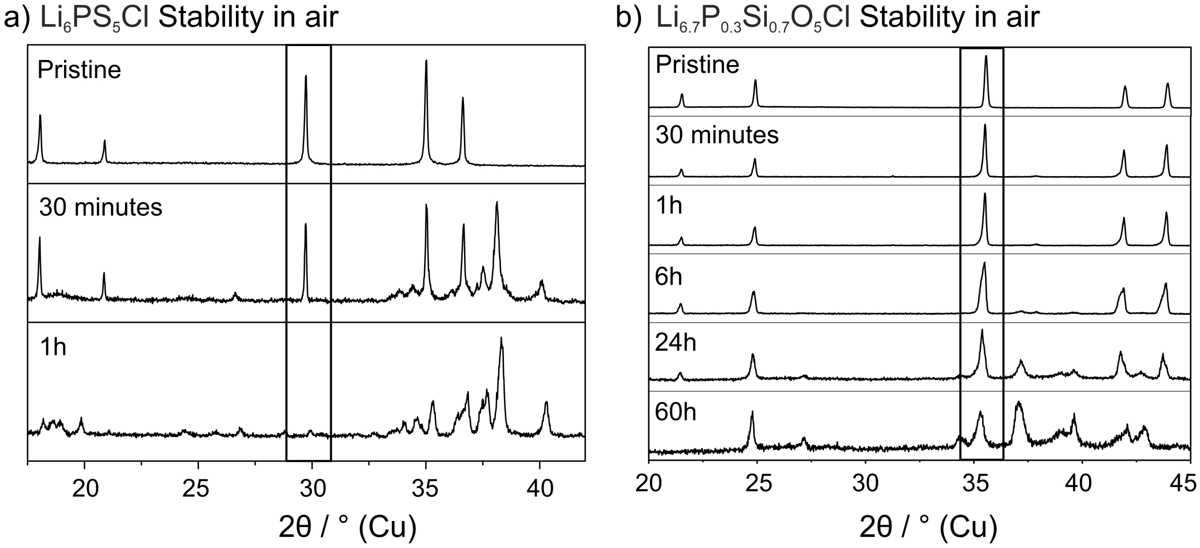

Figure S24: a) Li_6PS_5Cl stability in air for which full decomposition occurs within 1 hour as indicated by the disappearance of cubic argyrodite peaks. b) $Li_{6.7}P_{0.3}Si_{0.3}O_5Cl$ exhibits increased stability in air; decomposition starts after ~3h indicated by broadening of cubic argyrodite peaks and the appearance of Li_2CO_3 impurity peaks.

Table S12: Shortest intra- and inter-cage Li–Li distances in $Li_{6+x}P_{1-x}Si_xO_5Cl$ (x = 0, 0.1, 0.3, 0.5, 0.6, 0.7, 0.75, 0.8, 0.85, 1).

**Intra-cage Distance / Å**									
0	0.1	0.3	0.5	0.6	0.7	0.75	0.8	0.85	1
T5a–T5a	T5–T5								
2.728(9)	2.195(2)	2.269(2)	2.196(3)	2.265(11)	2.106(2)	2.101(13)	2.119(6)	2.087(2)	2.721(2)
**Inter-cage Distance / Å**									
0	0.1	0.3	0.5	0.6	0.7	0.75	0.8	0.85	1
T5a–T5a	T5–T3	T5–T4							
3.087(9)	2.195(2)	1.588(1)	1.575(9)	1.573(4)	1.561(9)	1.606(5)	1.615(8)	1.624(6)	2.229(3)

References:

1. Morscher, A.; Dyer, M. S.; Duff, B. B.; Han, G.; Gamon, J.; Daniels, L. M.; Dang, Y.; Surta, T. W.; Robertson, C. M.; Blanc, F., $Li_6SiO_4Cl_2$: a hexagonal argyrodite based on antiperovskite layer stacking. *Chem. Mater.* **2021,** *33* (6), 2206-2217.

2. Xu, Z.; Stebbins, J. F., Cation dynamics and diffusion in lithium orthosilicate: two-dimensional lithium-6 NMR. *Science* **1995,** *270* (5240), 1332-1334.
